# Associations of screen time, physical activity and neuromuscular fitness with brain structure in adolescents

**DOI:** 10.1016/j.ynirp.2026.100431

**Published:** 2026-11-07

**Authors:** Hannamari Skog, Sara Määttä, Laura Säisänen, Timo A. Lakka, Markus Niiranen, Eero A. Haapala, Mervi Könönen, Juhana Hakumäki

**Affiliations:** a Physiology, Institute of Biomedicine, University of Eastern Finland, Kuopio, Finland; b Institute of Clinical Medicine, Clinical Neurophysiology, University of Eastern Finland, Kuopio, Finland; c Clinical Neurophysiology, Kuopio University Hospital, Kuopio, Finland; d Institute of Clinical Medicine, Neurology, University of Eastern Finland, Kuopio, Finland; e Neuro Center, Neurology, University of Eastern Finland, Kuopio, Finland; f Department of Clinical Physiology and Nuclear Medicine, Kuopio University Hospital, Kuopio, Finland; g Kuopio Research Institute of Exercise Medicine, Kuopio, Finland; h Combinostics Ltd, Tampere, Finland; i Active Life Laboratory, Preventive Health Research Unit, South-Eastern Finland University of Applied Sciences, Mikkeli, Finland; j Sports and Exercise Medicine, Faculty of Sport and Health Sciences, University of Jyväskylä, Jyväskylä, Finland; k Children's Health and Exercise Research Centre, Public Health and Sport Sciences, University of Exeter, Exeter, UK; l Radiology, Institute of Clinical Medicine at the School of Medicine, University of Eastern Finland, Kuopio, Finland; m Imaging Center – Clinical Radiology at Kuopio University Hospital, Kuopio, Finland

**Keywords:** Screen time, Physical activity, Fitness, Brain structure, Adolescence

## Abstract

**Objective:**

To explore the associations of total screen time, smart device use, physical activity, and neuromuscular fitness with brain gray matter volumes in adolescence.

**Methods:**

Brain gray matter volumes were assessed using magnetic resonance imaging in 44 healthy adolescents (25 females and 19 males) aged 16–19 years. Physical activity and sedentary behavior questionnaires were used to assess screen time, including smart device use, and physical activity, including organized sports in sports clubs. Neuromuscular fitness was assessed using a score combining results of a 50-m shuttle-run test and a standing long jump test.

**Results:**

Screen time and smart device use were negatively associated with medial temporal lobe volumes but positively associated with supplementary motor cortex and dorsal lateral prefrontal cortex volumes. Screen time was also positively related to left cuneus volume. Total physical activity was positively associated with right inferior occipital gyrus volume, but the association did not remain statistically significant after correction for multiple testing. Neuromuscular fitness was positively associated with parietal lobe volumes but negatively associated with right hippocampus and left posterior cingulate gyrus volumes, but these associations were no longer statistically significant after correction for multiple testing.

**Conclusion:**

Longer screen time was related to lower medial temporal lobe volumes and larger frontal lobe volumes, whereas higher physical activity and better neuromuscular fitness were associated with larger volumes in the parietal and occipital regions. These findings highlight the complex relationships of screen time and physical activity with brain gray matter volumes in adolescence.

## Highlights

• Higher screen time was associated with lower medial temporal volumes.
• Higher screen time was related to larger frontal lobe volumes.
• Longer use of smart devices was associated with a lower medial temporal volume.
• Longer use of smart devices was associated with larger frontal lobe volume.

## 1 Introduction

Adolescence is a sensitive and neurobiologically critical period for brain maturation. Consequently, the increasing amount of time adolescents spend engaging in sedentary behaviors and digital devices raises substantial concerns (Ortega et al., 2013; Carson et al., 2017; Vanhelst et al., 2023). Recent evidence suggests that adolescents spend substantial amounts of time using screens and smart devices, averaging more than 6.5 h per day (Priftis and Panagiotakos, 2023; Christakis et al., 2025; Verma et al., 2025)**.** In addition, only a few adolescents meet the recommended levels of physical activity (PA) and achieve health-related fitness standards (World Health Organization, 2020). Accordingly, these trends are concerning because increasing evidence suggests that lifestyle factors play an important role in neurodevelopment during adolescence (Juraska, 2024; Skog et al., 2024, 2025). Low PA, excessive screen time (ST), and poor physical fitness (PF) are associated with poor cognitive function and mental health (Whiteman et al., 2016; Haapala et al., 2025). PA, ST, and PF have also been associated with brain structures in children; however, evidence in adolescents is scarce (Zou et al., 2024).

During adolescence and early adulthood, cortical gray matter (GM) volume decreases and white matter volume increases, driving extensive structural and functional brain remodeling (Blakemore et al., 2010; Arain et al., 2013; Juraska, 2024). Cortical GM reduction begins in the dorsal parietal cortices, especially in the superior parietal and intraparietal regions, and then spreads over the anterior regions of the insula and frontal lobe, with cortical thinning progressing into adulthood. The dorsolateral prefrontal cortex (DLPFC) and lateral temporal cortex are the last brain regions to reach full maturity (Gogtay et al., 2004; Casey et al., 2011). Stabilization of cortical thickening and thinning is typically achieved by approximately 15–16 years of age (Casey et al., 2011; Herting and Sowell, 2017). However, recent research suggests that there may be variation in GM development among adolescents (Foulkes and Blakemore, 2018), which may be explained by genetic, epigenetic, and socioeconomic factors (Shi et al., 2024). Although the precise links between structural brain changes and behavioral cognitive performance remain unclear, previous studies have suggested that cognitive development is closely linked to the structural maturation of the brain (Casey et al., 2011; Ing et al., 2019). Most psychopathological disorders, including anxiety, depression, and executive dysfunction symptoms such as ADHD, manifest for the first time in adolescence and have been found to correlate with distinct sets of brain regions and inter-regional connections before behavioral symptoms develop (Ing et al., 2019).

Increasing evidence suggests that excessive ST, defined as at least 2–3 h per day, is associated with poorer mental health and depression (Boers et al., 2019; Santos et al., 2024; Haapala et al., 2025), adverse behaviors such as substance abuse, gambling, and excessive Internet use, and weaker cognitive abilities such as executive functions, attention, and problem-solving in later childhood and adolescence (Manwell et al., 2022; Santos et al., 2024). However, some studies have reported contradictory findings on the association between ST and brain development in children and adolescents (Fung et al., 2023; Hutton et al., 2022; Skog et al., 2025), which may be explained by variations in screen use, type, and content in study populations (Wu et al., 2023; Cristi-Montero et al., 2025). ST is often defined as “time spent passively watching screen-based entertainment, such as TV or mobile devices,” but screen-based activities can vary considerably in their cognitive, social, and behavioral demands. Previous literature has distinguished between active and passive forms of ST (Kaye et al., 2020; Bal et al., 2024; Zhang et al., 2025). Active ST refers to interactive or educational activities that are cognitively or physically stimulating and has been positively associated with executive functions (Bal et al., 2024). In contrast, passive ST involves cognitively and physically inactive behaviors, such as simply watching TV or receiving information without interaction and has been negatively associated with cognitive skills (Kaye et al., 2020; Bal et al., 2024). It has also been suggested that active and passive ST can further be divided to low and high context relevance, i.e., whether the ST content is directly transferable to specific cognitive task or whether it affects domain-specific neural circuits (Zhang et al., 2025). Active and passive ST are also distinct from simple prolonged sitting or device-assessed sedentary time, which could include active or passive ST and be differently associated with brain health outcomes in youth (Zou et al., 2024). Consequently, different forms of screen-based activities may have distinct associations with cognitive function and brain structure, highlighting the importance of considering qualitative aspects of ST in addition to its duration.

Most studies among adolescents or young adults emphasize the negative associations of ST with brain structure (Paulus et al., 2019; Montag and Becker, 2023; Zhang et al., 2025), but the evidence among these age groups is limited compared to evidence among middle-aged or older adults. (Zou et al., 2024). Longer sedentary media time has been associated with reduced volumes in several cortical regions, including the anterior cingulate cortex, precuneus, supplementary motor area, superior parietal cortex, and DLPFC (He et al., 2017; Paulus et al., 2019; Montag and Becker, 2023). However, some studies have reported positive associations between ST and the prefrontal cortex and left insula and bilateral cerebellum volumes in adolescents (He et al., 2017; Paulus et al., 2019). These findings suggest that this relationship can also depend on the screen type (Wu et al., 2023), content usage patterns, or individual variability (Cristi-Montero et al., 2025). The limited and partly inconsistent evidence underscores a significant research gap and the need for further studies investigating the associations between screen time and brain structure in adolescence.

Evidence on the association between PA and GM volume remains mixed. While several studies have reported null findings (Ruotsalainen et al., 2019; Valkenborghs et al., 2019; Rodriguez-Ayllon et al., 2020; Giurgiu et al., 2025), other studies have observed positive associations with GM volumes in the frontal and parietal lobes, anterior cingulate cortex, and hippocampus (Valkenborghs et al., 2019; Gunnell et al., 2021). Notably, Ruotsalainen et al. (2019) reported no association between device-assessed moderate-to-vigorous PA and GM volume in adolescents. Instead of PA, most previous studies have focused on the association between PF (defined as the ability to perform PA) and brain volumes in adolescents (Herting et al., 2016; Ruotsalainen et al., 2019). Better PF has been linked to larger GM volumes in the anterior corpus callosum and cerebellum (Herting et al., 2014; Jalanko et al., 2024), left pallidum, superior frontal cortex (Ruotsalainen et al., 2019), and entorhinal cortex in adolescents and young adults (Whiteman et al., 2016; Verma et al., 2025). A longer ST has also been linked to lower PA levels in adolescents (Vanhelst et al., 2023).

The complex and inconsistent relationships between ST, PA, PF, and brain development in adolescents require further investigation. As brain development in adolescence is sensitive to changes in lifestyle, such as screen use and PA (Manwell et al., 2022; Verma et al., 2025), we investigated the associations of ST, PA, and PF (as neuromuscular fitness encompassing motor skills and muscular strength (Shumway-Cook and Woollacott, 1995)) with GM structure in 44 adolescents. We hypothesized that higher levels of ST would be associated with smaller temporal lobe volume, for which the existing evidence is most consistent, and more tentatively, given the mixed evidence base, with smaller frontal lobe volume (Paulus et al., 2019; Manwell et al., 2022; Verma et al., 2025). Moreover, we hypothesized that higher levels of PA and better neuromuscular fitness are related to larger temporal and frontal lobe volumes.

## 2 Methods

### 2.1 Study design and participants

The data for this cross-sectional study were sourced from the FitBrain study, which took place between 2018 and 2019. Participants were drawn from a group of 277 adolescents who participated in the 8-year follow-up assessments of the Physical Activity and Nutrition in Children (PANIC) study (ClinicalTrials.gov NCT01803776) at the Institute of Biomedicine, University of Eastern Finland (Eloranta et al., 2012). Sixty healthy adolescents, aged 16–19 years (comprising 30 females and 30 males), were invited to join the FitBrain study. These invitations were extended based on the adolescents' performance in the 50-m shuttle run test during the PANIC study's 8-year follow-up, covering a full range of performance levels. Out of those invited, 46 adolescents participated in the study. The participants in the FitBrain study (average 20.6 s, standard deviation [SD] 2.0 s, range 17.7 to 26.1 s) exhibited similar 50-m shuttle run test results to those in the PANIC study (average 20.6 s, SD 2.0 s, range 16.4 to 28.7 s). The FitBrain study excluded individuals with neurological disorders, medications affecting the central nervous system, and typical contraindications for magnetic resonance imaging (MRI) (Pedrosa et al., 2025). Two individuals were excluded from the study due to incidental MRI findings of structural brain abnormalities, leaving a final sample of 44 adolescents, comprising 25 females and 19 males. Among them, one male did not complete the questionnaire, and one female did not participate in the lower limb muscular and motor fitness test, resulting in data from 43 adolescents being available for these measures. The FitBrain participants showed no differences in total physical activity (PA), sedentary time (ST), parental education, weight, or height at the 8-year follow-up of the PANIC study compared to other adolescents in the study. Those who did not attend the 8-year follow-up had a lower body fat percentage (BF%) at baseline (mean difference = −2.1, 95% CI = −3.6 to −0.7) and at the 2-year follow-up (mean difference = −2.7, 95% CI = −4.6 to −1.0), performed worse in the standing long jump at the 2-year follow-up (mean difference = 5.7, 95% CI = 1.6 to 9.8), and had poorer results in the shuttle run test at the 2-year follow-up (mean difference = −0.5, 95% CI = −0.8 to −0.1). They were also more likely to come from families with lower parental education at both baseline (p < 0.001) and the 2-year follow-up. The adolescents included in the analysis were moderately active, and boys accumulated on average 91.2 min (SD = 63.9) and girls 92.5 min (SD = 60.6) of PA per day. Previous research (Ruotsalainen et al., 2019; Jalanko et al., 2024) indicates that a sample size of 45–60 participants are adequate to ensure sufficient statistical power for these analyses.

The PANIC study's design, participants, and protocol have been thoroughly detailed in earlier publications (Lakka et al., 2020; Sallinen et al., 2022). Additionally, the FitBrain study's site selection, recruitment methods, and assessments, along with the approach to handling missing data in these studies, are outlined in the Methods in Supplement (See Methods in Supplement). The Research Ethics Committee of the Hospital District of Northern Savo granted approval for this study (Permit 69/2006 and 295/2015 for the PANIC study and Permit 366/2017 for the FitBrain study). Before the PANIC study commenced, written informed consent was obtained from the children's parents or guardians, and the children themselves gave their assent to participate. Adolescents also provided written informed consent before the 8-year follow-up examinations of the PANIC study and later before participating in the FitBrain study. Both the PANIC and FitBrain studies adhered to the principles outlined in the Declaration of Helsinki. In this article, the term “sex” is used to describe the biological and physiological traits that distinguish men and women, as described in the Sex and Gender Equity in Research (SAGER) guidelines (Heidari et al., 2016).

### 2.2 Assessment of screen time and physical activity

ST and PA were evaluated using the PANIC physical activity and sedentary behavior questionnaire (Haapala et al., 2025; Skog et al., 2024, 2025). The time spent on school or leisure time-related activities during weekdays and weekends was measured and reported in hours and minutes per day. The usage of smart devices was determined by combining the time spent on mobile phones and tablets, also expressed in hours and minutes daily. In this study, total ST included also other forms of ST than smart device use and was computed by adding up the time spent on using mobile phones, tablets, computers, and watching TV and videos. Total ST was presented in hours per day. The PA questionnaire also inquired about participation in organized sports within sports clubs, supervised exercise outside of sports clubs, and unsupervised PA as distinct categories, reported in hours and minutes per day over the past 12 months. Total PA was calculated by summing the time spent on different types of PA, including organized sports in sports clubs, other supervised exercises, and unsupervised PA, and is expressed in minutes per day in this study.

#### 2.2.1 Assessment of body size and composition

Body weight (kg), BF%, and lean mass (LM) (kg) were measured using the Inbody 720® bioimpedance device (Biospace, Seoul, Korea), with the participants having emptied their bladders and wearing only light underwear. Body height (cm) was measured without shoes using a calibrated wall-mounted stadiometer according to standard procedures.

#### 2.2.2 Assessment of neuromuscular fitness (motor fitness and muscular strength)

Motor fitness was evaluated through the 50-m shuttle run test as described by Adam (1988). Participants were instructed to sprint 5 m from one line to another, turn at the line, return to the starting point, and repeat this process until they completed five shuttles. The score for this test was the time taken in seconds, with a longer duration indicating lower performance. Lower limb explosive muscular strength was measured using the standing long jump test (Adam, 1988). Participants stood with their feet together and jumped as far as they could, landing on both feet. The score was the longest jump out of three attempts, measured in centimeters. **Neuromuscular fitness** was calculated by summing the standardized values (z-scores) of the 50-m shuttle run (motor fitness) and standing long jump tests (muscular strength). Z-scores were calculated using the mean and standard deviation of the entire study sample. The scores from the 50-m shuttle run were multiplied by −1 so that higher score represented better performance. The 50-m shuttle run reflects motor fitness components such as speed, agility, and coordination, whereas the standing long jump reflects lower-body muscular power. Consistent with current physical fitness frameworks, neuromuscular fitness was considered a multidimensional construct encompassing both motor fitness and muscular strength/power components (Riebe et al., 2026).

#### 2.2.3 Assessment of brain structure

Participants underwent scanning with a 3.0 T MRI scanner (Philips Achieva TX; Philips Healthcare, Eindhoven, The Netherlands) with an 8-channel SENSE head coil. T1-weighted images used for VBM analysis, were scanned in the sagittal plane with 3D turbo field echo sequence (TR = 8.1ms, TE =3.7ms, 190 slices, FOV 250 × 250 mm^2^, matrix 256 × 256, voxel size 0.94 mm × 0.94 mm × 1 mm). Additionally, for assessing potential parenchymal pathology, a T2W-FLAIR sequence was performed (TR 11000 ms, TE 120 ms, TI 2800 ms, 29 transaxial slices, FOV 240 × 240 mm^2^, matrix 560 × 560, 4 mm thickness, 1 mm gap, pixel size 0.43 mm × 0.43 mm). An experienced neuroradiologist reviewed all images for potential abnormalities. If any incidental findings were identified, the participant was referred for further examination. The commercial cNeuro® cMRI software (Combinostics Ltd., Tampere, Finland), was used for voxel-based morphometry (VBM) analysis of MRI images. The software employs an MRI-based structural template and a multi-atlas segmentation algorithm to automatically quantify the volumes of 102 cortical brain regions and 10 subcortical gray matter structures from T1-weighted and FLAIR images (Lötjönen et al., 2010). In addition, white matter volumes were assessed separately for both hemispheres, and specific lobar and gyral volumes were derived by combining anatomical templates (Lötjönen et al., 2010). Multiple anatomical biomarkers, including total brain, gray matter, white matter, cerebellar, cerebrospinal fluid (CSF), supratentorial cortical, and deep gray matter volumes, were extracted from the MRI images. The cNeuro® cMRI software also provides age-, sex-, and head size-adjusted normative values based on a reference database comprising 1923 individuals, as described previously (Cole and Green, 1992; Buckner et al., 2004; Lötjönen et al., 2010; Koikkalainen et al., 2016).

#### 2.2.4 Other assessments

The parents were asked to report their highest completed or ongoing educational degrees, categorized as vocational school or less, polytechnic, or university, and the degree of the more educated parent was used in the analyses.

### 2.3 Statistical analyses

All statistical analyses were conducted using IBM SPSS statistical analysis software (version 25.0, for Macintosh IBM Corporation, Armonk, NY, USA). The normality of variable distributions was assessed using the Kolmogorov-Smirnov test and visually through histograms. Differences between females and males were analyzed using the independent samples *t*-test or the Mann-Whitney *U* test. Due to skewed distributions, we applied a logarithmic transformation to the smart device use and total PA variables prior to conducting linear regression analyses. Initially, Pearson's correlation analysis was employed to select variables for further examination (see Supplementary Table 1). Subsequently, we analyzed these associations from the correlation analyses with p < 0.01 using linear regression analyses, adjusting for age, sex, head circumference, and BF% to explore the relationships among ST, PA, neuromuscular fitness, and MRI variables. BF% was included in the models because previous research has indicated a connection between BF% and brain structures, ST, PA, and physical fitness (Silva et al., 2021; Haghjoo et al., 2022). Age, sex, and head circumference were entered into the linear regression models in the first step, while the measures of ST, PA, and neuromuscular fitness were added separately in the second step. BF% was incorporated into the linear regression models in the third step. The standardized regression coefficients b and the corresponding P-values are reported for each factor. Differences and associations with p-values lower than 0.05 were considered statistically significant. All linear regression models were evaluated for linearity using scatter plots and for normal distribution and homoscedasticity of residuals using normal probability plots and residual scatter plots, respectively. Additionally, we assessed the potential for multicollinearity in our models using the variance inflation factor (VIF) and tolerance values, with a threshold value of 5 for the VIF and 0.2 for tolerance. Finally, we examined whether sex moderated the associations of total ST, smart device use, total PA, or neuromuscular fitness with GM volumes by including the corresponding sex × predictor interaction terms in the general linear models. Multiple comparisons were controlled using the Benjamini-Hochberg false discovery rate (FDR) procedure. An FDR-adjusted p-value < 0.10 was considered statistically significant to balance the risks of false-positive and false-negative findings. A total of 72 tests were included in the FDR correction.

## 3 Results

### 3.1 Characteristics of adolescents

The characteristics of the 44 adolescents (25 females and 19 males) are presented in Table 1. Males were taller and heavier, had a lower BF%, and spent more time using computers than females. Males were also faster in the 50-m SRT and jumped a longer distance in the SLJ test than females (Table 1).

**Table 1:** Characteristics of adolescents.

	All (n = 44)	Females (n = 25)	Males (n = 19)	p-value
**Age (yr)**	17.9 ± 0.8 (16.3−19.1)	17.8 ± 0.8 (16.3−18.7)	18.0 ± 0.8 (16.4−19.1)	0.289
**Height (cm)**	172.0 ± 8.7	165.8 ± 4.8	179.4 ± 6.4	**<0.001**
**Weight (kg)**	67.0 ± 11.3	60.8 ± 8.4	75.2 ± 9.2	**<0.001**
**Body mass index (kg/m^2^)**	22.7 ± 2.9	22.0 ± 3.0	23.4 ± 2.7	0.137
**Lean mass (kg)**	52.8 ± 11.0	45.1 ± 6.1	62.9 ± 7.0	**<0.001**
**Body fat percentage (%)**	22.0 ± 7.5	25.6 ± 5.4	16.1 ± 6.3	**<0.001**
**Head circumference (cm)**	56.9 ± 1.8	55.9 ± 1.1	58.2 ± 1.6	**<0.001**
**Total screen time, h/d**^a^	9.2 ± 4.2 (2.5–19.0)	8.4 ± 4.0 (2.5–18.8)	10.32 ± 4.4 (3.8–19.0)	0.147
**Using smart devices (h/d)**^a^	5.2 ± 3.5 (0.0–17.5)	5.7 ± 3.8 (0.5–17.5)	4.3 ± 3.1 (0.0–13.0)	0.200
**Using a computer (h/d)**^a^	3.0 ± 3.0 (0.0–12.0)	2.1 ± 1.5 (0.0–4.5)	4.3 ± 4.0 (0.0–12.0)	**0.036**
**Watching television and videos (h/d)**^a^	2.5 ± 1.9 (0.0–8.0)	2.2 ± 1.8 (0.0–6.0)	2.9 ± 2.1 (0.0–8.0)	0.261
**Total physical activity, min/d**^a^	92.0 ± 61.2 (10.2–252.5)	92.5 ± 60.6 (26.8–252.5)	91.2 ± 61.2 (10.5–201.2)	0.946
**Organized sports (min/d)**^a^	13.5 ± 22.4 (0.0–79.1)	18.2 ± 24.9 (0.0–79.1)	7.0 ± 16.9 (0.0–63.1)	0.107
**Supervised exercise (min/d)**^a^	4.6 ± 12.0 (0.0–66.4)	5.3 ± 8.9 (0.0–30.3)	3.7 ± 15.7 (0.0–66.4)	0.669
**Unsupervised physical activity (min/d**^a^**)**	56.2 ± 57.8 (0.0–228.7)	51.7 ± 58.5 (0.0–228.5)	62.5 ± 57.9 (2.1–176.2)	0.549
**Neuromuscular fitness (score)**^a^	−0.05 ± 1.0 (−2.7–1.6)	−0.02 ± 1.0 (−1.7–1.5)	−0.08 ± 1.0 (−2.7–1.6)	0.833
**50-m shuttle run (s)**	20.5 ± 1.9 (16.7–25.1)	21.4 ± 1.7 (19.1–25.1)	19.4 ± 1.5 (16.7–22.5)	**<0.001**
**Standing Long Jump (cm)**^a^	196.5 ± 36.2 (127–273)	173.6 ± 24.7 (127–210)	224.2 ± 27.7 (152–273)	**<0.001**
**Parental education (n, %)**				0.672
**vocational school or less**	6, 14.3	3, 12.5	3, 16.7	
**polytechnic**	15, 33.3	7, 29.2	8, 38.9	
**university**	23, 52.4	15, 58.3	8, 44.4	

Data are presented as means ± standard deviations for normally distributed variables and as medians (minimum–maximum) for non-normally distributed variables. Differences between females and males were analyzed using the independent samples *t*-test or the Mann-Whitney *U* test, as appropriate. P-values < 0.05 are shown in bold.

a n = 43 due to missing data for one participant.

### 3.2 Associations of screen time, smart device use, physical activity and neuromuscular fitness with brain structure in adolescence

#### 3.2.1 Temporal lobe segments

Total ST was negatively associated with medial temporal volumes (total, R, L) (β = −.527, 95% CI -.801 to- .211, p = .001; β = −.467, 95% CI -.770 to −.136, p = .005; β = −.371, 95% CI -.658 to −.046, p = .024), after adjustment for age, sex, head circumference, and BF% (Table 2; Fig. 1). Smart device use was negatively associated with medial temporal volumes (total, R, L) (β = −.529, 95% CI -.790 to −.206, p = .001; β = −.364, 95% CI -.671 to −.015, p = .030; β = −.489, 95% CI -.746 to −.176, p = .002). These associations remained significant after FDR correction. Neuromuscular fitness was negatively associated with right hippocampal volume (β = −.450, 95% CI -.824 to −.067, p = .022), but it did not remain significant after the FDR-correction (corrected p = .242). There were no associations between total PA and temporal lobe segment volumes. Sex did not moderate the associations of ST, smart device use, PA, or physical fitness with temporal lobe segment volumes (p for all interactions ≥ .117).

**Table 2:** Associations of screen time, smart device use, physical activity, and neuromuscular fitness with brain volumes.

Brain volumetry	Total screen time (h/d)				Smart device use (h/d)				Total physical activity (min/day)				Neuromuscular fitness (score)			
	β (95% CI)	p	ΔR^2^	p_FDR_	β (95% CI)	p	ΔR^2^	p_FDR_	β (95% CI)	p	Δ R^2^	p_FDR_	β (95% CI)	p	Δ R^2^	p_FDR_
**TEMPORAL LOBE**																
Medial temporal total	**−.527 (−0.801, −0.211)**	**.001**	.225	**†.022**	**−.529 (−0.790, −0.206)**	**.001**	.222	**†.004**	.170 (−0.133, 0.497)	.296	.035	.523	.083 (−0.327, 0.490)	.683	.004	.928
Medial temporal R	**−.467 (−0.770, −0.136)**	**.005**	.180	**†.055**	**−.364 (−0.671, −0.015)**	**.030**	.106	**†.066**	.200 (−0.116, 0.532)	.223	.042	.523	.023 (−0.390, 0.435)	.911	<.001	.928
Medial temporal L	**−.371 (−0.658, -0.046)**	**.024**	.109	**†.096**	**−.489 (−0.746, −0.176)**	**.002**	.191	**†.007**	.054 (−0.245, 0.372)	.734	.004	.942	.102 (−0.297, 0.497)	.605	.006	.928
Hippocampus R	.016 (−0.302, 0.372)	.928	<.001	.928	−.083 (−0.416, 0.273)	.626	.004	.724	−.064 (−0.354, 0.308)	.682	<.001	.860	**−.450 (−0.824, −0.067)**	**.022**	.128	.242
**FRONTAL LOBE**																
Supplementary motor cortex total	**.399 (0.075, 0.792)**	**.012**	.133	**†.066**	**.542 (0.243, 0.815)**	**.001**	.252	**†.000**	.156 (−0.167, 0.467)	.318	.022	.523	.124 (−0.263, 0.507)	.522	.009	.928
Supplementary motor cortex L	**.409 (0.097, 0.714)**	**.010**	.144	**†.066**	**.587 (0.316, 0.855)**	**.001**	.307	**†.000**	.149 (−0.167, 0.461)	.339	.021	.523	.188 (−0.196, 0.570)	.329	.027	.904
Dorsolateral prefrontal cortex total	**.343 (0.021, 0.661)**	**.035**	.102	**†.096**	**.493 (0.199, 0.748)**	**.001**	.217	**†.004**	.217 (−0.096, 0.528)	.164	.045	.523	−.052 (−0.444, 0.323)	.790	.002	.928
Dorsolateral prefrontal R	**.355 (0.024, 0.674)**	**.031**	.107	**†.096**	**.500 (0.195, 0.793)**	**.001**	.219	**†.008**	.165 (−0.159, 0.484)	.298	.025	.523	−.071 (−0.465, 0.316)	.722	.003	.928
Dorsolateral prefrontal L	.311 (−0.005, 0.630)	.056	.085	.114	**.456 (0.167, 0.753)**	**.003**	.190	**†.008**	.242 (−0.062, 0.548)	.117	.057	.523	−.025 (−0.416, 0.343)	.897	<.001	.928
Superior frontal gyrus total	.259 (0.086, 0.587)	.123	.054	.225	**.477 (0.159, 0.770)**	**.003**	.194	**†.008**	.284 (−0.035, 0.593)	.071	.076	.520	−.296 (−0.683, 0.078)	.132	.059	.539
Opercular part of the inferior frontal gyrus R	.232 (0.104, 0.565)	.169	.046	.286	**.414 (0.109, 0.727)**	**.010**	.157	**†.024**	.204 (−0.112, 0.522)	.119	.041	.523	.136 (−0.262, 0.534)	.495	.011	.928
**PARIETAL LOBE**																
Parietal lobe L	−.204 (−0.503, 0.116)	.198	.032	.308	.286 (−0.571, 0.026)	.063	.066	.126	.011 (−0.283, 0.314)	.942	<.001	.942	**.508 (0.165, 0.821)**	**.004**	.164	.088
Supramarginal gyrus L	−.150 (−0.491, 0.192)	.381	.019	.490	−.022 (−0.364, 0.318)	.894	<.001	.936	.304 (−0.006, 0.614)	.054	.090	.520	.292 (−0.103, 0.681)	.139	.053	.539
Posterior cingulate gyrus L	−.109 (−0.456, 0.213)	.513	.012	.627	−.136 (−0.487, 0.171)	.404	.025	.522	.073 (−0.251, 0.384)	.641	.004	.783	**−.379 (−0.746, −0.005)**	**.045**	.091	.330
**OCCIPITAL LOBE**																
Inferior occipital gyrus R	.212 (−0.132, 0.543)	.210	.037	.308	.296 (−0.037, 0.615)	.070	.078	.128	**.360 (−0.054, 0.660)**	**.021**	.125	.462	.144 (−0.255, 0.541)	.469	.013	.928
Middle occipital gyrus R	−.043 (−0.393, 0.296)	.803	.002	.841	.011 (−0.338, 0.344)	.949	<.001	.949	−.131 (−0.457, 0.189)	.414	.017	.607	.072 (−0.331, 0.471)	.720	.003	.928
Cuneus total	.289 (−0.013, 0.581)	.057	.070	.114	.165 (−0.145, 0.462)	.279	.022	.402	−.152 (−0.440, 0.136)	.299	.023	.523	−.045 (−0.413, 0.318)	.806	.001	.928
Cuneus L	.**341 (0.023, 0.651)**	**.035**	.099	**†.096**	.259 (−0.065, 0.572)	.108	.057	.168	−.115 (−0.428, 0.196)	.464	.013	.638	.018 (−0.379, 0.406)	.928	<.001	.928

The data are standardized regression coefficients β with corresponding 95% confidence intervals (95% CIs) and their p-values from linear regression analyses adjusted for age, sex, head circumference, and body fat percentage (%) before (p < 0.05 for statistical significance) and after false discovery rate (FDR)-correction = p_FDR_ (q < 0.10 for statistical significance). Standardized regression coefficients β, 95% Cis, and *P*-values for statistically significant (p < 0.05) associations are bolded. † = Statistically significant after FDR-correction using the Benjamini-Hochberg procedure (q < 0.10). ΔR^2^ values smaller than .001 are reported as < .001.

The neuromuscular fitness score was calculated as a composite score by combining the z-scores from the 50-m shuttle run test (motor fitness) and the standing long jump test (muscular strength).

Abbreviations: L = left; R = right.

**Fig. 1:**
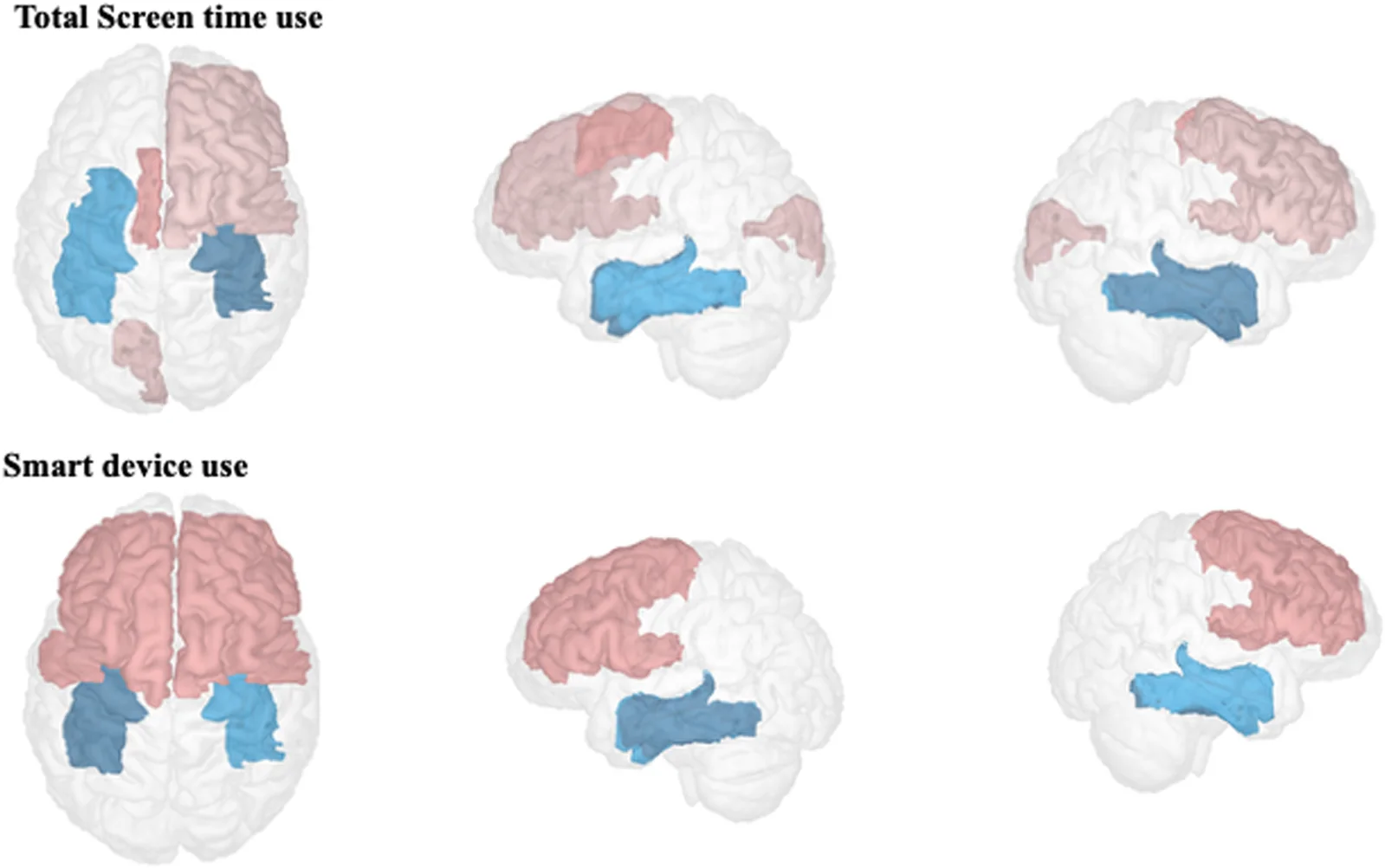
3D visualization of the associations of total screen time and smart device use with brain volume. The results considering physical activity and neuromuscular fitness are not depicted due to their weak and inconsistent association with brain volumes. Brain regions are color-coded as follows: red hue indicating positive and blue negative associations (p < 0.05) and darker color shading denoting higher significance (p < 0.01). All these associations remained significant after FDR correction. Total Screen time use (upper panel): right dorsal lateral prefrontal cortex= light red, left supplementary motor cortex= dark red, right medial temporal cortex= dark blue, left medial temporal cortex= light blue, left cuneus= light red. Smart device use (lower panel): left and right dorsal lateral prefrontal=dark red, right opercular part of the inferior frontal gyrus= dark red, total superior frontal gyrus= dark red, left supplementary motor cortex= dark red, right medial temporal cortex= light blue, left medial temporal cortex= dark blue.

#### 3.2.2 Frontal lobe segments

Total ST was positively associated with supplementary motor cortex (SMC) volumes (total, L) (β = .399, 95% CI .075 to .792, p = .012; β = .409, 95% CI .097 to .714, p = .010) and dorsal lateral prefrontal volumes (total, R) (β = .343, 95% CI .021 to .661, p = .035; β = .355, 95% CI .024 to .674, p = .031). Smart device use was positively associated with SMC volumes (total, L) (β = .542, 95% CI .243 to .815, p = .001; β = .587, 95% CI .316 to .855, p = .001), dorsal lateral prefrontal volumes (total, R, L) (β = .493, 95% CI .199 to .748, p = .001; β = .500, 95% CI .195 to .793, p = .001, β = .456, 95% CI .167 to .753, p = .003), the superior frontal gyrus (SFG) (total) (β = .477, 95% CI .159 to .770, p = .003), and volumes of the opercular part of the inferior frontal gyrus (R) (β = .414, 95% CI .109 to .727, p = .010). All these associations remained significant after FDR correction. There were no associations between total PA or neuromuscular fitness and frontal lobe segment volumes. Sex did not moderate the associations of ST, smart device use, PA, or physical fitness with frontal lobe segment volumes (p for all interactions ≥.138).

#### 3.2.3 Parietal lobe segments

Neuromuscular fitness was positively associated with parietal lobe volume (β = .508, 95% CI .165 to .821, p = .004) and this association remained significant after FDR correction. Neuromuscular fitness was negatively associated with posterior cingulate gyrus volume (β = −.379, 95% CI -.746 to −.005, p = .045), but this association did not remain significant after the FDR-correction (corrected p = .330). There were no associations between total ST or smart device use and parietal lobe segments. Sex did not moderate the associations of ST, smart device use, PA, or physical fitness with parietal lobe segment volumes (p for all interactions ≥.197).

#### 3.2.4 Occipital lobe segments

Total ST was positively associated with the left cuneus volume (β = .341, 95% CI .023 to .651, p = .035). Total PA was positively associated with the right inferior occipital gyrus volume (β = .360, 95% CI -.054 to .660, p = .021), but the association did not remain after the FDR-correction (corrected p= .462). There were no associations between total smart device use or neuromuscular fitness and occipital lobe segments. Sex did not moderate the associations of ST, smart device use, PA, or physical fitness with occipital lobe segment volumes (p for all interactions ≥.081).

## 4 Discussion

In this cross-sectional study of Finnish adolescents, we examined the associations between total ST, PA, smart device use, and neuromuscular fitness with whole-brain MRI volumetry. We found that higher levels of self-reported ST were associated with lower medial temporal volumes but larger volumes of the SMC, DLPFC, and left part of the cuneus. Smart device use was associated with lower medial temporal volumes but larger volumes of the SMC, DLPFC, opercular part of the inferior frontal gyrus, and superior frontal gyrus. Total PA was only associated with larger volumes of the right inferior occipital gyrus. Better neuromuscular fitness was associated with a larger parietal lobe but a lower volume of the right hippocampus and left posterior cingulate gyrus.

The initial assumption was that higher ST levels would be negatively associated with frontal and temporal lobe volumes. We found that higher ST and longer time spent using smart devices were associated with lower medial temporal volumes. These findings are partially consistent with prior studies, where excessive ST (e.g., Internet use, problematic smartphone use, online gaming addiction) in adolescence was associated with lower GM volume in the right SFG, bilateral amygdala, and right ventral striatum (Wang et al., 2016; He et al., 2017). Although there are differences compared to the results of previous studies, a consistent finding in both our and previous studies is the inverse association with the temporal lobes. ST has also been associated with lower GM volumes in medial temporal areas (Paulus et al., 2019) and lower volumes in the left anterior insula, inferior temporal, and parahippocampal cortex (Horvath et al., 2020) in adolescents. The temporal cortex is also a late-maturing area and is particularly sensitive to environmental exposure (Gogtay et al., 2004). Our results, together with prior findings (Wang et al., 2016; He et al., 2017; Paulus et al., 2019; Horvath et al., 2020; Ding et al., 2023), underline that higher levels of ST and longer daily use of smart devices are negatively associated with temporal lobe volumes in adolescence.

Contrary to our hypothesis, we found that higher levels of total ST were associated with larger volumes in the SMC and DLPFC (in the frontal lobe). In addition, longer self-reported ST was associated with larger volumes in the left part of the cuneus in the occipital lobe. Similarly, longer smart device use was associated with larger SMC and DLPFC volumes. These discrepancies could be partly explained by the complex role of ST types in brain development (Bal et al., 2024). The frontal lobe is a large cortical region that includes both the primary motor cortex and supplementary motor areas and encompasses the prefrontal cortex, which is critically involved in higher-order executive functions such as planning, organizing, attention regulation, and emotional control (Miller and Cohen, 2001; Verma et al., 2025). Additionally, the DLPFC is responsible for executive functions such as working memory, planning, decision-making, and cognitive control (Chan et al., 2008; Weng et al., 2013). In this context, active ST, which involves mentally or physically engaging with a device and focusing on creation, problem-solving, or interaction, may also be positively associated with frontal lobe and DLPFC volumes (Kaye et al., 2020; Bal et al., 2024). Paulus et al. (2019) showed that gaming activity had positive associations with prefrontal cortex volume, but social media use was negatively associated with hippocampal thickness and temporal lobe volume**.** Mukherjee et al. (2025) found that in 20-year-old young adults who were active videogame players had greater cortical thickness in right inferior and superior parietal, supramarginal, and precuneus cortices than their less actively playing counterparts. In contrast to our findings, longer smartphone use has been associated with lower GM volumes in the SFG in young adults (Wang et al., 2021; Montag and Becker, 2023). However, as Mukherjee et al. (2025) investigated cortical thickness and we assessed GM volumes, the results are not directly comparable.

The positive associations between longer smart device use and volumes of the opercular part of the inferior frontal gyrus and SFG may also be explained by differences in ST or different responses of lobes to ST. The opercular part of the IFG belongs to Broca's area/Brodmann area 44 and is similarly involved in cognitive control, such as inhibition and working memory, language production, and motor learning (Lehman and Hoepner, 2021). The SFG plays an important role in motor and cognitive brain networks and has a significant role in working memory, inhibitory control, and decision-making, and its volumetric decreases have been shown during the progression of Alzheimer's disease (Wang et al., 2024). The contrasting findings between the previous studies and our study may be due to differences in the composition of ST measures. The measure of ST in our study included both active and passive forms of ST, whereas He et al. (2017) examined the association between excess social media use (not total ST) and brain morphology. Also Paulus et al. (2019) assessed screen media activity using multiple recreational screen-based behaviors, including television/video viewing, video gaming, social media use, texting, video chatting, and internet use, but the study did not distinguish between active and passive ST. For example, smart device use could include activities such as digital gaming or watching educational content with lower and higher mental activation, respectively, with positive associations with brain structures (Zhang et al., 2025). However, these hypotheses of passive and active ST use require further investigation.

In the parietal lobes, only better neuromuscular fitness (measured by SLJ and SRT) was associated with larger volumes. This region plays a major role in sensory and attention processing (Lehman and Hoepner, 2021). In prior studies, PF has been associated with a larger cerebellar Crus I lobule GM volume (Jalanko et al., 2024) and larger volumes of the left hippocampus and right rostral middle frontal cortex in adolescence (Herting and Nagel, 2012). Moreover, cardiorespiratory fitness was positively related to entorhinal cortex GM volume (Whiteman et al., 2016) in young adults (18–30 years old). We also found that better neuromuscular fitness was associated with lower volumes of the right hippocampus and left posterior cingulate gyrus; however, these associations did not remain after FDR-correction and therefore they should be interpreted as exploratory findings that require confirmation in further studies. The inverse associations between neuromuscular fitness and right hippocampal and posterior cingulate volumes were unexpected, given that previous studies (Chaddock et al., 2010; Herting and Nagel, 2012) have generally reported positive associations between cardiorespiratory fitness and hippocampal volume. Moreover, prior evidence has also suggested that cardiorespiratory fitness, rather than motor fitness, is associated with hippocampal volume (Esteban-Cornejo et al., 2017). Therefore, it is possible that hippocampal volume is more strongly related to cardiorespiratory fitness than to neuromuscular fitness. In addition, neuromuscular fitness and cardiorespiratory fitness may reflect partly distinct physiological and neurobiological processes. As these associations did not remain statistically significant after FDR correction, they should be interpreted with caution and may represent chance findings rather than robust evidence of an inverse relationship between neuromuscular fitness and brain structure.

Similarly, in the occipital lobe, we found that a longer self-reported total PA was associated with larger volumes of the right inferior occipital gyrus; however, this association should be interpreted cautiously, as it did not remain after the FDR-correction. There are a few possible explanations for why total PA had no statistically significant association with brain volumetry. First, most previous studies in adults have reported that only moderate- or high-intensity exercise is associated with increased volumes of the hippocampus and several cortical regions (Erickson et al., 2011; Fox et al., 2022; Jemni et al., 2023). Positive effects of PA have mostly been observed in the brain's cognitive and motor regions, suggesting that the effects of PA are not uniform across the brain (Fox et al., 2022). In adolescents, most studies have not found an association between self-reported PA and GM volumes (Herting et al., 2016; Ruotsalainen et al., 2019). However, some previous reviews have suggested that PA may influence GM volumes in the frontal and parietal lobes in children and adolescents (Valkenborghs et al., 2019; Gunnell et al., 2021). Habitual PA among adolescents may be of lower intensity to cause sufficient stimulus influencing brain volumes (Toivo et al., 2023). The associations between PA and brain volumes have been less studied in adolescence, and its role in GM volume has been partly mixed (Valkenborghs et al., 2019; Gunnell et al., 2021). Furthermore, direct comparison with prior studies is challenging, mainly due to the small sample size, variation in research designs and measurement methods, and different age groups (Valkenborghs et al., 2019; Gunnell et al., 2021).

Previous studies (Gogtay et al., 2004; Tamnes et al., 2017) suggest that distinct aspects of brain anatomy follow different developmental trajectories during adolescence. The rate of GM development and maturation differs between individuals due to genetic or epigenetic variations (Shi et al., 2024), and ST and PA behaviors change during maturation. These individual and maturational differences highlight the complexity of the associations between ST and brain development in adolescence, along with the context-dependency of ST (Montag and Becker, 2023; Wu et al., 2023; Hutton et al., 2024). Nevertheless, studies have repeatedly shown that longer ST use is negatively associated with the prefrontal regions, which are essential for attention, executive functioning, emotional regulation, and language processing (Manwell et al., 2022; Ding et al., 2023). Despite some positive associations with ST, our findings—together with prior research—indicate that higher levels of ST and longer smart device use may have predominantly negative consequences for some brain structures during adolescence.

Taken together, the relationship between adolescents’ daily habits—ST, PA, and other types of sedentary behavior–with brain structure is complex (Hutton et al., 2024; Sanders et al., 2024; Cristi-Montero et al., 2025). The findings of our study suggest that, in addition to the amount and content of digital media use/ST and PA, the qualitative aspects of these behaviors may be important consideration in their associations with brain structure during adolescence. In particular, the finding that a higher ST in adolescence is negatively associated with brain lobe regions implicated in memory disorders raises concerns. Therefore, further research is warranted to elucidate the nature of these associations and their implications for adolescent brain development.

## 5 Strengths and limitations

The strengths of our study include the valid and reproducible methods used to assess ST, PA, and PF. We also used cNeuro for MRI analysis, which provides a reliable, highly standardized, and normalized multi-region volumetric assessment of a large array of brain structures that has proven utility in clinical brain research. However, our relatively small sample size reduced the statistical power and could have led to incorrect acceptance of the null hypothesis. However, the sample size is comparable to other MRI studies (N = 49 and N = 40) in adolescents (Ruotsalainen et al., 2019; Jalanko et al., 2024). Moreover, as the primary selection criterion was based on the 50-m shuttle run test results, the sample may not be fully representative. However, the voluntary participants in the FitBrain study did not differ in total PA, ST, parental education, weight or height at the 8-year follow-up examinations of the PANIC study from other adolescents. Our cross-sectional study design did not allow us to establish causal associations of ST and PA with the brains of adolescents. Furthermore, self-reports may lead to errors due to social desirability and recall bias (Choi and Pak, 2005). Moreover, the used questionnaire did not assess the content played on screens or the intensity of PA; therefore, screen time apps and accelerometers or smart devices could provide more comprehensive information on the content and amount of ST and the intensity of PA, which was not available at the time of the study. Moreover, our data did not allow us to study the associations of the use of different types of smart devices because of our lack of data on application usage. The data were collected during the years 2018–2019, a period when patterns of ST usage may have differed from those observed today. We did not adjust the analyses for socioeconomic status, as the FitBrain participants had a higher parental educational background than the original participants of the PANIC study (Skog et al., 2024, 2025). In addition, parental educational background showed limited variability within the FitBrain sample, with most participants belonging to the two highest educational categories and only a few participants represented in the lowest category. Consequently, our findings may not be generalizable to populations with lower socioeconomic status. Furthermore, we did not control the analyses for all possible confounding factors. Data for sleep or dietary factors were not available in the Fitbrain study. Therefore, residual confounding by socioeconomic factors, sleep, dietary habits, and other unmeasured factors cannot be excluded.

## 6 Conclusion

We found that higher levels of self-reported total ST and longer time spent using smart devices were associated with lower medial temporal volumes but larger frontal lobe volumes. PA and neuromuscular fitness had weaker and less consistent associations with brain volumes. Higher self-reported total PA was associated with one gyrus in the occipital lobe. Better neuromuscular fitness was negatively associated with the volumes of the right hippocampus and posterior cingulate gyrus but positively associated with the volume of the parietal lobe. Moreover, total PA or neuromuscular fitness was not associated with frontal lobe volumes. In conclusion, ST and smart device use may have complex and context-dependent associations with brain structures in adolescence. Further large-scale long-term studies are needed to clarify the causal relationships between different types of ST, lifestyle habits, and their underlying mechanisms in relation to the adolescent brain structures.

## CRediT authorship contribution statement

**Hannamari Skog:** Writing – review & editing, Writing – original draft, Visualization, Project administration, Methodology, Investigation, Formal analysis, Data curation. **Sara Määttä:** Writing – review & editing, Supervision, Methodology, Data curation, Conceptualization. **Laura Säisänen:** Writing – review & editing, Supervision, Methodology, Formal analysis, Data curation. **Timo A. Lakka:** Writing – review & editing, Supervision, Methodology, Funding acquisition, Data curation, Conceptualization. **Markus Niiranen:** Writing – review & editing, Visualization. **Eero A. Haapala:** Writing – review & editing, Supervision, Methodology, Formal analysis, Data curation. **Mervi Könönen:** Writing – review & editing, Supervision, Methodology, Data curation. **Juhana Hakumäki:** Writing – review & editing, Supervision, Software, Resources, Methodology, Data curation.

## Declaration of generative AI and AI-assisted technologies in the manuscript preparation process

During the preparation of this work the author(s) used Copilot for text structuring and language editing. After using this tool, the authors reviewed and edited the content as needed and take full responsibility for the content of the published article.

## Declaration of competing interest

The authors declare the following financial interests/personal relationships which may be considered as potential competing interests: Hannamari Skog reports financial support was provided by Juho Vainio Foundation. Hannamari Skog reports financial support was provided by Jenny and Antti Wihuri Foundation. Hannamari Skog reports financial support was provided by Yrjö Jahnsson Foundation. Hannamari Skog reports financial support was provided by Orion Research Foundation. Timo Lakka reports financial support was provided by Juho Vainio Foundation. Timo Lakka reports financial support was provided by Paavo Nurmi Foundation. Timo Lakka reports financial support was provided by The Finnish Innovation Fund Sitra. Timo Lakka reports financial support was provided by Finnish Foundation for Cardiovascular Research. Timo Lakka reports financial support was provided by Yrjö Jahnsson Foundation. Timo Lakka reports financial support was provided by Diabetes Research Foundation in Finland. Timo Lakka reports financial support was provided by Finnish Cultural Foundation. Timo Lakka reports financial support was provided by The Social Insurance Institution of Finland. Timo Lakka reports financial support was provided by Research Committee of the Kuopio University Hospital Catchment Area. Timo Lakka reports financial support was provided by the Ministry of Social Affairs and Health of Finland. Timo Lakka reports financial support was provided by the Ministry of Education and Culture of Finland. Timo Lakka reports financial support was provided by Finnish Foundation for Cardiovascular Research. Timo Lakka reports financial support was provided by Research Council of Finland. If there are other authors, they declare that they have no known competing financial interests or personal relationships that could have appeared to influence the work reported in this paper.

## Data Availability

The data that has been used is confidential.
