# Supplementary material for: Associations of screen time, physical activity and neuromuscular fitness with brain structure in adolescents
Source: Neuroimage Rep. 2026 Nov 7;6(4):100431. doi: 10.1016/j.ynirp.2026.100431 (PMC13763068; doi:10.1016/j.ynirp.2026.100431)
Supplement: Supplementary file 1 [file mmc1.pdf]

Supplement table 1 – korrelaatiot / correlations (ilman TMS)

			Motor Fitness	Muscular strenght		Screen time				Physical activity			
Muuttuja (suomi)	Variable (English, cNeuro)		50-m shuttle run	Standing Long Jump	Box & block sum score	Total screen time	Smart device	Computer	TV&video	Total PA	Organized sports	All supervised exercise	Unsupervised PA
nAivokudostilavuus_YHTEENSA	mri__t1__cross__volume__brain_tissue_all_regions_total_normalized	Pearson Correlation	-.390**	.351*	-0.07033409020428368	0.003750493656339659	-0.07477963401531243	0.05123794855945313	0.1394769445433706	0.0009775219406729803	0.02005586999528494	0.1036319214014538	0.05735756776249935
		Sig. (2-tailed)	0.008919143261314075	0.01965720709553413	0.6461567882804587	0.9807235269568182	0.6295016056241992	0.7411681850243196	0.3665388568165932	0.9949753754960526	0.8971846683573121	0.5032194290168857	0.7148601485349756
		N	44	44	45	44	44	44	44	44	44	44	43
nKolmas._aivokammiotilavuus_YHTEENSA	mri__t1__cross__volume__3rd_ventricle_total_normalized	Pearson Correlation	.420**	-.517**	0.1104028672341449	-0.03977303701899166	0.0993305371545124	-0.2061932991839211	0.06028315222272305	0.07707970963538506	0.06714470443917217	0.008069554118840945	0.08988781067355692
		Sig. (2-tailed)	0.004565066278952209	0.0003223028294436976	0.4703093960006622	0.7976954279727277	0.5211942685125036	0.1793242353391772	0.697486712139245	0.6189699372772357	0.6649748761249438	0.958538892651296	0.5664907793565246
		N	44	44	45	44	44	44	44	44	44	44	43
nNeljäs._aivokammiotilavuus_YHTEENSA	mri__t1__cross__volume__4th_ventricle_total_normalized	Pearson Correlation	0.2161376849674378	-0.1930797123510757	-0.02285512920085051	-0.17185841396811	-0.145917696169501	-0.1767789569832818	0.1446083402243363	0.1746708243356958	0.05201999527126628	-0.1240416845890201	0.1335859183962574
		Sig. (2-tailed)	0.1587935451340668	0.2092134878630761	0.88153637671882	0.2646422739774522	0.344599808849973	0.2509975453470016	0.3489931203818895	0.2567843185458791	0.7373572761136484	0.4224270836877966	0.393101758926378
		N	44	44	45	44	44	44	44	44	44	44	43
nAccumbensAreatilavuus_DX	mri__t1__cross__volume__accumbens_area_right_normalized	Pearson Correlation	-0.215364425483567	0.1602870425126989	-0.2740727031789075	0.1130656508247864	0.02325918183668399	-0.01122592956494807	0.1193231290084859	0.2099448395271429	-0.2152330670955819	-0.0813224212467993	.363*
		Sig. (2-tailed)	0.1603256111250826	0.2986399113750702	0.06847630479599741	0.4649319630390281	0.880873062202848	0.9423449415484619	0.4404245648301306	0.1713667002643396	0.1605869389255146	0.5997409292220632	0.01668945479970209
		N	44	44	45	44	44	44	44	44	44	44	43
nAccumbensAreatilavuus_SIN	mri__t1__cross__volume__accumbens_area_left_normalized	Pearson Correlation	-0.04639222279072577	0.09254166156438216	-.299*	0.06814558965518126	-0.02612353327572997	0.01499387880435551	-0.01887813369635856	0.05938105193240457	-0.1718529291336162	-0.05130597323817541	0.2258677705701982
		Sig. (2-tailed)	0.7649147497480527	0.5501972832856067	0.04628482587540726	0.6602810515108744	0.8663284120073299	0.9230438563192024	0.9031927340210001	0.70180273702287	0.2646577531077435	0.7408364565835073	0.1452914896309463
		N	44	44	45	44	44	44	44	44	44	44	43
nMantelitumaketilavuus_DX	mri__t1__cross__volume__amygdala_right_normalized	Pearson Correlation	0.1951335183379014	-0.0990896177770514	-0.06333245462244168	-0.063762349120445	-0.158184743875808	-0.0106348424601958	0.07537509212114595	-0.01579228931918757	-0.1611737805907904	-0.02540932350825481	0.0006836467269052857
		Sig. (2-tailed)	0.2043162101760602	0.5222103673981762	0.6793816176650249	0.6809300088125606	0.30510528885833	0.9453759929642453	0.6267680155091172	0.9189592771764037	0.2959394857474615	0.869951148415573	0.9965285210621794
		N	44	44	45	44	44	44	44	44	44	44	43
nMantelitumaketilavuus_SIN	mri__t1__cross__volume__amygdala_left_normalized	Pearson Correlation	-0.01892024488221696	-0.0472069889527777	0.01842089434668937	-0.1449227966378051	-0.2627763324771287	0.0531597082600181	-0.06043555219072702	-0.06677210228421272	-0.1375513202903558	-0.06771615367554028	-0.007754698298495565
		Sig. (2-tailed)	0.9029778152442492	0.7609063808726716	0.9044008411104156	0.3479349026829091	0.08483212967187294	0.7318146097399286	0.6967584930473266	0.6667255087767772	0.3732575123419235	0.6622934013372586	0.9606378068889289
		N	44	44	45	44	44	44	44	44	44	44	43
nAivorunkotilavuus_YHTEENSA	mri__t1__cross__volume__brain_stem_total_normalized	Pearson Correlation	-0.2893467049259578	0.2189734779479822	0.07438654066394287	-0.09105591445320338	-0.03167168948267225	-0.1944054508273505	.322*	0.1002203817936559	-0.08449186552956252	0.05924108172753533	0.1468464780683136
		Sig. (2-tailed)	0.05677643399341877	0.1532663389061265	0.6272281826650513	0.556645169546093	0.8382837260725866	0.2060429966839669	0.03299889446594502	0.5174497917236769	0.5855487798177558	0.7024732486834878	0.3473872165406617
		N	44	44	45	44	44	44	44	44	44	44	43
nCaudatetilavuus_DX	mri__t1__cross__volume__caudate_right_normalized	Pearson Correlation	-.351*	.408**	-.412**	-0.03531401304137092	-0.2516614359136972	0.09820400309272788	-0.01972196763191096	0.2041298691060366	0.03213707757964422	0.02556576659768176	0.219712712827619
		Sig. (2-tailed)	0.01939394807609624	0.005952246323445019	0.004870650620162955	0.8199774446126891	0.09936937976590421	0.5259539403121344	0.898887464778928	0.1838122508639198	0.8359397714230674	0.8691573834733498	0.1568739694176635
		N	44	44	45	44	44	44	44	44	44	44	43
nCaudatetilavuus_SIN	mri__t1__cross__volume__caudate_left_normalized	Pearson Correlation	-.391**	.478**	-.315*	-0.1190362275105966	-0.227363597879509	-0.1206964855503409	0.1127185846295053	0.2150154995618957	-0.07666745887038043	-0.06316268325988503	0.2530607122926588
		Sig. (2-tailed)	0.008593773110293402	0.001033768980383277	0.03480508729752017	0.4415323498426185	0.1377402036571915	0.4351430840755968	0.466312290810738	0.1610204556292861	0.6208521091969393	0.6837733959268384	0.1015667673206225
		N	44	44	45	44	44	44	44	44	44	44	43
nPikkuaivojenHarmaaAinetilavuus_DX	mri__t1__cross__volume__cerebellum_exterior_right_normalized	Pearson Correlation	-0.1275185940255699	0.05004664560868854	-0.03785363505112841	-0.2634939070628768	-0.1049159434137093	-.351*	.359*	0.1033903357445812	-0.1361505419633622	0.05364251415416679	0.2291064402977219
		Sig. (2-tailed)	0.4094357179252253	0.7469851485286371	0.8050067516790906	0.08395391434524552	0.497915565947823	0.01930182496790717	0.0166477112447749	0.504220542676126	0.3781907886955654	0.729470664148735	0.1394563545603068
		N	44	44	45	44	44	44	44	44	44	44	43
nPikkuaivojenHarmaaAinetilavuus_SIN	mri__t1__cross__volume__cerebellum_exterior_left_normalized	Pearson Correlation	-0.03761012177236976	-0.01350858270313346	-0.05752714568614665	-0.2155054948762406	-0.1433917610556606	-0.2412528308680474	.342*	0.1159984526164874	-0.1784226129631081	0.07168186587083042	0.2416889471406923
		Sig. (2-tailed)	0.8084849775804736	0.930647592820641	0.7073960682066316	0.1600453091252768	0.3531057128897082	0.1146520850233745	0.02294779594278186	0.4533557206174252	0.2465470665053853	0.6438010012706399	0.1184274680435511
		N	44	44	45	44	44	44	44	44	44	44	43
nPikkuaivojenValkeaAinetilavuus_DX	mri__t1__cross__volume__cerebellum_white_matter_right_normalized	Pearson Correlation	-0.1161168855283629	0.1232487927193706	-0.209262226365706	-0.0591623312386184 1	-0.0407513431194701	-0.0483603190758119	0.2410370421023513	0.05280058568555544	-0.08899530507537745	0.02110331777931237	0.1740974054437017
		Sig. (2-tailed)	0.4528915649757101	0.4254219467419178	0.1677095561974712	0.7028505922716111	0.7928272960175221	0.7552429755365284	0.1149867694209664	0.7335596595077446	0.5656461710162348	0.891846011090669	0.2641887170947388
		N	44	44	45	44	44	44	44	44	44	44	43
nPikkuaivojenValkeaAinetilavuus_SIN	mri__t1__cross__volume__cerebellum_white_matter_left_normalized	Pearson Correlation	-0.1097287474347537	0.1435373502360191	-0.2314333204468035	-0.03150688812379718	-0.09173195581047323	0.03083215064799953	0.2131814888186279	-0.05931808678546124	-0.106582843274462	-0.05610543890938535	0.1172169022867976
		Sig. (2-tailed)	0.4782935717304639	0.3526120052007593	0.12609947317305	0.8391140906293111	0.55370686875712	0.8425155994154745	0.1647087423384547	0.7021043368886926	0.4910731845336602	0.717551848354065	0.4541108528074754
		N	44	44	45	44	44	44	44	44	44	44	43
nIsoaivojenValkeaAinetilavuus_DX	mri__t1__cross__volume__cerebral_white_matter_right_normalized	Pearson Correlation	0.05411952324698614	-0.1388949476161479	0.04541204732767858	0.1028993506726388	0.02329796182358141	0.1942788665264565	-0.1158337332799365	-0.1803395494403989	-0.1444549512017118	-0.1788010725317481	0.01601248374201236
		Sig. (2-tailed)	0.7271572513346987	0.3685617820907643	0.7670669044117001	0.5062582650959314	0.8806758746794514	0.2063442610299651	0.4540017093714939	0.241424330811174	0.3495100235787418	0.2455299185467174	0.9188255322766
		N	44	44	45	44	44	44	44	44	44	44	43

			Motor Fitness	Muscular strenght		Screen time				Physical activity			
nIsoaivojenValkeaAinetilavuus_SIN	mri__t1__cross__volume__cerebral_white_matter_left_normalized	Pearson Correlation	0.02039762496099424	-0.1335815757566203	0.04364706874179612	0.08317407815097683	-0.07928628867482823	0.2453326271453602	-0.1073771425401425	-0.2053681803419637	-0.1670063734678784	-0.20706508590107	-0.01771186648778718
		Sig. (2-tailed)	0.8954422986767695	0.3873381530794278	0.7758817692973685	0.5914313161358201	0.6089367135594814	0.1084624909537614	0.4878299154803349	0.1811093736213308	0.2785709107477752	0.1774518665134185	0.9102439964985815
		N	44	44	45	44	44	44	44	44	44	44	43
nCSFnmtilavuus_YHTEENSA	mri__t1__cross__volume__csfnm_total_normalized	Pearson Correlation	0.2182831470869127	-.337*	0.1274674089938772	0.2231329974858633	0.1849422194531994	0.08520866854381204	0.1415150917133801	-0.1278734893855912	-0.1875117412583901	-0.1131595857191246	0.008073613356498123
		Sig. (2-tailed)	0.1545986823098091	0.02523046655369176	0.4040404708719623	0.1454160960782745	0.2294207554196231	0.5823600438017916	0.3595073563240931	0.4081226773819393	0.2229006341774882	0.4645587485883671	0.9590203652384024
		N	44	44	45	44	44	44	44	44	44	44	43
nHippokampustilavuus_DX	mri__t1__cross__volume__hippocampus_right_normalized	Pearson Correlation	0.2438400024466554	-.355*	0.02436169009355054	-0.005561499034828506	-0.03473902021259368	-0.009774070051283761	0.02186867183563063	-0.04570939684626905	-0.2352240264551509	0.03280265304191272	0.09175251325627333
		Sig. (2-tailed)	0.1106967334699663	0.01789665298800474	0.8737887080977366	0.971418686408894	0.8228613378512555	0.9497913227923266	0.8879481075568799	0.7682787036947824	0.1242840235609697	0.8325900047078595	0.5584342210591838
		N	44	44	45	44	44	44	44	44	44	44	43
nHippokampustilavuus_SIN	mri__t1__cross__volume__hippocampus_left_normalized	Pearson Correlation	0.06161282113970153	-0.2308419306178181	0.04476610848127464	-0.04718492340674916	-0.09717570551216216	-0.04032711241393826	0.1463214717953367	0.1129018069856901	-0.2140529295610062	0.08687332770075651	0.1870264021723399
		Sig. (2-tailed)	0.6911422165254353	0.1316580851503571	0.7702896212394981	0.761014854835526	0.5303171692881864	0.7949373591824447	0.343251902645201	0.4655833184188305	0.1629486610115985	0.5749850367664563	0.2297874153960802
		N	44	44	45	44	44	44	44	44	44	44	43
nTemporaalisarvi_sivuaivokammiotilavuus_DX	mri__t1__cross__volume__inferior_lateral_ventricle_right_normalized	Pearson Correlation	-0.1941291529148561	0.184863204167577	0.2926534962654957	0.02718307722725637	-0.2192947113488628	0.2094181757225485	-0.145861526101551	-0.1299411107113279	0.0604470391525594	-0.0790949134747128	-0.1545544499756984
		Sig. (2-tailed)	0.2067009697288618	0.229623306150175	0.05107101030723828	0.8609590162825219	0.1526492334459779	0.1724681817604417	0.3447875764009942	0.4005211151765794	0.696703615324411	0.6098041216331025	0.3223797247133238
		N	44	44	45	44	44	44	44	44	44	44	43
nTemporaalisarvi_sivuaivokammiotilavuus_SIN	mri__t1__cross__volume__inferior_lateral_ventricle_left_normalized	Pearson Correlation	-0.1572351425143652	-0.01029050623885427	.312*	-0.1116987305475809	-0.07730795463690444	-0.1185778352012416	-0.2692520847508817	0.07617813858242675	0.2329744604249664	-0.04260381942103286	-0.07940755565691354
		Sig. (2-tailed)	0.3080547991570417	0.9471420780103401	0.03719298185690154	0.4703810412069112	0.6179288957750542	0.4433054828777661	0.07716104815702701	0.6230892575144064	0.128029740582578	0.7836306491249257	0.612739509620288
		N	44	44	45	44	44	44	44	44	44	44	43
nSivuaivokammiotilavuus_DX	mri__t1__cross__volume__lateral_ventricle_right_normalized	Pearson Correlation	-0.02211419505634184	-0.046063577862626	0.1712824032743594	-0.2163527980691875	-0.04458457926654678	-.340*	0.02997007359052112	.319*	.448**	0.2126190971915624	0.07284745410059526
		Sig. (2-tailed)	0.886698220759046	0.7665332954090168	0.2605879556857571	0.1583692445277389	0.7738292921377725	0.02408678619734455	0.8468656467358237	0.03467612390803539	0.002286567050260976	0.1658519258122288	0.642480006799495
		N	44	44	45	44	44	44	44	44	44	44	43
nSivuaivokammiotilavuus_SIN	mri__t1__cross__volume__lateral_ventricle_left_normalized	Pearson Correlation	0.172711278619382	-0.1641827095426732	0.1413944460236099	-0.09866740159285842	0.04668665967738427	-0.1943158297519894	-0.1151495745000448	0.2059615990068693	.426**	0.07329378702303993	-0.04903656841216121
		Sig. (2-tailed)	0.2622426609340695	0.2868940301012262	0.3541976553892716	0.5239934653596747	0.7634655151881236	0.2062562585424597	0.4566901584273833	0.179824238744795	0.00396494183012281	0.636344039058433	0.7548376677739488
		N	44	44	45	44	44	44	44	44	44	44	43
nPallidumtilavuus_DX	mri__t1__cross__volume__pallidum_right_normalized	Pearson Correlation	-0.2203116403934114	0.06941491528979787	-0.08144699236179032	0.04407164764091157	-0.01715758813569364	-0.001263476855558556	0.07905094013462796	-0.1448016302929996	-0.2487835023646389	0.0001365313042949492	0.01646889474529827
		Sig. (2-tailed)	0.1507076770741523	0.6543468277967808	0.5948137508090197	0.7763641567037838	0.9119793905713343	0.9935055476681773	0.6100035055197032	0.3483424220445261	0.1034288081971531	0.9992982021236705	0.9165198149376726
		N	44	44	45	44	44	44	44	44	44	44	43
nPallidumtilavuus_SIN	mri__t1__cross__volume__pallidum_left_normalized	Pearson Correlation	-.303*	0.1971230730789977	-0.08198153676607778	0.01867068878522455	-0.1012852885827212	0.02395712864728572	0.1224604997647361	-0.1974928167524694	-.303*	0.01353098743846537	0.0008952392259747944
		Sig. (2-tailed)	0.04520966779975877	0.1996493215871576	0.5923900094503014	0.9042515500082358	0.5129863614620789	0.8773252595054702	0.4284112465340782	0.1987903530687959	0.04569845651582544	0.9305328483173625	0.995454088718145
		N	44	44	45	44	44	44	44	44	44	44	43
nPutamentilavuus_DX	mri__t1__cross__volume__putamen_right_normalized	Pearson Correlation	-0.05137994912431333	0.0745757936710419	0.007072899932009157	.358*	0.06689555289574724	.332*	0.03840381089389278	-0.1082932535217884	-0.1652320459987115	-0.02122996159479773	0.05148839022395496
		Sig. (2-tailed)	0.7404757592481024	0.630438510035573	0.9632211098492139	0.01717708371772211	0.6661452945104482	0.02759500607701362	0.804521524091639	0.4841031461952159	0.2837822842837694	0.891200845112367	0.742986875986535
		N	44	44	45	44	44	44	44	44	44	44	43
nPutamentilavuus_SIN	mri__t1__cross__volume__putamen_left_normalized	Pearson Correlation	-0.09533688205890983	0.06753884381513647	-0.0403255236958969	.338*	0.04643967684711883	.314*	0.05034381733413407	-0.1195855045405227	-0.1905522188532469	-0.05577126250605934	0.08725603241509722
		Sig. (2-tailed)	0.5381634893172808	0.6631249676457998	0.7925455484083781	0.02498834152051008	0.7646811244941946	0.03787297473436919	0.7455327859673749	0.4394128257435135	0.2153519756073992	0.7191652182626612	0.5779519979282878
		N	44	44	45	44	44	44	44	44	44	44	43
nTalamustilavuus_DX	mri__t1__cross__volume__thalamus_proper_right_normalized	Pearson Correlation	-0.1989774478918186	0.08264662149090941	0.01816539417766417	0.04157346543194026	0.1645218796319801	-0.01651402310896777	0.02044746803492596	0.1811716149810205	0.1216785528282906	0.1194175060308936	0.0980518329360206
		Sig. (2-tailed)	0.1953675589891758	0.5937931667740175	0.9057208014724094	0.7887423485246339	0.2858858224649278	0.9152687375505355	0.8951882240998351	0.2392233943061336	0.4313880817262209	0.4400604920113268	0.5316215311353226
		N	44	44	45	44	44	44	44	44	44	44	43
nTalamustilavuus_SIN	mri__t1__cross__volume__thalamus_proper_left_normalized	Pearson Correlation	-.340*	0.1911019866285903	0.01705268155200487	-0.06598378451654346	0.0312539906113635	-0.09601065036214454	0.1349339686810253	0.08104183763894875	0.02893596197141469	0.1307451354536115	0.05089877057636561
		Sig. (2-tailed)	0.02394243918073823	0.2140062391715838	0.9114721619815107	0.6704350990409225	0.8403886700148565	0.5352819928900537	0.3825065677870492	0.6010045244013806	0.8520897099726354	0.3975874283250997	0.7458315465050684
		N	44	44	45	44	44	44	44	44	44	44	43
nVentral_diencephalontilavuus_DX	mri__t1__cross__volume__ventral_diencephalon_right_normalized	Pearson Correlation	-0.1079266095717712	-0.02045083542287425	0.1160532041530957	-0.1626650552645744	-0.02609522295760154	-0.2470515905325867	0.1548695173548162	-0.05193596473762928	-0.2228558525885598	0.003776644589302914	0.07527022533633042
		Sig. (2-tailed)	0.4855928771354761	0.8951710592464293	0.4477497705740088	0.2914336712711665	0.8664719614222636	0.1059322415903526	0.3154813270833172	0.7377664599461209	0.1459297363331752	0.9805891440534916	0.6314286207138791
		N	44	44	45	44	44	44	44	44	44	44	43
nVentral_diencephalontilavuus_SIN	mri__t1__cross__volume__ventral_diencephalon_left_normalized	Pearson Correlation	-0.1570904755262175	-0.02020421133746267	0.1601336747996841	-0.1624586992513303	0.02104592339272826	-0.233933763174261	0.09990948588293283	-0.111570422766414	-0.2055550448134069	-0.03447913525351989	0.01656058551268052
		Sig. (2-tailed)	0.3085057326206235	0.8964283203688488	0.29335938597378	0.2920545018387893	0.8921384200506094	0.1264222090459091	0.518756518360263	0.4708942649302406	0.1807039845735174	0.8241655578961358	0.9160566884020768

			Motor Fitness	Muscular strenght		Screen time				Physical activity			
		N	44	44	45	44	44	44	44	44	44	44	43
nVesseltilavuus_DX	mri__t1__cross__volume__vessel_right_normalized	Pearson Correlation	-0.103890621898271	0.1749228594541324	-0.08434611894224424	0.03045356208143541	-0.07465492345171995	0.2177113771598555	-.345*	-0.09364793097621762	0.1602549364111961	-0.2289344005909336	-0.1837196243896295
		Sig. (2-tailed)	0.5021485158296927	0.2560878300164707	0.5817213067127087	0.8444253977485539	0.6300747404094216	0.155708605970158	0.02188558726333761	0.5454194104618438	0.2987379820706916	0.1349681976994018	0.2382784638526745
		N	44	44	45	44	44	44	44	44	44	44	43
nVesseltilavuus_SIN	mri__t1__cross__volume__vessel_left_normalized	Pearson Correlation	0.005548308760646481	0.0944547395247608	-.310*	-0.1571102081698391	-0.189025299547216	0.07772397929631057	-0.1874529350443931	0.1607842774983694	0.2458689623265691	0.1848559719125501	0.05944618337244551
		Sig. (2-tailed)	0.9714864452022266	0.5419474414810447	0.03835193986176398	0.3084442003234386	0.2191203947497694	0.6160332801239089	0.2230484096871939	0.2971237117603173	0.1076681289794472	0.2296418517050995	0.704938003867278
		N	44	44	45	44	44	44	44	44	44	44	43
nCerebellar_vermal_lobules_I_Vtilavuus_YHTEENSA	mri__t1__cross__volume__cerebellar_vermal_lobules_i_v_total_normalized	Pearson Correlation	0.0704300280314217	-0.05882611447211764	0.09863977389228155	0.2174513683508506	.312*	-0.04853089441941608	0.1191878960615597	0.07380115011815684	-0.1309642441200068	0.08359449139395653	0.2105676491148445
		Sig. (2-tailed)	0.6496161567878493	0.7044624131340811	0.5191536813196844	0.1562152600047411	0.03952970538056898	0.7544064358316548	0.4409465352402124	0.6340041941054837	0.3967901255065979	0.5895517709688284	0.1753063060980722
		N	44	44	45	44	44	44	44	44	44	44	43
nCerebellar_vermal_lobules_VI_VIItilavuus_YHTEENSA	mri__t1__cross__volume__cerebellar_vermal_lobules_vi_vii_total_normalized	Pearson Correlation	0.01288351911345796	-0.1632318682093391	0.1266658169625261	-0.1184474498034022	0.03696386468413166	-.389**	.304*	-0.04738540143762076	-0.1866084452730964	0.1267580528190518	0.005364340147298155
		Sig. (2-tailed)	0.9338493701824528	0.2897327939844067	0.4070271041023087	0.4438105493261875	0.8117157050909596	0.009150097581228749	0.04451339494888865	0.7600294737581562	0.2251780018096592	0.4122577068205677	0.9727655131880715
		N	44	44	45	44	44	44	44	44	44	44	43
nCerebellar_vermal_lobules_VIII_Xtilavuus_YHTEENSA	mri__t1__cross__volume__cerebellar_vermal_lobules_viii_x_total_normalized	Pearson Correlation	-0.2613253695583321	0.0894260443424699	0.1287623776131052	-0.1911736913825444	-0.1732456471070824	0.05951911584634489	-0.115443780955256	-0.02648629225736232	0.08679415811618713	0.07916335318691688	-0.1266550499292424
		Sig. (2-tailed)	0.08662977881315621	0.5637590895354434	0.3992424018866876	0.2138311502667319	0.2607465703402354	0.7011415763576764	0.4555329985564798	0.8644893936845819	0.5753348188837948	0.6094938578705542	0.4183275245066317
		N	44	44	45	44	44	44	44	44	44	44	43
nAnterior_cingulate_gyrustilavuus_DX	mri__t1__cross__volume__anterior_cingulate_gyrus_right_normalized	Pearson Correlation	-0.1138436556886297	0.05051645950797216	0.1201059548042256	-0.0675469147127771	-0.07267965647782215	0.1108763626157091	-0.1098946045947045	0.08832241894159786	0.2269976929660485	-0.1431989483074535	-0.01741286528767473
		Sig. (2-tailed)	0.4618457085319989	0.7446894343950743	0.4319413954760472	0.663087107205418	0.639180954518743	0.4736756210817941	0.4776247078522526	0.5685999429025856	0.1383919490342129	0.3537602086201315	0.9117531885540795
		N	44	44	45	44	44	44	44	44	44	44	43
nAnterior_cingulate_gyrustilavuus_SIN	mri__t1__cross__volume__anterior_cingulate_gyrus_left_normalized	Pearson Correlation	-0.05293978586683831	0.05966120689638885	0.02986999270359788	-0.08912066986706466	-0.02471185494336312	0.04957142579149831	-0.1414390310426538	0.09693283542384983	-0.0941011904421564	0.08401267790057752	0.1341390678810971
		Sig. (2-tailed)	0.7328830994319693	0.7004613582339576	0.8455661142516109	0.565096642574405	0.8734915083647588	0.749309481336637	0.3597682850259762	0.5313502852736502	0.543467573387895	0.5876848187038514	0.3911275294122261
		N	44	44	45	44	44	44	44	44	44	44	43
nAnterior_insulatilavuus_DX	mri__t1__cross__volume__anterior_insula_right_normalized	Pearson Correlation	-0.1811471668072951	0.1995658743734842	0.1674832916270749	-0.01274693057252352	0.06104440156545957	-0.2443700169039529	0.2403573286857796	0.1285449700817476	-0.246414051998754	0.1496051645405341	0.1144771074743451
		Sig. (2-tailed)	0.2392878681977728	0.1940225424268527	0.271468844643743	0.9345491598346527	0.6938518948688543	0.1098994036553603	0.1160458336907964	0.4056449610844979	0.1068653573285448	0.3324109548895859	0.4648007847463811
		N	44	44	45	44	44	44	44	44	44	44	43
nAnterior_insulatilavuus_SIN	mri__t1__cross__volume__anterior_insula_left_normalized	Pearson Correlation	-.342*	0.2936168553120137	0.1656382617148578	0.005810825057155755	-0.01788980940993818	-0.2068628673669434	.404**	0.05842303487290799	-0.2332079853801529	0.1760084703739969	0.1355049407987281
		Sig. (2-tailed)	0.02308025127185252	0.05305897976899943	0.2768598531152426	0.9701379298531214	0.9082386796207232	0.1778849237668771	0.006520803707346273	0.7063964606711085	0.1276370129091146	0.2531022541386158	0.3862775303825746
		N	44	44	45	44	44	44	44	44	44	44	43
nAnterior_orbital_gyrustilavuus_DX	mri__t1__cross__volume__anterior_orbital_gyrus_right_normalized	Pearson Correlation	-0.05178145605392404	-0.06190631390843864	0.1046077015822236	-0.2054516176875045	-0.2396617887041705	-0.1964818676316882	0.2634069179133183	0.2751323159802587	0.04986905803374303	0.06190291493470024	0.1591338962400608
		Sig. (2-tailed)	0.7385190216036448	0.6897446137060435	0.4940617485225053	0.1809282819711882	0.1171371878686903	0.2011451233913179	0.08405999808067038	0.07067741994339978	0.747853483302571	0.6897607936803487	0.3080723847800739
		N	44	44	45	44	44	44	44	44	44	44	43
nAnterior_orbital_gyrustilavuus_SIN	mri__t1__cross__volume__anterior_orbital_gyrus_left_normalized	Pearson Correlation	0.161857820922453	-0.1426077265978782	0.06440127596187872	-0.03709002224919077	-0.01148903578817449	0.01588664032880185	0.02448934092745017	0.0750995076036472	0.1512992517907815	.348*	-0.03787541827446046
		Sig. (2-tailed)	0.2938671425396835	0.3557716912304465	0.6742687517306687	0.8110847816344529	0.9409960083316222	0.9184767220369673	0.8746215098171182	0.6280325397395582	0.3269023283286856	0.02052920075388793	0.8094516024605843
		N	44	44	45	44	44	44	44	44	44	44	43
nAngular_gyrustilavuus_DX	mri__t1__cross__volume__angular_gyrus_right_normalized	Pearson Correlation	-0.01442625290436281	0.05941521007435425	-0.1111912497178712	-0.09799887061764952	-0.0355422008368743	-0.1612712151620704	-0.01635426604964225	0.01756484819837309	0.2846100216994491	0.1487317211900214	-0.2442731725516233
		Sig. (2-tailed)	0.9259489632875884	0.7016391401875123	0.46712581198958	0.5268229354814795	0.8188336098460213	0.2956437272472739	0.9160854961063191	0.9098985771303602	0.06114135056912302	0.3352735514627105	0.1144219321483493
		N	44	44	45	44	44	44	44	44	44	44	43
nAngular_gyrustilavuus_SIN	mri__t1__cross__volume__angular_gyrus_left_normalized	Pearson Correlation	-0.1522033485754285	0.1006041534085476	0.103937975846991	-0.2722304719256912	-0.2853825405956739	-0.04631127281640138	-0.1092404210560452	-0.04052853664302077	0.09665789687140945	-0.1921143365610037	-0.07765570546843623
		Sig. (2-tailed)	0.3239860229244629	0.5158390288822817	0.4968460424891437	0.07382101864839377	0.06041173597242912	0.7653133292868872	0.480265741240959	0.7939353224617366	0.5325209991085574	0.2115434986600917	0.620624281094994
		N	44	44	45	44	44	44	44	44	44	44	43
nCalcarine_cortextilavuus_DX	mri__t1__cross__volume__calcarine_cortex_right_normalized	Pearson Correlation	-0.2876508619650346	0.1279069133263595	0.2280989519315546	-0.06450073338041833	-0.1680590040785716	0.2646375409701144	-0.09725022998945777	-0.1370058864258247	-0.006580616311288741	-0.1623218904893313	-0.1655088962143662
		Sig. (2-tailed)	0.05830951201376539	0.407999140307445	0.1318050388051664	0.6774348694726036	0.2755090834141038	0.0825689375845497	0.5300003557873764	0.3751738439282991	0.9661840908830875	0.2924665683282575	0.2888398903927877
		N	44	44	45	44	44	44	44	44	44	44	43
nCalcarine_cortextilavuus_SIN	mri__t1__cross__volume__calcarine_cortex_left_normalized	Pearson Correlation	-0.2357590734618794	0.1001245347534595	0.1714123966167107	0.02739364148223323	-0.08529020717405256	.319*	-0.06031243644301219	-0.1743921680701027	-0.02417193169827582	-0.1254118943031194	-0.1755564421094805
		Sig. (2-tailed)	0.1234053785209007	0.5178524712744802	0.2602208728532454	0.8598926802500022	0.5819978105083201	0.03452032609246001	0.6973467612657919	0.257555847409042	0.8762338472961051	0.4172797790622214	0.2601441698577477
		N	44	44	45	44	44	44	44	44	44	44	43

			Motor Fitness	Muscular strenght		Screen time				Physical activity			
nCentral_operculumtilavuus_DX	mri__t1__cross__volume__central_operculum_right_normalized	Pearson Correlation	-0.1201807712446758	0.1747274289810445	-0.2581636905421437	0.1408321983141275	0.107717443962786	0.2460764523394941	-0.2095519475320923	-0.2215757392939913	-0.1297677519720767	-0.09150710964236972	-0.09567722348765262
		Sig. (2-tailed)	0.4371222097752899	0.2566277837288802	0.08685220459463458	0.3618541484960364	0.4864438191794618	0.1073620108764757	0.1721879248849733	0.1483195811975991	0.4011552968953349	0.5546833120645194	0.541654710858544
		N	44	44	45	44	44	44	44	44	44	44	43
nCentral_operculumtilavuus_SIN	mri__t1__cross__volume__central_operculum_left_normalized	Pearson Correlation	-0.1976613022909939	0.2675327758452168	-0.2525585627800659	0.1735004831479509	0.1152520133763997	0.118317930624139	0.01129272674320735	-0.2381920162926322	-0.2268012856380636	0.05228254042841625	-0.171447180867796
		Sig. (2-tailed)	0.1983998013126536	0.07914246070899009	0.09417071623830994	0.2600351044849592	0.4562870697947443	0.4443125737511665	0.9420024603168313	0.1194688906802272	0.1387427303881781	0.7360792840447126	0.271641586305373
		N	44	44	45	44	44	44	44	44	44	44	43
nCuneustilavuus_DX	mri__t1__cross__volume__cuneus_right_normalized	Pearson Correlation	0.1852911070105038	-0.1949569382668387	-0.1109191492207005	0.1848654667399148	-0.02854385884718138	.394**	-0.07620501983691055	-0.1894175974002522	0.002252856752095038	0.2446031763428826	-0.1662430583791832
		Sig. (2-tailed)	0.228527869012576	0.2047340770939774	0.4682232820323122	0.2296175044832966	0.8540721620713188	0.00812196719239107	0.6229662704259689	0.2181478814769884	0.988420261606275	0.1095500374722999	0.2866762040783755
		N	44	44	45	44	44	44	44	44	44	44	43
nCuneustilavuus_SIN	mri__t1__cross__volume__cuneus_left_normalized	Pearson Correlation	0.05733169439119343	0.001253066151523989	-0.09389270319268828	.400**	0.2004438280356806	.334*	0.1540772803437537	-0.1322795221791285	0.1286747694860025	0.02180449434130806	-0.1442632064066424
		Sig. (2-tailed)	0.7116420541760787	0.9935590590557645	0.5395557276092718	0.007217170645744076	0.1920279282484455	0.02653546891419008	0.3179935552351093	0.3920236081686567	0.4051670114112227	0.8882748607819678	0.3560270379294552
		N	44	44	45	44	44	44	44	44	44	44	43
nEntorinaalinen_aluetilavuus_DX	mri__t1__cross__volume__entorhinal_area_right_normalized	Pearson Correlation	0.1567979254168155	-0.08245747893679267	0.01503080741814368	-0.2043732231137819	-.313*	-0.0963640764179232	0.1174076752603446	0.02075307219446982	-0.03115863541188344	0.01403606240868507	-0.04439900114271513
		Sig. (2-tailed)	0.309418909277338	0.5946411231721945	0.921934031066387	0.1832788192905658	0.03848611428902338	0.5337735030897135	0.4478495784699508	0.893630637378993	0.8408693560458953	0.9279465109309483	0.7774037772973141
		N	44	44	45	44	44	44	44	44	44	44	43
nEntorinaalinen_aluetilavuus_SIN	mri__t1__cross__volume__entorhinal_area_left_normalized	Pearson Correlation	-0.1016730691419032	-0.007742149881806093	0.08794659720285287	-0.2136342383746957	-.337*	-0.00365422799486094	0.03220654622681492	-0.1409686030976572	0.07210476313314186	0.06280887112145597	-0.2742794170214556
		Sig. (2-tailed)	0.511365840772625	0.9602197125304285	0.5656450356051533	0.1637925932900838	0.02536105620842051	0.9812182158900511	0.8355900083710567	0.3613846497412406	0.6418412367134472	0.6854530708947336	0.0750955049889653
		N	44	44	45	44	44	44	44	44	44	44	43
nFrontal_operculumtilavuus_DX	mri__t1__cross__volume__frontal_operculum_right_normalized	Pearson Correlation	-0.2304332296791048	.308*	0.222835067357963	0.1996083158861001	0.176838060792479	0.07042453091784731	0.02945617965610802	-0.03790906025272318	-0.1415076073479897	0.03682052397511083	-0.09067216731252029
		Sig. (2-tailed)	0.13236214322921	0.04184843711448025	0.1412018642212952	0.1939257841642325	0.2508365813347792	0.6496417382491364	0.8494609091296352	0.8069916036602371	0.3595330266086738	0.8124327057508959	0.5630953845976144
		N	44	44	45	44	44	44	44	44	44	44	43
nFrontal_operculumtilavuus_SIN	mri__t1__cross__volume__frontal_operculum_left_normalized	Pearson Correlation	-0.2278458136923924	0.2823184283877023	-0.1083458740646578	0.1946003673940424	0.106430983476961	0.09478738317378976	0.2006894399701715	-0.03531038945707005	-0.2481791976041634	-0.139491680036458	0.1161312964786009
		Sig. (2-tailed)	0.1368847730639285	0.06334711905191108	0.478669924249523	0.2055797072531238	0.4916945244233754	0.5405190646443336	0.1914725355660664	0.8199956115029143	0.1042971140736804	0.3664877254511661	0.4583305405579716
		N	44	44	45	44	44	44	44	44	44	44	43
nFrontal_poletilavuus_DX	mri__t1__cross__volume__frontal_pole_right_normalized	Pearson Correlation	-0.1577837911064098	.306*	0.1106275812938985	0.2009978817654507	.312*	-0.1931064885799232	0.05924245240618813	0.0125000662099452	-0.03913511123149193	0.03956213060518535	-0.06939052059687095
		Sig. (2-tailed)	0.3063484584585011	0.04323194863395902	0.4694007975088901	0.1907766825707169	0.03924181821685778	0.2091491177103152	0.702466681517245	0.9358140534193196	0.8008739011237553	0.7987459168547255	0.6583810430057949
		N	44	44	45	44	44	44	44	44	44	44	43
nFrontal_poletilavuus_SIN	mri__t1__cross__volume__frontal_pole_left_normalized	Pearson Correlation	0.1491648985413402	-.311*	0.01808830784141532	0.05400613134574126	0.2199820947907765	-0.1235300533029501	-0.13499307766596	-0.04689549165598658	0.1761586933723708	-0.04866871097916376	-0.1247734894955672
		Sig. (2-tailed)	0.3338519620846159	0.03957236729138436	0.9061190930150439	0.7277069664709258	0.1513348597696406	0.4243582215258304	0.3822962093836306	0.7624381163685985	0.2526909688441795	0.7537307552579373	0.4253306466927163
		N	44	44	45	44	44	44	44	44	44	44	43
nFusiform_gyrustilavuus_DX	mri__t1__cross__volume__fusiform_gyrus_right_normalized	Pearson Correlation	-0.1718797304551351	0.1689146461040021	0.2395045445931007	-0.2355986222198106	-0.1520768326346055	-0.06123461315029708	-0.1633603089105847	0.2096259831244568	0.2434051774466379	0.09133884821133524	0.05357152104549762
		Sig. (2-tailed)	0.264582120957365	0.2730366128625502	0.1130585678945761	0.1236683767326452	0.324393133981319	0.6929447267619167	0.2893482696097642	0.1720329588070156	0.111354145448823	0.5554145543033585	0.7329638931582643
		N	44	44	45	44	44	44	44	44	44	44	43
nFusiform_gyrustilavuus_SIN	mri__t1__cross__volume__fusiform_gyrus_left_normalized	Pearson Correlation	-0.2970668892278681	0.2533120949068017	0.02827988181051598	-0.2308358784534906	-.305*	0.03103132604223015	-0.05704121347538161	0.1122813815150838	-0.05581777948593143	-0.1466628202270688	0.05705551899207302
		Sig. (2-tailed)	0.05020082682308313	0.09709699847632872	0.8536952759683349	0.1316684906050533	0.04411595539767882	0.8415112123195574	0.7130404994807742	0.4680542208188594	0.7189405665281708	0.3421149303602006	0.7162989503336236
		N	44	44	45	44	44	44	44	44	44	44	43
nGyrus_rectustilavuus_DX	mri__t1__cross__volume__gyrus_rectus_right_normalized	Pearson Correlation	-0.1392390539006028	0.2310550710435174	-0.1969749241862963	0.05275497533043857	0.08631862718020228	-0.1748416675017573	0.08709037801119594	-0.02448514583781843	-0.0516526679375719	0.1032737946548667	-0.1102097518719612
		Sig. (2-tailed)	0.3673649177065929	0.131292025492164	0.1946608158173768	0.7337813847115823	0.577437816417578	0.2563120623597597	0.5740265760517976	0.8746428162756632	0.7391464913156482	0.504703842371133	0.4817157642054513
		N	44	44	45	44	44	44	44	44	44	44	43
nGyrus_rectustilavuus_SIN	mri__t1__cross__volume__gyrus_rectus_left_normalized	Pearson Correlation	-0.110000038780214	0.2628031659515961	-.316*	-0.05372191135331852	0.01083620226470607	-.312*	.444**	0.1542789013800548	-0.1195819610379564	-0.06582156966721549	0.2139673299000565
		Sig. (2-tailed)	0.4771997724770269	0.0847991607276751	0.03443501525329737	0.7290854359376079	0.9443433468925749	0.03922586947967686	0.00252454110253585	0.3173530084818607	0.4394264811733646	0.6711993994001341	0.1682806874497139
		N	44	44	45	44	44	44	44	44	44	44	43
nInferior_occipital_gyrustilavuus_DX	mri__t1__cross__volume__inferior_occipital_gyrus_right_normalized	Pearson Correlation	-0.1540977042996959	0.08730493011146029	-0.1201959270669035	0.06031101386059059	0.208646029102241	-0.06827242967602762	-0.07770510731500543	.392**	0.2395554034410785	-0.03168130624346396	0.2160484331012959
		Sig. (2-tailed)	0.3179286315227234	0.5730798644605207	0.4315940233323934	0.6973535596348956	0.1740922855159331	0.659687127299433	0.6161192169758458	0.008431983488838288	0.1173047967023646	0.8382352766197871	0.1640816871225305
		N	44	44	45	44	44	44	44	44	44	44	43

			Motor Fitness	Muscular strenght		Screen time				Physical activity			
nInferior_occipital_gyrustilavuus_SIN	mri__t1__cross__volume__inferior_occipital_gyrus_left_normalized	Pearson Correlation	-0.02601707291512875	0.02761024192584649	-0.09467624366374543	-0.2017427475007528	-0.2178282252485307	0.06642029442836686	-0.09866148557479465	-0.1851200743915021	0.05572365447670485	0.08912143173402386	-0.087159429938568
		Sig. (2-tailed)	0.866868248605902	0.858796032742469	0.5361617208046208	0.1891036475728239	0.1554813072168918	0.6683800487290003	0.5240184711635674	0.228965283794254	0.7193951633889487	0.5650933037363212	0.578374685841374
		N	44	44	45	44	44	44	44	44	44	44	43
nInferior_temporal_gyrustilavuus_DX	mri__t1__cross__volume__inferior_temporal_gyrus_right_normalized	Pearson Correlation	0.09056856765578965	0.1009610515755121	0.03235006412804751	-0.18870057664936	-0.2683872102735779	-0.006535167561794455	0.10243846135158	-0.2132530136665992	-0.11723438900543	0.1256726414777076	-0.1982219573206101
		Sig. (2-tailed)	0.5587678739382855	0.5143433123918191	0.8329190854102322	0.2199276548482361	0.07815284392191027	0.9664175056071723	0.5081748732036131	0.1645637633949782	0.4485246643395793	0.4163043131947234	0.2025875882470015
		N	44	44	45	44	44	44	44	44	44	44	43
nInferior_temporal_gyrustilavuus_SIN	mri__t1__cross__volume__inferior_temporal_gyrus_left_normalized	Pearson Correlation	-0.1142933836156845	0.2666773028081539	0.2586301297086677	-0.1646210881862399	-0.0816525291315843	-0.02269938184900682	-0.1011436158110934	0.0208105013918322	.401**	0.1263016819454981	-0.1844852441895508
		Sig. (2-tailed)	0.4600667313491906	0.08014309264228704	0.08626373648374418	0.2855913545904181	0.5982557897488592	0.883720306645563	0.5135790491468405	0.8933379793028388	0.007031986979769198	0.4139563928804938	0.2362938173191645
		N	44	44	45	44	44	44	44	44	44	44	43
nLingual_gyrustilavuus_DX	mri__t1__cross__volume__lingual_gyrus_right_normalized	Pearson Correlation	-0.2712971805632962	0.2113474656056342	-0.04822569907991636	0.07254664735378023	0.04440647798776527	0.1714038277817983	-0.002028362198199588	-0.06003605757535492	-0.02029897290191192	-0.1521191354985645	-0.01402207717367535
		Sig. (2-tailed)	0.07485514310234094	0.1684579794471967	0.7530749760895672	0.6397960496577193	0.7747091916124864	0.2659272339290751	0.9895741022616351	0.6986679853617224	0.895945207026487	0.3242569734223564	0.9288881983932825
		N	44	44	45	44	44	44	44	44	44	44	43
nLingual_gyrustilavuus_SIN	mri__t1__cross__volume__lingual_gyrus_left_normalized	Pearson Correlation	-0.05208647145614902	0.01018716475242339	0.07228150661974424	-0.06061037603374596	0.05080864490565205	-0.1469534809449393	0.03908143941473022	0.1721287403688771	0.1168330135031167	0.0478798085382112	0.0523270615325652
		Sig. (2-tailed)	0.7370336234024429	0.9476721610863835	0.6370320494347201	0.695923456611935	0.7432627903305695	0.3411486223638811	0.8011414673496136	0.2638801123486201	0.4500904731989284	0.757600991694587	0.7389464572577307
		N	44	44	45	44	44	44	44	44	44	44	43
nLateral_orbital_gyrustilavuus_DX	mri__t1__cross__volume__lateral_orbital_gyrus_right_normalized	Pearson Correlation	0.03488274470813293	0.1013649403135456	-0.06915198698687286	0.004735142959218192	.315*	-0.2392353037538666	-0.114979683105274	0.1879856533068254	0.09225933453705389	0.1949112934264035	0.1263804360977922
		Sig. (2-tailed)	0.8221402657030651	0.5126532895285874	0.6517208668838014	0.975664064466404	0.03724587452858524	0.1178102045409013	0.457359092191026	0.2217121941619521	0.5514198006826961	0.2048421904213343	0.4193455365572192
		N	44	44	45	44	44	44	44	44	44	44	43
nLateral_orbital_gyrustilavuus_SIN	mri__t1__cross__volume__lateral_orbital_gyrus_left_normalized	Pearson Correlation	.314*	-0.1797560135962834	-0.1783180689617518	0.1429921709983495	0.1171981093875152	-0.078192348367341	0.0853755067453023	-0.009083433272315134	-0.2614840592138966	0.08387399044171265	0.1445967427124322
		Sig. (2-tailed)	0.03790175102025364	0.2429760396436131	0.2412153790420118	0.3544629270729074	0.4486660723633815	0.6139021076019533	0.5816189779502634	0.9533350232213333	0.08643174099259512	0.5883036829745989	0.3549042427993822
		N	44	44	45	44	44	44	44	44	44	44	43
nMiddle_cingulate_gyrustilavuus_DX	mri__t1__cross__volume__middle_cingulate_gyrus_right_normalized	Pearson Correlation	-0.04877447583545037	0.1224576870599504	-0.02032885849671495	0.03292120618402984	0.06340911733697586	-0.06251214132568518	0.002551647817429446	0.2075637200989365	0.1747631727633778	0.1097923836994574	0.1008906938472875
		Sig. (2-tailed)	0.7532123394687984	0.4284219336833353	0.8945522434602177	0.8319936455299388	0.6826043735166871	0.6868629117544379	0.9868845937926811	0.1763872602040704	0.2565289705616538	0.4780368829579971	0.5197470933160615
		N	44	44	45	44	44	44	44	44	44	44	43
nMiddle_cingulate_gyrustilavuus_SIN	mri__t1__cross__volume__middle_cingulate_gyrus_left_normalized	Pearson Correlation	-0.04978393518911101	0.03184768986737643	0.2077383574707247	-0.1152090440864512	-0.09468568241766967	-0.1101339769879869	0.1463392215311102	0.06049689567628066	-0.01060107007313073	0.06281464375766593	0.1394891744541546
		Sig. (2-tailed)	0.7482698106052619	0.8373971259772932	0.1708966707792821	0.4564561269708247	0.5409555766112857	0.4766602439709171	0.3431927240825883	0.6964654488839612	0.9455491987163788	0.6854256540363772	0.3723335247731316
		N	44	44	45	44	44	44	44	44	44	44	43
nMedial_frontal_cortextilavuus_DX	mri__t1__cross__volume__medial_frontal_cortex_right_normalized	Pearson Correlation	0.04170671607012161	0.02390749370625907	-0.1741629517354271	0.04594618392622569	-0.1424184328824778	0.1611393815667522	-0.1043714957441556	0.04009993698312397	-0.2420239333523887	-0.03178725479433552	0.275480859736679
		Sig. (2-tailed)	0.7880807787745421	0.8775774862047251	0.2525345777803064	0.7671116881006079	0.3564171815778869	0.2960439480942224	0.5001609887186176	0.7960678931842685	0.1134621532528909	0.837701544648306	0.07377714820090489
		N	44	44	45	44	44	44	44	44	44	44	43
nMedial_frontal_cortextilavuus_SIN	mri__t1__cross__volume__medial_frontal_cortex_left_normalized	Pearson Correlation	-0.009532928892677294	0.1834616857277049	-0.05512791100891127	0.09482634976603863	-0.1419723383618652	0.2200162308932712	0.2062276601517338	-0.01485456458219362	-0.170898017342474	-0.1853341312819097	0.09147935768661744
		Sig. (2-tailed)	0.9510285278582464	0.2332363955336762	0.7190890735764364	0.5403518602987946	0.3579411722544565	0.1512698039985698	0.1792501701016215	0.9237567757647065	0.2673618448952131	0.2284179250623218	0.5596110461753211
		N	44	44	45	44	44	44	44	44	44	44	43
nMiddle_frontal_gyrustilavuus_DX	mri__t1__cross__volume__middle_frontal_gyrus_right_normalized	Pearson Correlation	0.1055471381733373	-0.06075257521383605	0.1021243434264094	0.1143136184123847	0.1341977351607357	0.03804775272976712	-0.1020744821295481	0.07344308141320578	0.03164258266070887	0.05666993039577326	0.05935059788218527
		Sig. (2-tailed)	0.4953188656972729	0.6952445119618096	0.5044262780734926	0.4599867760652341	0.3851324185666885	0.8062989828467246	0.5096910687182847	0.6356551622953285	0.8384303704829341	0.7148292996611535	0.7053910517395428
		N	44	44	45	44	44	44	44	44	44	44	43
nMiddle_frontal_gyrustilavuus_SIN	mri__t1__cross__volume__middle_frontal_gyrus_left_normalized	Pearson Correlation	-0.0026204947234041	0.02333296917076574	0.04454751244185125	0.1628113982244745	0.1920360635053168	0.05310786411095066	0.0829651081168463	0.09019071576358982	0.0008133850305021774	0.2667388038233377	0.06392898796845392
		Sig. (2-tailed)	0.9865307543850497	0.8804978763046821	0.771381110252339	0.2909939110138164	0.2117332059425527	0.7320664487108203	0.592366546542301	0.5604162622070697	0.9958190581771043	0.08007082712821806	0.6838063431945979
		N	44	44	45	44	44	44	44	44	44	44	43
nMiddle_occipital_gyrustilavuus_DX	mri__t1__cross__volume__middle_occipital_gyrus_right_normalized	Pearson Correlation	-0.08017078322750985	0.007332522288000983	-0.2618937342000029	-0.1325296133012806	-0.01811528224228997	-0.08891616243175204	-0.03864356619673057	-0.07662011284444119	.437**	0.02597559493444303	-0.138856568547594
		Sig. (2-tailed)	0.6049346378968554	0.962322887845807	0.0822327834283559	0.3911210909172389	0.9070871938702327	0.5659932149980029	0.8033252024367419	0.6210684257147369	0.003011482403416425	0.8670785903537118	0.3745272189033224
		N	44	44	45	44	44	44	44	44	44	44	43
nMiddle_occipital_gyrustilavuus_SIN	mri__t1__cross__volume__middle_occipital_gyrus_left_normalized	Pearson Correlation	-0.04016934976064444	0.02453579047675863	-0.05548177506698235	-0.05113907715495061	-0.01256705727970971	-0.09114585401589388	-0.1370396444181605	-0.1430012351390829	0.0638625542290162	.307*	-.359*
		Sig. (2-tailed)	0.7957224179953937	0.8743856028625125	0.7173603994437264	0.7416504229573482	0.9354707863048997	0.5562538402110061	0.3750550685393379	0.3544321054209623	0.6804552988801024	0.04244864782564333	0.01813900102623395
		N	44	44	45	44	44	44	44	44	44	44	43
nMedial_orbital_gyrustilavuus_DX	mri__t1__cross__volume__medial_orbital_gyrus_right_normalized	Pearson Correlation	.356*	-0.166742213555371	-0.1308049723544892	0.181055228690802	.307*	-0.002721728681350555	-0.07126513658449662	-0.001107747935202034	-0.2081097795766099	0.06226638760581932	0.08189885681627758
		Sig. (2-tailed)	0.01771704523386247	0.2793427714477406	0.3917418641526782	0.2395304299857058	0.04260050341184188	0.9860104660353104	0.6457345206567612	0.9943060026467946	0.1752266708562741	0.6880313474690097	0.6016007100074985
		N	44	44	45	44	44	44	44	44	44	44	43

			Motor Fitness	Muscular strenght		Screen time				Physical activity			
nMedial_orbital_gyrustilavuus_SIN	mri__t1__cross__volume__medial_orbital_gyrus_left_normalized	Pearson Correlation	0.1499925977617153	0.02858229903707244	-0.2503387263839915	-0.02959024243792835	0.01865165688380794	-0.2540987481156972	0.1866954248426306	0.01021775424614946	-0.1184522829419878	-0.1507190517626125	0.1400217013552471
		Sig. (2-tailed)	0.3311460793768919	0.8538777710877963	0.09719753815735795	0.8487837137561516	0.9043486986533749	0.09602823951856755	0.224958016889446	0.9475152520373811	0.4437918218236656	0.3287824817618188	0.3704928333574911
		N	44	44	45	44	44	44	44	44	44	44	43
nPostcentral_gyrus_medial_segmenttilavuus_DX	mri__t1__cross__volume__postcentral_gyrus_medial_segment_right_normalized	Pearson Correlation	-0.03298997775951596	0.07386058595578505	-0.08290765039917262	-0.05243853791785508	-0.09006559025399814	-0.0003385865320053068	-0.09239667420268731	-0.1084960435809889	0.1437896527622486	-0.01748045908326125	-0.1483330602654599
		Sig. (2-tailed)	0.8316477458060638	0.6337303193664784	0.5882011882159577	0.7353202674122009	0.5609626253274167	0.9982595994503545	0.5508249390927494	0.4832802060567902	0.3517574191931722	0.9103296978920857	0.3424740495043974
		N	44	44	45	44	44	44	44	44	44	44	43
nPostcentral_gyrus_medial_segmenttilavuus_SIN	mri__t1__cross__volume__postcentral_gyrus_medial_segment_left_normalized	Pearson Correlation	-0.1486706251140165	0.1011194018594445	-0.1738486940901836	0.1331057526358739	0.06522741609496946	0.2117411453862945	-0.1568105896978789	-.384*	0.04695862122697551	0.05933355176223627	-.380*
		Sig. (2-tailed)	0.3354743567631503	0.5136803825148629	0.2534049474921511	0.389046578804056	0.6740016409179054	0.1676480355030448	0.3093793429197445	0.01005469580699966	0.7621276146644645	0.7020302562419309	0.01195196344214994
		N	44	44	45	44	44	44	44	44	44	44	43
nPrecentral_gyrus_medial_segmenttilavuus_DX	mri__t1__cross__volume__precentral_gyrus_medial_segment_right_normalized	Pearson Correlation	-0.0928757056391171	0.1940504226121608	0.1042111446959229	0.02666572529553836	-0.0934859318589257	-0.007370794443844205	-0.01176279947662691	0.1548196308307266	0.177461066440347	0.1764394469068281	-0.03575376189986035
		Sig. (2-tailed)	0.5487524872010358	0.2068887260865118	0.4957094068790635	0.8635800153303037	0.5461178273031863	0.9621263739198901	0.9395926080061842	0.3156391483603595	0.2491441038089229	0.2519235166906633	0.819943898840118
		N	44	44	45	44	44	44	44	44	44	44	43
nPrecentral_gyrus_medial_segmenttilavuus_SIN	mri__t1__cross__volume__precentral_gyrus_medial_segment_left_normalized	Pearson Correlation	-0.2029492962547065	0.2678588272296488	0.1629632495998848	-0.06615431353520004	-0.1360089007527417	0.1153910473714193	-0.1245756709756747	0.1183190420861238	0.2029699521906566	0.1298996550549153	-0.04891982667848982
		Sig. (2-tailed)	0.1864157943490327	0.07876367150339662	0.2848001569278186	0.669631979482239	0.3786917671091725	0.4557402909044221	0.4204168703974529	0.4443082643268494	0.1863700168348981	0.4006727156009966	0.7554033473243464
		N	44	44	45	44	44	44	44	44	44	44	43
nSuperior_frontal_gyrus_medial_segmenttilavuus_DX	mri__t1__cross__volume__superior_frontal_gyrus_medial_segment_right_normalized	Pearson Correlation	-0.07722533936491419	0.1907362613718127	-0.1689617103998766	0.02775523196544216	-0.0432936066700224	-0.101794300559097	0.260794268127632	0.1226190248739229	-0.2859362829147166	0.1084230702605628	0.1210977487864952
		Sig. (2-tailed)	0.618305624602574	0.2149008187258552	0.2671994303033679	0.8580620950796581	0.7802135467641995	0.5108597482987327	0.0872951395045117	0.4278091564224895	0.05989303941611663	0.4835762539614403	0.4392000655897971
		N	44	44	45	44	44	44	44	44	44	44	43
nSuperior_frontal_gyrus_medial_segmenttilavuus_SIN	mri__t1__cross__volume__superior_frontal_gyrus_medial_segment_left_normalized	Pearson Correlation	0.02297199004632974	-0.03016940941992195	-0.1610126106326281	0.06997634855401644	0.09248691129430388	-0.1494602022324371	.333*	-0.121026797197443	-0.2649226268104019	-0.0987996234530439	-0.0721492953735068
		Sig. (2-tailed)	0.8823335887407506	0.8458593919415321	0.2906830418464149	0.651728744450788	0.5504342593918515	0.3328849938063418	0.02736928638629837	0.433878090133146	0.08222648436390761	0.5234347443592549	0.6456789666373652
		N	44	44	45	44	44	44	44	44	44	44	43
nMiddle_temporal_gyrustilavuus_DX	mri__t1__cross__volume__middle_temporal_gyrus_right_normalized	Pearson Correlation	-0.1984876977502301	0.198250854730713	-0.1812565252768291	0.05984233265362607	-0.1054228741577313	0.09088244735743332	0.1244924547906905	-0.1549657280765204	0.0009966330932893962	0.008411949506419849	-0.256057904341468
		Sig. (2-tailed)	0.1964920356140485	0.1970374679430159	0.2334217446046112	0.699594608810899	0.4958295292531991	0.5574002943768789	0.4207297844210507	0.3151770954226071	0.9948771422982137	0.9567813017047073	0.09744538405265729
		N	44	44	45	44	44	44	44	44	44	44	43
nMiddle_temporal_gyrustilavuus_SIN	mri__t1__cross__volume__middle_temporal_gyrus_left_normalized	Pearson Correlation	-0.08621874718031422	0.06596516309547171	-0.04504712204017183	-0.08343688083851895	-0.1287965265660606	-0.1590554002011624	0.1697206579588715	-0.1949444205682717	-0.04593349966393821	0.04147412826612035	-0.2319710680107006
		Sig. (2-tailed)	0.5778799706318223	0.6705228200648661	0.7688871151942338	0.5902560908288755	0.4047189703546941	0.3024169097136714	0.2707209725283666	0.2047637222486539	0.7671741901713167	0.7892356384689341	0.1344414946079455
		N	44	44	45	44	44	44	44	44	44	44	43
nOccipital_poletilavuus_DX	mri__t1__cross__volume__occipital_pole_right_normalized	Pearson Correlation	0.09670621714008383	-0.05524556841403608	-0.0613258150134051	.371*	.356*	-0.01102565203695456	0.2843054559174188	0.06273906240180037	-0.1138312158557895	-0.03274532179950388	0.1582445939411148
		Sig. (2-tailed)	0.5323151558249407	0.7217056565091124	0.6890190519899039	0.01324191102415305	0.01781948657482059	0.9433718620494781	0.06143092707241062	0.6857846556496158	0.4618949690079148	0.8328784320246163	0.3108186351556935
		N	44	44	45	44	44	44	44	44	44	44	43
nOccipital_poletilavuus_SIN	mri__t1__cross__volume__occipital_pole_left_normalized	Pearson Correlation	-0.005118983130062812	0.04826195464133257	0.04353573836169131	0.1936818737063245	0.03128839222128251	.370*	-0.2337246307470292	-0.2433160243680941	-0.1383022258537057	0.02736419931187445	-0.2673300252002674
		Sig. (2-tailed)	0.9736920081130448	0.7557255026174976	0.7764387363360129	0.2077692293637009	0.8402152653993314	0.01351107318357057	0.1267713607001501	0.1114893020565297	0.3706288515902543	0.8600417660413627	0.08308821102859135
		N	44	44	45	44	44	44	44	44	44	44	43
nOccipital_fusiform_gyrustilavuus_DX	mri__t1__cross__volume__occipital_fusiform_gyrus_right_normalized	Pearson Correlation	-0.05378400129649012	-0.1219121472416625	-0.1282644220571268	-0.1217464512747236	-0.09353446439498986	-0.0743955596916391	0.1329128957559233	-0.1916215616647343	0.03546253176976551	0.02311076296395121	-0.1461387163107231
		Sig. (2-tailed)	0.7287842269275551	0.4304975904791548	0.401083481058945	0.4311291382803122	0.5459085471454302	0.6312673907113097	0.3897402812205466	0.2127397927977794	0.8192329241201036	0.8816278044900366	0.3497414677876374
		N	44	44	45	44	44	44	44	44	44	44	43
nOccipital_fusiform_gyrustilavuus_SIN	mri__t1__cross__volume__occipital_fusiform_gyrus_left_normalized	Pearson Correlation	-0.09253240793003789	-0.1218400220270332	0.07078968156041777	-0.04258977258600091	-0.1375866933863078	0.09363424543011964	0.0684409423064475	-0.08678389558968819	0.006711321096298097	0.08892803668011405	-0.1145131665743878
		Sig. (2-tailed)	0.5502373324767652	0.4307724310787433	0.6440174171162886	0.7837002770729571	0.3731334342613053	0.5454783955930422	0.6588983921740548	0.5753801672325467	0.9655128356497418	0.5659411396146066	0.4646592226446538
		N	44	44	45	44	44	44	44	44	44	44	43
nOpercular_part_of_the_inferior_frontal_gyrustilavuus_DX	mri__t1__cross__volume__opercular_part_of_the_inferior_frontal_gyrus_right_normalized	Pearson Correlation	0.07789882762312701	-0.006771879815429594	-0.09955476850020271	0.1479408698709177	.385**	-0.1082514110867198	-0.1860791888297805	0.2116770576679685	0.1463111144734128	-0.09267102902175949	0.07923989950465629
		Sig. (2-tailed)	0.615237319169654	0.9652018346627551	0.515265868433542	0.3378786206459884	0.009793572084662461	0.4842730376268777	0.2265197656951774	0.1677796950398516	0.3432864374097351	0.5496375333849529	0.6134922544179379
		N	44	44	45	44	44	44	44	44	44	44	43
nOpercular_part_of_the_inferior_frontal_gyrustilavuus_SIN	mri__t1__cross__volume__opercular_part_of_the_inferior_frontal_gyrus_left_normalized	Pearson Correlation	-0.04790098536492762	0.1776420140050528	-.348*	0.1191869912559958	0.005490201549394997	-0.08643244524176445	0.1850579626514437	0.1650158838515797	-0.06702461518021699	0.1006652781701419	0.2424897357411717
		Sig. (2-tailed)	0.7574970243705987	0.2486539809680057	0.01931932049841593	0.4409500287474704	0.9717849458650035	0.5769341476878628	0.2291242762227708	0.2844214921543434	0.6655389113670029	0.5155827082445926	0.1171750936115463
		N	44	44	45	44	44	44	44	44	44	44	43

			Motor Fitness	Muscular strenght		Screen time				Physical activity			
nOrbital_part_of_the_inferior_frontal_gyrustilavuus_DX	mri__t1__cross__volume__orbital_part_of_the_inferior_frontal_gyrus_right_normalized	Pearson Correlation	-0.04791931782366272	-0.05807041797867862	-0.03786562160810638	-0.2570469789797784	-0.1440302219396305	-0.1525970891518094	-0.2546695401640257	0.2221311256886086	0.06374957420411106	0.03851870947820956	0.169964846241586
		Sig. (2-tailed)	0.7574070248443859	0.7080898793507293	0.8049462073225961	0.09210328315937029	0.3509437542353229	0.3227210728471766	0.09525844998353754	0.14727921624409	0.680990537237854	0.803948152386838	0.2758700214069291
		N	44	44	45	44	44	44	44	44	44	44	43
nOrbital_part_of_the_inferior_frontal_gyrustilavuus_SIN	mri__t1__cross__volume__orbital_part_of_the_inferior_frontal_gyrus_left_normalized	Pearson Correlation	-0.1661566987089647	0.1850019065469906	0.08914521205859217	-0.1717625463907265	-0.172600902507383	-0.1305886548771028	-0.1547960834441229	0.09714289683056919	0.1441312079598612	0.02744155326571032	-0.08236920297008457
		Sig. (2-tailed)	0.2810586047019342	0.2292678325503437	0.560339243688981	0.2649129150992951	0.2625523955266387	0.3981574064553313	0.3157136603571893	0.5304566727521884	0.3506025372386612	0.8596500805007409	0.5995076682306224
		N	44	44	45	44	44	44	44	44	44	44	43
nPosterior_cingulate_gyrustilavuus_DX	mri__t1__cross__volume__posterior_cingulate_gyrus_right_normalized	Pearson Correlation	0.01245150991013546	0.07091424709184885	0.1292869769550551	-0.005225745762930665	-0.08470168742646049	0.02081566427475488	-0.05017382440029838	-0.06350164570783819	-0.03143942859253396	-0.1273340240438462	-0.1583411787856775
		Sig. (2-tailed)	0.9360628666235562	0.647364351736638	0.3973081298266292	0.9731435205910497	0.5846145675079555	0.8933116700367062	0.7463634833525457	0.6821656310276518	0.8394540406126574	0.4101195453497403	0.3105196214181317
		N	44	44	45	44	44	44	44	44	44	44	43
nPosterior_cingulate_gyrustilavuus_SIN	mri__t1__cross__volume__posterior_cingulate_gyrus_left_normalized	Pearson Correlation	0.1000794626206463	0.01612844058161415	-0.1122134265562	-0.02176158574198749	-0.1288817455857127	-0.03741788048489035	0.08069853657407845	-0.002538128363122541	-.434**	-0.006558060956670101	0.1420338218671546
		Sig. (2-tailed)	0.5180418855870531	0.9172401751058811	0.4630153610722609	0.8884933357885976	0.4044055518106572	0.8094456918501103	0.6025521388516905	0.9869540774664802	0.003209698403802797	0.9662999298472948	0.3635871527782235
		N	44	44	45	44	44	44	44	44	44	44	43
nPrecuneustilavuus_DX	mri__t1__cross__volume__precuneus_right_normalized	Pearson Correlation	-0.07532269153873347	0.1849387289301102	-0.2852789198437171	0.1256453308545714	-0.009713597505838613	0.1256618280039775	0.1463617839855645	-0.1635604450805461	-0.01382974466668512	0.1067441855484353	-0.1274780055841585
		Sig. (2-tailed)	0.62700837523758	0.2294297005692512	0.05749345481371822	0.4164064225055322	0.9501015739523735	0.416344741046955	0.3431175087291868	0.2887497646887376	0.9290029150626746	0.4904134924059174	0.4152851758534334
		N	44	44	45	44	44	44	44	44	44	44	43
nPrecuneustilavuus_SIN	mri__t1__cross__volume__precuneus_left_normalized	Pearson Correlation	-0.1933962225175629	0.1855178681226813	-0.1393802029575263	-0.03557669550258082	-0.137050859861673	-0.01432774732529629	0.1585881364846889	0.02584793370381785	0.08423067310858058	0.01230292513409161	-0.05240451335313363
		Sig. (2-tailed)	0.2084534803823175	0.2279488136849968	0.3611645975344917	0.8186607310339067	0.3750156126579798	0.9264532124009015	0.3038587814628609	0.8677260385794028	0.5867126470406068	0.9368242851180639	0.7385736699962732
		N	44	44	45	44	44	44	44	44	44	44	43
nParahippocampal_gyrustilavuus_DX	mri__t1__cross__volume__parahippocampal_gyrus_right_normalized	Pearson Correlation	6.028704820226113e-05	-0.005162167692677764	-0.155089105311219	-0.1923400284947589	-0.1490268057865627	-0.2460882883732687	0.1776001125564947	-0.08912374734587047	-0.1829439091783869	-0.1271729044400581	-0.1956892428294562
		Sig. (2-tailed)	0.999690112631314	0.9734701481908383	0.3090274997470568	0.2109971611748905	0.3343047436218725	0.1073445687643843	0.2487674192835605	0.5650831557568339	0.234580988684818	0.4107170241329574	0.208533464282458
		N	44	44	45	44	44	44	44	44	44	44	43
nParahippocampal_gyrustilavuus_SIN	mri__t1__cross__volume__parahippocampal_gyrus_left_normalized	Pearson Correlation	-0.1368756840651384	0.1109625962963459	-0.2350299661350851	-0.1905537071233815	-0.195320108453867	-0.2078612867311963	0.1926444092737152	-0.0898589671808832	0.05877289102198455	0.2562864564532442	-0.1826209096297636
		Sig. (2-tailed)	0.3756321625659182	0.4733295778760889	0.1201553051142652	0.2153483246666163	0.2038753051760214	0.1757541323252247	0.2102618985056981	0.5618653928141031	0.7047176835104974	0.0931036666878778	0.241146308598542
		N	44	44	45	44	44	44	44	44	44	44	43
nPosterior_insulatilavuus_DX	mri__t1__cross__volume__posterior_insula_right_normalized	Pearson Correlation	-0.1919959150424028	0.2364705077707027	-0.2121098836566301	0.1613717290265018	-0.09161706266250856	0.2262652046434585	0.0841829148618848	-0.05847419683050058	-0.04203687709165096	0.2829214608635979	-0.01035957725608342
		Sig. (2-tailed)	0.2118305581352221	0.1222443223409064	0.1618696031779852	0.2953388216208224	0.5542057158227719	0.1397035238153346	0.5869255683222535	0.7061508749424485	0.7864422136308545	0.06276062047082485	0.9474317807079663
		N	44	44	45	44	44	44	44	44	44	44	43
nPosterior_insulatilavuus_SIN	mri__t1__cross__volume__posterior_insula_left_normalized	Pearson Correlation	-0.1877533893697169	0.2425717529496418	-0.2188891530680786	0.1505109736114116	-0.06240661790635826	0.2078168357904824	-0.02339670554937988	0.05459409598657848	0.01957857534613611	0.05167427087931035	0.09755863892729497
		Sig. (2-tailed)	0.2222940998998066	0.1126224944620282	0.1485650984425784	0.3294584070245246	0.6873645345648011	0.1758486059773937	0.8801738172296517	0.7248580307243435	0.8996188582511117	0.7390412277363481	0.533697883219924
		N	44	44	45	44	44	44	44	44	44	44	43
nParietal_operculumtilavuus_DX	mri__t1__cross__volume__parietal_operculum_right_normalized	Pearson Correlation	-0.1351392256768288	0.253595778530553	-0.1332042192248926	0.1341109346633387	-0.01164535574208322	0.1216326493229754	-0.171129250633768	-0.03569744858426918	0.0382549121822276	0.08100946639436697	0.03027192953697816
		Sig. (2-tailed)	0.3817763880604966	0.09671053006914981	0.3830378764582956	0.385442699405806	0.940194640296527	0.4315631925641307	0.2667053716648936	0.8180556131629592	0.8052647153950763	0.6011503813179729	0.847193165407332
		N	44	44	45	44	44	44	44	44	44	44	43
nParietal_operculumtilavuus_SIN	mri__t1__cross__volume__parietal_operculum_left_normalized	Pearson Correlation	-0.06952906010826358	0.1909593726908828	-0.1770100347403289	0.005102220352848428	-0.1405700110343904	0.1995701735770394	0.03295536285798457	-.346*	-0.06899999036391996	0.2947072779214644	-0.2376121666610442
		Sig. (2-tailed)	0.6538142128331413	0.2143547705193978	0.2447408589233364	0.9737781271122509	0.362757618592467	0.1940127395380077	0.8318218441155643	0.02159747125629062	0.6562843504033903	0.05214180864130809	0.1249604436484672
		N	44	44	45	44	44	44	44	44	44	44	43
nPostcentral_gyrustilavuus_DX	mri__t1__cross__volume__postcentral_gyrus_right_normalized	Pearson Correlation	-0.04208989687278786	0.00559883615558862	-0.05476055563617042	-0.1050986345774727	-0.1229271574101326	-0.04320483095853789	-0.1872821285905233	-0.1170853809833737	-0.1464834757151042	0.009854104688952674	-8.78577435017394e-05
		Sig. (2-tailed)	0.7861791650341639	0.971226885694611	0.7208851222628218	0.4971632674139461	0.4266402056856216	0.7806531004179909	0.2234780162795978	0.4491056124361384	0.3427120059974866	0.949380720648047	0.9995538672162968
		N	44	44	45	44	44	44	44	44	44	44	43
nPostcentral_gyrustilavuus_SIN	mri__t1__cross__volume__postcentral_gyrus_left_normalized	Pearson Correlation	-.320*	0.2698931433094746	-0.02006028596877388	0.0495421157871485	-0.2619227157361497	.361*	-0.1702317929336024	-0.2924106545575316	0.1030208362555048	0.02571044904389535	-0.2818245167474879
		Sig. (2-tailed)	0.03409969792723113	0.07643231241193653	0.895937657228593	0.7494529106977125	0.08588614946065697	0.01603369231392808	0.2692592435174882	0.05408861786944506	0.5057536783189039	0.8684234030593999	0.06711551550902622
		N	44	44	45	44	44	44	44	44	44	44	43
nPosterior_orbital_gyrustilavuus_DX	mri__t1__cross__volume__posterior_orbital_gyrus_right_normalized	Pearson Correlation	0.08291430961223678	0.01120881112645499	-0.04976354153601182	0.144173577732966	0.03240151338879208	0.02813464485415764	0.05389509575706089	-0.1950343976178649	-0.1614398994167732	-0.1271358328389227	-0.02123710396072185
		Sig. (2-tailed)	0.5925939903612968	0.9424327125394971	0.7454601040196607	0.3504594363660418	0.8346085488052639	0.8561420730482905	0.7282453892730925	0.2045507001678213	0.2951321444528383	0.410854566987069	0.8924754699409456
		N	44	44	45	44	44	44	44	44	44	44	43
nPosterior_orbital_gyrustilavuus_SIN	mri__t1__cross__volume__posterior_orbital_gyrus_left_normalized	Pearson Correlation	-0.07353746179962575	0.1316211543265171	0.0272130695281098	-0.006726408807743113	-0.1796397692596417	0.1215829366389732	-0.1404335844578531	-0.1717628891984084	-0.1078787914214155	-0.03844988876689636	-0.1445986391566032
		Sig. (2-tailed)	0.6352198273331288	0.3944053094611887	0.8591576312379496	0.9654353518131282	0.2432859558534015	0.4317528792812079	0.3632282682846699	0.2649119470010222	0.4857873457634427	0.8042915721795099	0.3548978648919832
		N	44	44	45	44	44	44	44	44	44	44	43

			Motor Fitness	Muscular strenght		Screen time				Physical activity			
nPlanum_polaretilavuus_DX	mri__t1__cross__volume__planum_polare_right_normalized	Pearson Correlation	-0.21186958730554	0.1532952227277301	-0.1451056268914285	-0.04919580693142001	-0.0630468464041294	-0.08482866485858875	0.0496281336152779	-0.001647889718932544	0.08064197643834065	0.2528780978717377	-0.07797813209105865
		Sig. (2-tailed)	0.167384394131489	0.3204858582520134	0.3415775264922232	0.7511482142516295	0.6843231500747851	0.5840495371056501	0.7490320036741906	0.9915296735892973	0.6028072799968941	0.0976905435221154	0.6191699047119054
		N	44	44	45	44	44	44	44	44	44	44	43
nPlanum_polaretilavuus_SIN	mri__t1__cross__volume__planum_polare_left_normalized	Pearson Correlation	-0.01279138797058652	-0.04826699425258135	-0.1302715171926179	0.1460974560780122	0.07410488312467675	0.1060027680479603	0.0677426928777185	-0.09692587163665757	0.06843936982162062	0.1461037647880156	-0.07985554565848832
		Sig. (2-tailed)	0.934321384054761	0.7556863145930784	0.393692742171895	0.3439993229267174	0.6326051291697463	0.49344877124137	0.6621689695052753	0.5313799221919653	0.6589057506107691	0.3439782604534232	0.610730049067499
		N	44	44	45	44	44	44	44	44	44	44	43
nPrecentral_gyrustilavuus_DX	mri__t1__cross__volume__precentral_gyrus_right_normalized	Pearson Correlation	-0.173718957109406	0.2504407137224596	-0.1702675165864208	0.1480532233837637	0.2775381980422083	0.06425675180612629	-0.1445110814805917	0.1015847871764319	0.2522474365143087	0.2234617724509436	0.02159626351419574
		Sig. (2-tailed)	0.2594261883019603	0.1010760253435051	0.263465730604493	0.3375077658169274	0.06815215167046679	0.6785890196279665	0.3493208167436956	0.5117345412222792	0.09855802676537839	0.144808499973746	0.8906679372419918
		N	44	44	45	44	44	44	44	44	44	44	43
nPrecentral_gyrustilavuus_SIN	mri__t1__cross__volume__precentral_gyrus_left_normalized	Pearson Correlation	-0.13213964560799	0.201398991814073	0.2513562191161075	0.1108275832301687	0.1111323849603522	0.1591963480395454	-0.1088815769070274	0.02816085108879857	0.2107958698417318	0.08712895979941011	-0.1586482865796419
		Sig. (2-tailed)	0.3925289187146468	0.1898744535993174	0.09580097178312127	0.4738714250036313	0.4726486326093871	0.3019831291640364	0.4817176982622489	0.8560094863461324	0.1695975701731074	0.5738562811954941	0.3095700720598907
		N	44	44	45	44	44	44	44	44	44	44	43
nPlanum_temporaletilavuus_DX	mri__t1__cross__volume__planum_temporale_right_normalized	Pearson Correlation	-0.1108050880294699	0.1191114305499733	0.04203781073508987	0.165924947453134	0.06476293175166584	0.1201238259913138	0.07867929473857754	0.04469209547317095	-0.0677133697590621	0.1806741461294681	0.1673126039508382
		Sig. (2-tailed)	0.4739617367837736	0.4412418269962128	0.7839432928419505	0.2817396451581788	0.6761953582060675	0.4373410509093925	0.61168973296231	0.7732982497547973	0.6623064545423661	0.2405376275248149	0.2835430000951348
		N	44	44	45	44	44	44	44	44	44	44	43
nPlanum_temporaletilavuus_SIN	mri__t1__cross__volume__planum_temporale_left_normalized	Pearson Correlation	-0.02333593202931709	0.1285837576989417	-0.1525198794053282	0.08799129132745832	0.05653537561987563	0.05803011963364711	0.01771507319749237	-.320*	-0.2461922519112281	0.2109457732320466	-0.09438934293184309
		Sig. (2-tailed)	0.8804828116195892	0.4055021026343435	0.3172086026294253	0.5700560992408868	0.7154779429342578	0.708283498203349	0.9091311823070727	0.03427068971269458	0.1071914563394276	0.1692873216994739	0.5471340412084031
		N	44	44	45	44	44	44	44	44	44	44	43
nSubcallosal_areatilavuus_DX	mri__t1__cross__volume__subcallosal_area_right_normalized	Pearson Correlation	-0.001888530720077369	0.1042686177330386	0.01740488332340663	0.001970052937874516	0.01605310918298075	-0.02273936870104937	-0.1684095509852319	-0.04044064905823742	0.08478128027737732	0.02959522179105479	-0.1561923558157922
		Sig. (2-tailed)	0.9902928086149353	0.5005858612005931	0.9096512124696342	0.9898737994566746	0.9176253932282263	0.8835168778040461	0.2744943655918775	0.7943725016880301	0.5842603626794185	0.8487585634679337	0.3172153029834361
		N	44	44	45	44	44	44	44	44	44	44	43
nSubcallosal_areatilavuus_SIN	mri__t1__cross__volume__subcallosal_area_left_normalized	Pearson Correlation	0.09753580441877197	0.01105847794476981	-0.006149503924107097	0.08934452853134907	0.1346055556245569	-0.07316330006644357	-0.1576665792940871	-0.0623235737934051	0.073354788729517	0.1437618653182459	-0.1792284698717617
		Sig. (2-tailed)	0.5287871994456589	0.9432035413611184	0.9680200768935332	0.5641159883345228	0.383676576575674	0.6369463825748297	0.3067124884485167	0.6877593916161174	0.6360625272640511	0.3518514773811325	0.2501484557691872
		N	44	44	45	44	44	44	44	44	44	44	43
nSuperior_frontal_gyrustilavuus_DX	mri__t1__cross__volume__superior_frontal_gyrus_right_normalized	Pearson Correlation	0.03021019940034037	0.06817933572880547	-0.04796043891199166	0.2300452975545766	.383*	-0.07390049716140219	0.2407393448819231	0.1311691139489156	-0.07004858172412118	0.2518733841652249	0.103495740592699
		Sig. (2-tailed)	0.8456535123871459	0.6601230164760744	0.7543908233207599	0.1330330196246031	0.01033311446538389	0.633546439425258	0.1154497072881501	0.3960454774600408	0.6513922051194303	0.09907533397065099	0.5089678394125323
		N	44	44	45	44	44	44	44	44	44	44	43
nSuperior_frontal_gyrustilavuus_SIN	mri__t1__cross__volume__superior_frontal_gyrus_left_normalized	Pearson Correlation	-0.1040243784984794	0.1346783404310056	-0.01098609836283255	0.1769776048306598	.324*	-0.1767804934881325	0.1294704397107745	0.2941592695541512	0.02125896443295389	-0.05679125617061726	0.2319076225781979
		Sig. (2-tailed)	0.50159527484489	0.383417090483239	0.9429001516257328	0.2504568210046068	0.03215983559195865	0.2509933599322036	0.4022442777719355	0.05260113658942957	0.8910531047023909	0.7142445990781744	0.1345510891529769
		N	44	44	45	44	44	44	44	44	44	44	43
nSupplementary_motor_cortextilavuus_DX	mri__t1__cross__volume__supplementary_motor_cortex_right_normalized	Pearson Correlation	-0.1216142851281998	0.2659795132464038	-0.008710862138444011	0.2408581357730014	.327*	0.05399639405071108	0.059929306399457	0.1022129051776708	-0.1794014400950543	-0.1377986393115165	0.1613684273067659
		Sig. (2-tailed)	0.4316332586711324	0.08096659740467729	0.9547117492991322	0.1152648109554193	0.03020834763154075	0.7277541784222727	0.6991785432794136	0.5091141834982206	0.2439221963162163	0.3723905080308134	0.3012403796564488
		N	44	44	45	44	44	44	44	44	44	44	43
nSupplementary_motor_cortextilavuus_SIN	mri__t1__cross__volume__supplementary_motor_cortex_left_normalized	Pearson Correlation	-0.2556599079973921	.440**	-0.04824655467186859	.314*	.466**	-0.06521644150456163	0.0403304641714016	0.1556058238434908	0.06465972331651415	0.01830706794848171	0.03006090195396886
		Sig. (2-tailed)	0.093934117957734	0.002828192971994875	0.7529715493047381	0.03769897436063382	0.001429817563469608	0.6740534421950374	0.7949206823286519	0.313157755309837	0.6766831629654994	0.9061078956884999	0.8482458860479756
		N	44	44	45	44	44	44	44	44	44	44	43
nSupramarginal_gyrustilavuus_DX	mri__t1__cross__volume__supramarginal_gyrus_right_normalized	Pearson Correlation	0.1339257122123607	-0.09538044464284874	-0.1645835102985795	-0.03535985702534616	0.1455836535994724	-0.1895980701624589	0.04586325653036746	0.118330563763389	-0.0263928726440788	0.1228716909627891	0.2098293715537359
		Sig. (2-tailed)	0.3861052956631987	0.5379769591774333	0.2799731385684228	0.8197476132054196	0.3457173846483537	0.217701491842164	0.7675203416662196	0.4442635931716751	0.8649629213814355	0.4268504940865898	0.1768594369242023
		N	44	44	45	44	44	44	44	44	44	44	43
nSupramarginal_gyrustilavuus_SIN	mri__t1__cross__volume__supramarginal_gyrus_left_normalized	Pearson Correlation	-0.2553307038733655	0.2662888060097391	-0.227199782706107	-0.1557079003247733	-0.09577167946854523	-0.0803688123970534	-0.03812604171784233	.299*	.441**	0.1295651058647184	0.01583382598880336
		Sig. (2-tailed)	0.09437274554464518	0.08060076873217692	0.1333761725062165	0.312836490493193	0.5363031383795972	0.6040401743276999	0.8059080775645858	0.0489448782654648	0.002757374776633654	0.4018973539339783	0.9197282635032311
		N	44	44	45	44	44	44	44	44	44	44	43
nSuperior_occipital_gyrustilavuus_DX	mri__t1__cross__volume__superior_occipital_gyrus_right_normalized	Pearson Correlation	-0.1513522208423865	0.2773651706230816	0.2480344097903682	0.1354372268770855	0.0964734471473476	0.02860901094519241	0.1656237862590872	0.1687757515666324	0.005507904475679878	-0.0660225070396	0.04700640480355066
		Sig. (2-tailed)	0.3267310166529778	0.06833134521929636	0.1004183458598659	0.3807177488317587	0.533307107717265	0.8537426947370667	0.2826262692339125	0.2734369660915433	0.9716940043335104	0.6702527004384462	0.7646927551680609
		N	44	44	45	44	44	44	44	44	44	44	43
nSuperior_occipital_gyrustilavuus_SIN	mri__t1__cross__volume__superior_occipital_gyrus_left_normalized	Pearson Correlation	0.06264528978074312	-0.0810331074464432	0.002605244558281634	0.1108594076659213	0.004817521434783728	0.1111758139527924	0.07009503162599451	0.01223976581210584	0.0561455449242135	-0.05835407035751324	0.04164792863255939
		Sig. (2-tailed)	0.6862301584327064	0.601043858998181	0.9864487953496025	0.4737436745940313	0.9752408159405895	0.4724745421585308	0.6511758279700989	0.9371479601874224	0.7173583019885679	0.7067275482002373	0.790877600464613
		N	44	44	45	44	44	44	44	44	44	44	43

			Motor Fitness	Muscular strenght		Screen time				Physical activity			
nSuperior_parietal_lobuletilavuus_DX	mri__t1__cross__volume__superior_parietal_lobule_right_normalized	Pearson Correlation	-0.06992943929336709	0.1974886581427859	-0.2569154437520748	.333*	0.09824433354614992	.358*	-0.02859999647281145	0.001989743816142591	0.1314012112946899	-0.1060511685679843	-0.1009756891652648
		Sig. (2-tailed)	0.6519473345668436	0.1987999996262773	0.08844239504360693	0.02739574423195851	0.5257831730011704	0.01705789428426254	0.8537882785153792	0.9897725923748165	0.3952028480123442	0.4932503297071027	0.5193936162424171
		N	44	44	45	44	44	44	44	44	44	44	43
nSuperior_parietal_lobuletilavuus_SIN	mri__t1__cross__volume__superior_parietal_lobule_left_normalized	Pearson Correlation	-0.2851983761725853	0.2539857952326381	-0.1440949859546715	-0.039002756458683	-0.2213800549728974	0.2208603899799752	-0.03786450211005947	0.2084257300826607	0.07495943442910304	-0.1347072449913628	0.176191036595071
		Sig. (2-tailed)	0.06058503771781701	0.09618113867071873	0.3449864459705119	0.8015337604340536	0.1486874281126792	0.1496675510547875	0.807214154987651	0.1745576658842926	0.6286756717740233	0.3833140711866796	0.2583979773113998
		N	44	44	45	44	44	44	44	44	44	44	43
nSuperior_temporal_gyrustilavuus_DX	mri__t1__cross__volume__superior_temporal_gyrus_right_normalized	Pearson Correlation	-0.09612625499229561	0.1106545502362685	0.08925925521573631	-0.1091960210442983	-0.09707699978478665	-0.1851641069832393	0.1458142135726861	0.03113193857744156	0.0648624222441189	0.2924955768233257	-0.09387177795638227
		Sig. (2-tailed)	0.5347883424622168	0.4745663374499589	0.5598356345331467	0.4804452680653066	0.5307369231437207	0.2288526158759538	0.3449457834073318	0.8410039451738388	0.675725249905493	0.05401560458818108	0.5493434528122826
		N	44	44	45	44	44	44	44	44	44	44	43
nSuperior_temporal_gyrustilavuus_SIN	mri__t1__cross__volume__superior_temporal_gyrus_left_normalized	Pearson Correlation	-0.08750655775867698	0.1820488654449365	-0.2889144394928035	0.04272999681041889	0.09685983966535533	-0.05179665336548933	0.1491039413015333	0.1060636424301702	-0.09559671279651652	0.0463225185012263	0.1028177708601219
		Sig. (2-tailed)	0.5721908332664984	0.2369177500794132	0.05425080651074087	0.7830052826112286	0.5316609852973345	0.7384449897104749	0.3340517829952051	0.4931991937035405	0.5370513863464365	0.7652579544691059	0.5117622662149448
		N	44	44	45	44	44	44	44	44	44	44	43
nTemporal_poletilavuus_DX	mri__t1__cross__volume__temporal_pole_right_normalized	Pearson Correlation	0.1666373204346958	0.03679133881830372	0.01958986233378868	-0.08999762418002966	-0.1253118689581163	0.141429373840806	-0.1558920896027597	-0.2554935167397705	-0.04452644907089449	0.04873653624473833	-0.232474502828427
		Sig. (2-tailed)	0.2796496517017472	0.8125787106224439	0.8983650420968486	0.5612595046991172	0.4176543222887953	0.3598014225825198	0.3122573219287526	0.09415561832888195	0.7741164504116799	0.7533982914042826	0.1335742243817419
		N	44	44	45	44	44	44	44	44	44	44	43
nTemporal_poletilavuus_SIN	mri__t1__cross__volume__temporal_pole_left_normalized	Pearson Correlation	-0.1896255486450678	0.2502903792271708	-0.07512705716922245	-0.2273160061712761	-0.1804239464602329	-0.0445054740065982	-0.03439742308409501	0.1158743751366364	0.08126386122357705	0.1268556310084843	0.04048764764047127
		Sig. (2-tailed)	0.2176335808585591	0.1012877489461653	0.6237942711782964	0.1378248441821526	0.2412004643113582	0.7742200727798834	0.8245757233366853	0.453842275528507	0.6000045558296807	0.4118950226452884	0.7965786035082881
		N	44	44	45	44	44	44	44	44	44	44	43
nTriangular_part_of_the_inferior_frontal_gyrustilavuus_DX	mri__t1__cross__volume__triangular_part_of_the_inferior_frontal_gyrus_right_normalized	Pearson Correlation	-0.1640401397375879	0.1717138202161954	0.02744633568398309	0.1727821890624245	0.08660283306866368	0.07914185163418297	0.1136005562280711	-0.170983483045026	0.0078571686601742776	0.1077192290738974	-0.0818991569035593
		Sig. (2-tailed)	0.2873185189047774	0.2650505430498742	0.8579626833484156	0.2620438022706441	0.5761805191213278	0.6095913254626517	0.4628088697292896	0.2671190824411639	0.9596292121440015	0.486436553581215	0.6015993736067796
		N	44	44	45	44	44	44	44	44	44	44	43
nTriangular_part_of_the_inferior_frontal_gyrustilavuus_SIN	mri__t1__cross__volume__triangular_part_of_the_inferior_frontal_gyrus_left_normalized	Pearson Correlation	-0.2056018024383938	0.2225406123865463	-0.08869262629654359	0.1652787663581034	0.1474853636671753	0.0303905382121476	0.2529546213397224	0.1883644862373271	0.09070852072770383	0.1593315862383455	0.120820191621783
		Sig. (2-tailed)	0.180602648921842	0.1465156050771865	0.5623399256276626	0.2836442519373128	0.3393847321705928	0.8447434089382446	0.09758568574627285	0.2207653379689499	0.5581579018321273	0.3015672959196629	0.440257404865501
		N	44	44	45	44	44	44	44	44	44	44	43
nTransverse_temporal_gyrustilavuus_DX	mri__t1__cross__volume__transverse_temporal_gyrus_right_normalized	Pearson Correlation	-0.1799110464509049	0.2673063269183185	-0.0580284089581329	.350*	0.1425607083577715	.307*	-0.01879675311390652	0.07005438153567722	-0.1927718993412943	0.02532926687371918	0.2738959101399094
		Sig. (2-tailed)	0.2425631266874951	0.07940637610870113	0.7049613718972246	0.0200005368206594	0.3559319567580401	0.0430126281283255	0.9036080875694156	0.651365186364137	0.2099544649230729	0.8703573907142094	0.07552020816729431
		N	44	44	45	44	44	44	44	44	44	44	43
nTransverse_temporal_gyrustilavuus_SIN	mri__t1__cross__volume__transverse_temporal_gyrus_left_normalized	Pearson Correlation	-0.1660345096286882	0.1744144487245062	0.01619579324364957	.372*	0.1750459409999549	.362*	-0.08234889043227943	-0.1819316195039766	-0.2585608139635273	0.07281514866282625	0.04129478815265997
		Sig. (2-tailed)	0.2814175437351209	0.2574941009572497	0.9159042910266811	0.01292692886631126	0.2557481593587017	0.01561217887587566	0.5951281836494522	0.237225023641059	0.0901367849848096	0.6385546223692977	0.7926116193765106
		N	44	44	45	44	44	44	44	44	44	44	43
nIsoaivojenHarmaaAinetilavuus_YHTEENSA	mri__t1__cross__volume__cerebral_gray_matter_total_normalized	Pearson Correlation	-.309*	.419**	-0.1667290622647694	0.1064760584491176	0.03669226990514281	0.06525360147105282	0.08168472641203486	0.02317728623064117	0.06181567369894137	0.2001946484335766	-0.07780445484177369
		Sig. (2-tailed)	0.04128857952775552	0.004622724190679288	0.2736641918594304	0.4915100561041783	0.813074370459187	0.6738780490723519	0.598111021889965	0.8812895063410849	0.6901761307829498	0.192592553998061	0.6199531363456566
		N	44	44	45	44	44	44	44	44	44	44	43
nIsoaivojenHarmaaAinetilavuus_DX	mri__t1__cross__volume__cerebral_gray_matter_right_normalized	Pearson Correlation	-0.1886738846495432	.354*	-0.196913768555805	0.1690572000511313	0.1355286035189412	0.06127645656478466	0.04141943487866843	0.02601582984215059	0.05851137476244869	0.2004569795079343	-0.06218323741002198
		Sig. (2-tailed)	0.2199941024260222	0.01845544275041772	0.1948021957703309	0.2726261136269533	0.3803934848251526	0.6927452216015659	0.789507270463751	0.8668745523040035	0.7059724333122885	0.1919981604412533	0.6920084421305667
		N	44	44	45	44	44	44	44	44	44	44	43
nIsoaivojenHarmaaAinetilavuus_SIN	mri__t1__cross__volume__cerebral_gray_matter_left_normalized	Pearson Correlation	-.417**	.464**	-0.126734138143317	0.03481314162227508	-0.06999132884555445	0.06788204166839441	0.1164440654184089	0.0147304145297127	0.06123977570615313	0.1843805443003218	-0.0929623492968902
		Sig. (2-tailed)	0.004881171836267671	0.001508087165367803	0.4067720537490582	0.8224894494193067	0.6516589446148793	0.6615157656722841	0.451610644973939	0.9243921446252078	0.692920111108487	0.2308632362285832	0.5532359061390432
		N	44	44	45	44	44	44	44	44	44	44	43
nIsoaivojenValkeaAinetilavuus_YHTEENSA	mri__t1__cross__volume__cerebral_white_matter_total_normalized	Pearson Correlation	0.037101472076474	-0.1364042991555949	0.04383579869951054	0.09453652033049187	-0.02674605834874308	0.2200539466872191	-0.1119845420324677	-0.1945265741695872	-0.1573559235873244	-0.1939347400658281	-0.0001318161686352685
		Sig. (2-tailed)	0.8110275260352744	0.3772942455235428	0.7749378393742119	0.54159610528405	0.8631729378231275	0.1511979500146922	0.4692388827050948	0.2057550175414939	0.3076786412423361	0.2071648226874067	0.9993306507973542
		N	44	44	45	44	44	44	44	44	44	44	43
nAivoselkäydinnestetilavuus_YHTEENSA	mri__t1__cross__volume__cerebrospinal_fluid_total_normalized	Pearson Correlation	0.08173870680875477	-0.1103833289989231	0.1874167044822709	-0.1692700203213305	-0.02448363807721867	-0.2580846985081449	-0.04976967518698462	0.2706667048320092	.438**	0.1220822180473668	0.01979998820092238
		Sig. (2-tailed)	0.5978683455292599	0.4756566651014305	0.2176467484470954	0.2720140326947126	0.8746504740687756	0.09075172470068033	0.7483395617687632	0.07556016431538304	0.002978961633791477	0.4298499065183796	0.8997132977941936
		N	44	44	45	44	44	44	44	44	44	44	43

			Motor Fitness	Muscular strenght		Screen time				Physical activity			
nIsoaivojen_kortikaalinen_harmaa_ainetilavuus_YHTEENSA	mri__t1__cross__volume__cerebral_cortex_total_normalized	Pearson Correlation	-0.2689534666403774	.387**	-0.1515287106410058	0.09783706829936344	0.04150180309373035	0.06033401461775385	0.06594686899798148	0.01612743865277694	0.0805827289592955	0.1956645831180077	-0.0993062460811947
		Sig. (2-tailed)	0.07750236543676702	0.009472920842357334	0.3204010266881734	0.5275088706831749	0.7890982022154168	0.6972436443505602	0.6706090033619497	0.9172452984928182	0.6030745939206755	0.2030630793381889	0.5263582684830997
		N	44	44	45	44	44	44	44	44	44	44	43
nIsoaivojen_kortikaalinen_harmaa_ainetilavuus_DX	mri__t1__cross__volume__cerebral_cortex_right_normalized	Pearson Correlation	-0.1516135581875659	.320*	-0.1776367011301865	0.1533411493385169	0.1381871035614925	0.04704912798880671	0.03005456643487175	0.01921294027723782	0.07440361422476743	0.1955564892897972	-0.08171345024954553
		Sig. (2-tailed)	0.3258866264221512	0.03420412894982843	0.2430475078550819	0.3203391577169208	0.3710311343070406	0.7616825231017876	0.8464390941867546	0.9014842042747159	0.631230339050001	0.2033177054312449	0.6024266410765553
		N	44	44	45	44	44	44	44	44	44	44	43
nIsoaivojen_kortikaalinen_harmaa_ainetilavuus_SIN	mri__t1__cross__volume__cerebral_cortex_left_normalized	Pearson Correlation	-.370*	.426**	-0.1143588235119357	0.03137219408729067	-0.06084844867237987	0.06730690807662283	0.09748892879530431	0.01337013419020728	0.08336228149214044	0.185066626553216	-0.1111328177641139
		Sig. (2-tailed)	0.01340947868487161	0.003958336312689771	0.4544517552866311	0.839792885343871	0.6947868871009969	0.6642133281821408	0.5289862395369269	0.9313566792775485	0.5905895857145385	0.2291020939912939	0.4780298116384889
		N	44	44	45	44	44	44	44	44	44	44	43
nOtsalohkotilavuus_DX	mri__t1__cross__volume__frontal_lobe_right_normalized	Pearson Correlation	-0.09146182266569416	0.2676312137704106	-0.07318451014088272	0.2430482201509853	.363*	-0.04100564599055688	0.007128195765932163	0.1683761865667756	-0.02437374927204704	0.161964718553097	0.1220992177876187
		Sig. (2-tailed)	0.5548800787288977	0.07902795060565204	0.6328188150236986	0.1118960450914085	0.01538315624277815	0.7915631292309968	0.9633720690061013	0.2745908385229228	0.8752086207660542	0.293544139030312	0.4353967193104055
		N	44	44	45	44	44	44	44	44	44	44	43
nOtsalohkotilavuus_SIN	mri__t1__cross__volume__frontal_lobe_left_normalized	Pearson Correlation	-0.1544461622693316	0.277918953558259	-0.02683334479832979	0.2238121732936978	0.2884954737065106	-0.08184880050422845	0.182830481163841	0.1754665750404432	-0.04631178611830504	0.1400517253461	0.1241446511640832
		Sig. (2-tailed)	0.3168222425359131	0.06775913847977445	0.8611035041645205	0.1441630035894597	0.05754187438946304	0.5973735377099428	0.2348762503977517	0.2545896052203508	0.765310801714489	0.3645475773883681	0.427685777617935
		N	44	44	45	44	44	44	44	44	44	44	43
nOhimolohkotilavuus_DX	mri__t1__cross__volume__temporal_lobe_right_normalized	Pearson Correlation	-0.05413156714138261	0.1883856994403507	-0.0007935376647257863	-0.1372179217349279	-0.25552912256891	0.02164741045957489	0.07157828214202586	-0.158701749525805	-0.03624871750455486	0.1918407721877995	-0.2384695108694176
		Sig. (2-tailed)	0.7270988714147092	0.2207124003682107	0.9958722325206717	0.3744281824326592	0.09410818587424986	0.8890747145888104	0.644281387473991	0.3035070614246382	0.8152944262987446	0.2122070422784765	0.1235645896645012
		N	44	44	45	44	44	44	44	44	44	44	43
nOhimolohkotilavuus_SIN	mri__t1__cross__volume__temporal_lobe_left_normalized	Pearson Correlation	-0.2814732468376138	.341*	-0.07134664211810941	-0.1799170030113187	-0.2120168611046459	-0.07346595019862369	0.104672187474103	-0.09227733641470977	0.024366004327138	0.1907139057566654	-0.1688959853015512
		Sig. (2-tailed)	0.06417646319476665	0.02333661121046078	0.6414058965996289	0.2425472715366458	0.1670824677816419	0.6355496675604924	0.4989202297432819	0.5513418115212214	0.8752479611137772	0.2149555856223039	0.2789456816635252
		N	44	44	45	44	44	44	44	44	44	44	43
nPäälakilohkotilavuus_DX	mri__t1__cross__volume__parietal_lobe_right_normalized	Pearson Correlation	-0.05666027234733848	0.1840688083354496	-.318*	0.08909478330787102	-0.00628026373679497	0.0536823683961869	-0.05086614148508511	-0.06396264984306602	0.1336432705508993	0.114176795550853	-0.1469321869179572
		Sig. (2-tailed)	0.7148758512550407	0.2316665037596777	0.03334249609716957	0.5652100943609935	0.967726686114243	0.7292772866465078	0.7429821535125231	0.6799812294605585	0.3871169635099441	0.4605275605716739	0.3471027808742289
		N	44	44	45	44	44	44	44	44	44	44	43
nPäälakilohkotilavuus_SIN	mri__t1__cross__volume__parietal_lobe_left_normalized	Pearson Correlation	-.460**	.471**	-0.2261310203464621	-0.1440109446715846	-.391**	0.1956518879330387	-0.05668439669785477	0.00329792476558734	0.2076163313171487	-0.0116671498738732	-0.1052631610101051
		Sig. (2-tailed)	0.001696608703871802	0.001245647487012148	0.1352617726595212	0.3510089123559368	0.00867100027293933	0.203092972543073	0.7147595742967529	0.9830492421718948	0.1762752010760767	0.9400829181630908	0.5017193282550826
		N	44	44	45	44	44	44	44	44	44	44	43
nTakaraivolohkotilavuus_DX	mri__t1__cross__volume__occipital_lobe_right_normalized	Pearson Correlation	-0.2308301491302169	0.113799665764312	-0.0666524723424297	0.09541659416283978	0.08201936718243305	0.1692493832691281	0.03201118422459487	0.003113294389706063	0.2205252471297882	-0.05010761950021359	-0.07136098202831412
		Sig. (2-tailed)	0.131678341566756	0.4620199168454591	0.6635473917064546	0.537822194703729	0.5966072932039421	0.2720733460825915	0.8365737031004346	0.9839980838166634	0.1503021674136094	0.7466870810137946	0.649298612952345
		N	44	44	45	44	44	44	44	44	44	44	43
nTakaraivolohkotilavuus_SIN	mri__t1__cross__volume__occipital_lobe_left_normalized	Pearson Correlation	-0.1036409333656332	0.01599315172426623	0.03187503945280847	0.04904812850065005	-0.0681205722881459	0.2366791431719312	-0.06859236622269359	-0.204014189210042	0.06733918889784261	0.1362826536862889	-0.3006433551799001
		Sig. (2-tailed)	0.5031821036717139	0.9179320077862608	0.8353383304914944	0.751871504851122	0.6603982188785545	0.1219053958899756	0.6581899537216088	0.1840662091915592	0.6640618091845218	0.3777238703967202	0.05011968120675386
		N	44	44	45	44	44	44	44	44	44	44	43
nSisempi_ohimolohkotilavuus_DX	mri__t1__cross__volume__medial_temporal_lobe_right_normalized	Pearson Correlation	0.1745212416455607	-0.1816826202051257	-0.06730855459763455	-0.1708579623760673	-0.2409325942976596	-0.1351850822295097	0.1465183409439308	-0.04883202374744703	-0.2203188259946698	-0.04247629886440246	-0.05361340952734756
		Sig. (2-tailed)	0.2571982827975652	0.2378784910972136	0.6604351363713309	0.2674756699755075	0.1151490317917204	0.3816133709974224	0.3425958820709702	0.7529303083193795	0.1506940229258741	0.784262809595833	0.7327627893851245
		N	44	44	45	44	44	44	44	44	44	44	43
nSisempi_ohimolohkotilavuus_SIN	mri__t1__cross__volume__medial_temporal_lobe_left_normalized	Pearson Correlation	-0.07835326714452824	-0.07335712898855919	-0.0634843771670289	-0.2273901310350565	-.323*	-0.117919368207266	0.1694694726185061	-0.05869708033677171	-0.068628703335278813	0.1842640834906433	-0.1108204204720612
		Sig. (2-tailed)	0.6131706188765679	0.6360517284024282	0.6786539990292564	0.1376930319499094	0.03221086673421419	0.4458593843894836	0.2714412223040105	0.7050813421490375	0.6580199940271286	0.2311631038312147	0.4792756001670635
		N	44	44	45	44	44	44	44	44	44	44	43
nIsoaivojen_harmaa_ja_valkean_ainetilavuus_YHTEENSA	mri__t1__cross__volume__cerebral_gray_and_white_matter_total_normalized	Pearson Correlation	-0.2522911404529546	0.2684372459751492	-0.1018352559560041	0.1571641833269912	-0.009024850521497858	0.2545374723759901	-0.02779638179389444	-0.142411969585342	-0.07357147557448698	0.01982674333122854	-0.07931123686835295
		Sig. (2-tailed)	0.09849772091918897	0.07809519422574546	0.505639955963129	0.3082759295714454	0.9536356568411802	0.09543613615925314	0.8578538167040847	0.3564392339309943	0.6350629666166965	0.8983530931903707	0.6131719149645506
		N	44	44	45	44	44	44	44	44	44	44	43
nHippokampustilavuus_YHTEENSA	mri__t1__cross__volume__hippocampus_total_normalized	Pearson Correlation	0.1487259395314591	-0.2902064404028974	0.03445090232812584	-0.02173630424843333	-0.07095068424901714	-0.01939943341953796	0.09137016441109636	0.03176545922699353	-0.2355348335702755	0.05744749415148254	0.1440409114708649
		Sig. (2-tailed)	0.3352925509214375	0.05601165540060444	0.8222383460865434	0.8886220637967115	0.6471950303857975	0.9005327134138981	0.5552784238216321	0.8378113372682431	0.1237730507768247	0.7110848264011791	0.3567765534517471
		N	44	44	45	44	44	44	44	44	44	44	43

			Motor Fitness	Muscular strenght		Screen time				Physical activity			
nAccumbensAreatilavuus_YHTEENSA	mri__t1__cross__volume__accumbens_area_total_normalized	Pearson Correlation	-0.143002983604086	0.1371521581373009	-.297*	0.09465000198213205	0.001378094460017602	-0.0007813402834149011	0.05456104505763162	0.1475704129137407	-0.205249810916606	-0.07536779788193657	.313*
		Sig. (2-tailed)	0.3544261601383274	0.3746593577774655	0.04760413450122101	0.5411087615801291	0.9929164110328745	0.995983772433398	0.7250180794303199	0.3391032059071679	0.1813665039516655	0.6268014716542922	0.04118532080657146
		N	44	44	45	44	44	44	44	44	44	44	43
nMantelitumaketilavuus_YHTEENSA	mri__t1__cross__volume__amygdala_total_normalized	Pearson Correlation	0.106111020581451	-0.08711058568233603	-0.02384410116715277	-0.1118827139956581	-0.2200536120967161	0.01713388892203126	0.008482916890150162	-0.04330520418262049	-0.1603969004583763	-0.04874660503704462	-0.003356149620611017
		Sig. (2-tailed)	0.4930049941709399	0.5739373792153103	0.8764491918570264	0.4696456378254416	0.1511985873472796	0.9121004949871417	0.9564170355542163	0.7801561291963335	0.2983044980825348	0.7533489402546101	0.9829590566280426
		N	44	44	45	44	44	44	44	44	44	44	43
nCaudatetilavuus_YHTEENSA	mri__t1__cross__volume__caudate_total_normalized	Pearson Correlation	-.387**	.464**	-.379*	-0.07920591645405098	-0.2511975359494716	-0.009585591740977149	0.05036626184609055	0.2169413138075536	-0.02593371012156843	-0.02118542043891435	0.2457761481019255
		Sig. (2-tailed)	0.00935926362532579	0.00153066240059405	0.01033741844975116	0.6093009361895203	0.1000153112630559	0.9507583246763915	0.7454231285891482	0.1572126531098153	0.86729100442049	0.8914277447740422	0.11214002809835 4
		N	44	44	45	44	44	44	44	44	44	44	43
nPikkuaivojenHarmaaAinetilavuus_YHTEENSA	mri__t1__cross__volume__cerebellum_exterior_total_normalized	Pearson Correlation	-0.07969928672543955	0.01417780024664775	-0.04556164469186169	-0.2449409451453248	-0.1259140489745437	-.304*	.355*	0.110849800392296	-0.1572891589797394	0.06418509496551987	0.236569827770301
		Sig. (2-tailed)	0.6070666059998976	0.9272208446523957	0.7663210852531103	0.1090454259964257	0.4154023552114251	0.0451459029844469	0.0180828321029608 6	0.473782238418023	0.307886535236249	0.6789281291546205	0.1266733931772286
		N	44	44	45	44	44	44	44	44	44	44	43
nPikkuaivojenValkeaAinetilavuus_YHTEENSA	mri__t1__cross__volume__cerebellum_white_matter_total_normalized	Pearson Correlation	-0.1121835665516125	0.1316521965235667	-0.2262128320387701	-0.04600879999227078	-0.06618312492757107	-0.00948990914902292 9	0.2293792018395692	-0.005712366733753274	-0.09743314201626951	-0.01352115426951511	0.1458141194550421
		Sig. (2-tailed)	0.4684444157960006	0.3942928225342178	0.1351167353094823	0.7668031670188329	0.6694963259240796	0.9512492581100361	0.134190854686588	0.970643689240223	0.5292231654463204	0.9305832081289771	0.350824438001381
		N	44	44	45	44	44	44	44	44	44	44	43
nTemporaalisarvi_sivuaivokammiotilavuus_YHTEENSA	mri__t1__cross__volume__inferior_lateral_ventricle_total_normalized	Pearson Correlation	-0.199801149688747	0.1102893253125137	.339*	-0.03976433826714464	-0.1762546603288037	0.06675627841661559	-0.2017133756268137	-0.04013574898653968	0.1491143135332977	-0.07018754835748725	-0.1295582105309337
		Sig. (2-tailed)	0.1934865911128869	0.4760348749885536	0.02288166044581643	0.7977387480054394	0.2524284625332354	0.6667998943951009	0.1891694213567609	0.795889648006392	0.3340177770489929	0.6507449425178876	0.4076513261040597
		N	44	44	45	44	44	44	44	44	44	44	43
nSivuaivokammiotilavuus_YHTEENSA	mri__t1__cross__volume__lateral_ventricle_total_normalized	Pearson Correlation	0.08204260206498709	-0.1160385732405883	0.1644938311790098	-0.1710184703028489	-0.001196251892203753	-0.2842723607932269	-0.04326388954851873	0.2741749344971554	.453**	0.1525492105532195	0.01351325625550728
		Sig. (2-tailed)	0.596502947459743	0.453198453140782	0.2802388965798983	0.2670197444681895	0.9938510868475106	0.06146245921429398	0.7803606771055409	0.0717026445027318	0.002021161858384243	0.3228747239426504	0.9314624055901788
		N	44	44	45	44	44	44	44	44	44	44	43
nPallidumtilavuus_YHTEENSA	mri__t1__cross__volume__pallidum_total_normalized	Pearson Correlation	-0.2672550154453974	0.1382716892183808	-0.0860431769678388	0.03597243427583924	-0.05970377793733657	0.01389103870169458	0.1083191603402452	-0.1683623711906673	-0.2790984396639993	0.00735467036155213 4	0.01361210350282152
		Sig. (2-tailed)	0.0794662728966372	0.3707355332017028	0.5741182247611197	0.8166779951374863	0.7002576072281542	0.928689059909732	0.4839979732278883	0.2746307920976214	0.06655307315015363	0.9622091650854954	0.930962266556211
		N	44	44	45	44	44	44	44	44	44	44	43
nPutamentilavuus_YHTEENSA	mri__t1__cross__volume__putamen_total_normalized	Pearson Correlation	-0.07291483138625632	0.07103136276260716	-0.01615742207625082	.353*	0.058044158012477	.329*	0.04367728443645959	-0.1162358693982411	-0.1812679897532221	-0.03848487529803574	0.07038008536796313
		Sig. (2-tailed)	0.6380939832218162	0.6468201846519782	0.9161028202689466	0.01862737850793233	0.7082160468490503	0.02945906687590746	0.7783146298128991	0.4524255106000676	0.2389693530662551	0.8041169825770604	0.6538136805422488
		N	44	44	45	44	44	44	44	44	44	44	43
nTalamustilavuus_YHTEENSA	mri__t1__cross__volume__thalamus_proper_total_normalized	Pearson Correlation	-0.2849279649168419	0.1432504309133971	0.02019638564119338	-0.01691028841671349	0.09646455443433645	-0.05854402077139287	0.08061689714770039	0.1348683155186238	0.07891122194301262	0.1335314325931858	0.07463928520112807
		Sig. (2-tailed)	0.0608402183485398	0.3535853805563169	0.8952355574763995	0.913243203585296	0.5333450219651988	0.7058157562419823	0.6029204269520855	0.3827402958976173	0.6106372007392817	0.387517982181502	0.6342991281078963
		N	44	44	45	44	44	44	44	44	44	44	43
nVentral_diencephalontilavuus_YHTEENSA	mri__t1__cross__volume__ventral_diencephalon_total_normalized	Pearson Correlation	-0.140960611268369	-0.01336316095490066	0.1391027660144092	-0.162399744798647	-0.01025119323627964	-0.2356840316047259	0.1348377858884321	-0.08616545499521792	-0.2200915952344363	-0.01204492916733324	0.04453151882800157
		Sig. (2-tailed)	0.3614121470801732	0.931392395223913	0.3621306590107817	0.2922320258874635	0.9473437287188923	0.1235283284055113	0.3828490114335714	0.5781159501719408	0.1511262491420939	0.9381465100744232	0.7767563405923227
		N	44	44	45	44	44	44	44	44	44	44	43
nVesseltilavuus_YHTEENSA	mri__t1__cross__volume__vessel_total_normalized	Pearson Correlation	-0.07219025459529112	0.1824477503808669	-0.2184028593495231	-0.05499231028600061	-0.1490030288546075	0.1927194239274651	-.335*	0.02591648618793944	0.2349355893430224	-0.04645975775721036	-0.08790106834639874
		Sig. (2-tailed)	0.6414453488537536	0.2358743987178819	0.1494917558583991	0.722930595530191	0.3343827424295978	0.2100809678002144	0.02640399963594153	0.8673783563224268	0.124759636894438	0.7645822686063914	0.5751332116330639
		N	44	44	45	44	44	44	44	44	44	44	43
nAnterior_cingulate_gyrustilavuus_YHTEENSA	mri__t1__cross__volume__anterior_cingulate_gyrus_total_normalized	Pearson Correlation	-0.1148493478226937	0.08339722120725199	0.09899307037716809	-0.08598863305551038	-0.05857769820405676	0.1077424608209976	-0.158278422802326	0.1120928397378329	0.08341094600558252	-0.02433954228349318	0.07089111290682858
		Sig. (2-tailed)	0.4578726361417511	0.5904333780120362	0.517650794682826	0.5788992362477509	0.7056541416830854	0.486342002975636	0.3048152992904651	0.4688064904293504	0.5903720225032963	0.8753823769644188	0.6514598853799216
		N	44	44	45	44	44	44	44	44	44	44	43
nAnterior_insulatilavuus_YHTEENSA	mri__t1__cross__volume__anterior_insula_total_normalized	Pearson Correlation	-0.2845501693797073	0.2695950689913647	0.1767276077005808	-0.002544670991847328	0.0243185406877965	-0.2446325192547418	.348*	0.102878626105756	-0.2565294945436331	0.1719714152074731	0.1381057862272571
		Sig. (2-tailed)	0.06119817106310441	0.07677047852826939	0.2455066258135795	0.986920451402054	0.8754890585441328	0.1095061300491088	0.0204914085695948	0.5063443694451938	0.0927830674854279	0.264323499197641	0.3771406751377849
		N	44	44	45	44	44	44	44	44	44	44	43
nAnterior_orbital_gyrustilavuus_YHTEENSA	mri__t1__cross__volume__anterior_orbital_gyrus_total_normalized	Pearson Correlation	0.0877694944821594	-0.1481042534005462	0.1149957155663468	-0.1688510433313423	-0.1780843170153918	-0.1243417219980812	0.2100141475836488	0.2469832227602079	0.1379503606566888	.311*	0.0817909762890477
		Sig. (2-tailed)	0.5710324233706561	0.337339409970433	0.4519262062188687	0.2732198951695385	0.247458675965573	0.4212969134633913	0.1712221259716145	0.1060320070822298	0.3718592277948103	0.03962880292222118	0.6020812258150254
		N	44	44	45	44	44	44	44	44	44	44	43
nAngular_gyrustilavuus_YHTEENSA	mri__t1__cross__volume__angular_gyrus_total_normalized	Pearson Correlation	-0.1252687488807769	0.1190695159401763	-0.01693752950998613	-0.2496165276909704	-0.2129710891694971	-0.1422317543825165	-0.08797089004982758	-0.01533701800712854	0.2691549669347251	-0.01193140394196617	-0.2279465965531562
		Sig. (2-tailed)	0.4178158435634064	0.4414037376399235	0.9120676174871665	0.1022409527251508	0.1651357540732713	0.3570544502651032	0.5701458712683255	0.9212881486213368	0.07727192300882976	0.93872837721975	0.14152574589423

			Motor Fitness	Muscular strenght		Screen time				Physical activity			
		N	44	44	45	44	44	44	44	44	44	44	43
nCalcarine_cortextilavuus_YHTEENSA	mri__t1__cross__volume__calcarine_cortex_total_normalized	Pearson Correlation	-0.2673572466047107	0.1159611490082149	0.2007262292956644	-0.01251001923270482	-0.1264029897768763	.304*	-0.07942969071374842	-0.1629183803241649	-0.02318539745936322	-0.150525284033341	-0.1741779390599117
		Sig. (2-tailed)	0.0793469716357096	0.4535019723951299	0.1861268788828746	0.9357630526778654	0.4135789644919246	0.0447295363680499	0.6082870909034281	0.2906727023235162	0.8812482588363288	0.3294118929987713	0.263964390094623
		N	44	44	45	44	44	44	44	44	44	44	43
nCentral_operculumtilavuus_YHTEENSA	mri__t1__cross__volume__central_operculum_total_normalized	Pearson Correlation	-0.1640625142658206	0.2303606986855862	-0.2803887297406993	0.168277140048501	0.1245141050441263	0.2075542896957883	-0.131330325434457	-0.2504634492553023	-0.1846848815407626	-0.03208781919547415	-0.142097711877938
		Sig. (2-tailed)	0.287251873745393	0.1324873834882046	0.06209835808468248	0.2748773621291757	0.4206483612809087	0.1764073518739686	0.3954600882884572	0.1010440353517814	0.2300808755006361	0.8361877981215844	0.3633691576722682
		N	44	44	45	44	44	44	44	44	44	44	43
nCuneustilavuus_YHTEENSA	mri__t1__cross__volume__cuneus_total_normalized	Pearson Correlation	0.1385261937160958	-0.1179230674521195	-0.1196037242525885	.330*	0.08580756987088106	.437**	0.03160007324456248	-0.1921709985250394	0.07194897851633948	0.1731007032475476	-0.1849233825895235
		Sig. (2-tailed)	0.369846967683357	0.4458450141358363	0.4338833155143872	0.02880364122805662	0.5797018076813782	0.003047216789803401	0.8386445488309684	0.2114062432246734	0.6425628854599492	0.2611518112983913	0.2351631490464894
		N	44	44	45	44	44	44	44	44	44	44	43
nEntorinaalinen_aluetilavuus_YHTEENSA	mri__t1__cross__volume__entorhinal_area_total_normalized	Pearson Correlation	0.0401745031123658	-0.0573556137331331	0.05969560150960449	-0.2447543236649658	-.379*	-0.05656536136120965	0.08437350950429666	-0.066955141435844	0.0263851084001983	0.04466872381948918	-0.18307526883184
		Sig. (2-tailed)	0.7956967707232783	0.7115269422650071	0.6968846841959696	0.1093240106854495	0.01111094070788599	0.7153333746074737	0.5860760434391684	0.6658652989771763	0.8650022789496016	0.7734136779738507	0.2399575242917749
		N	44	44	45	44	44	44	44	44	44	44	43
nFrontal_operculumtilavuus_YHTEENSA	mri__t1__cross__volume__frontal_operculum_total_normalized	Pearson Correlation	-0.276128010475146	.363*	0.05188898715805408	0.2441080836262689	0.1653448388681133	0.1080105628649907	0.1363497046108654	-0.03471392909735101	-0.2320699251322199	-0.06881465725762508	0.02024268742446368
		Sig. (2-tailed)	0.0696234933264767	0.01529525750371471	0.7349751272823608	0.1102928958558394	0.2834491196999914	0.4852515510204849	0.3774870254496299	0.8229872358858984	0.1295594804820975	0.6571504984196606	0.8974828229501246
		N	44	44	45	44	44	44	44	44	44	44	43
nFrontal_poletilavuus_YHTEENSA	mri__t1__cross__volume__frontal_pole_total_normalized	Pearson Correlation	0.0310757191212588	-0.06737838393023501	0.06008260687698044	0.1496592148294484	.330*	-0.1927547400337982	-0.07013946886395929	-0.02982210632560441	0.09694564657207427	-0.01687541428111283	-0.1248543898442811
		Sig. (2-tailed)	0.8412873839650689	0.6638778542070346	0.695014273240025	0.3322343135144541	0.02886752755031068	0.2099958251466332	0.6509688528978346	0.84761274681545	0.5312957649029053	0.9134214432198979	0.4250281883290852
		N	44	44	45	44	44	44	44	44	44	44	43
nFusiform_gyrustilavuus_YHTEENSA	mri__t1__cross__volume__fusiform_gyrus_total_normalized	Pearson Correlation	-0.2797650597288658	0.2523621267557336	0.1932792941713534	-.299*	-0.2739974991968353	-0.03198131891521009	-0.1530284197095243	0.2170994141062046	0.1541563472460209	-0.01349185872741725	0.07145291250247524
		Sig. (2-tailed)	0.06587900479210168	0.09839982942066142	0.2033352751189951	0.04835053315712574	0.07189393665053824	0.8367241038704678	0.3213389275172562	0.1569029975984209	0.3177422637342924	0.9307332445612708	0.6488760889042424
		N	44	44	45	44	44	44	44	44	44	44	43
nGyrus_rectustilavuus_YHTEENSA	mri__t1__cross__volume__gyrus_rectus_total_normalized	Pearson Correlation	-0.1625026628651799	.307*	-.295*	0.01187073458899732	0.05017778165798073	-0.2553075220204522	.297*	0.06473768618148677	-0.09680227031610544	0.01856966831479222	0.05079830688689674
		Sig. (2-tailed)	0.291922163765945	0.04241697861538087	0.04904730610023139	0.9390393475165607	0.7463441423523715	0.09440369229021507	0.04994722629955464	0.6763146668779544	0.5319060867431064	0.9047672263743571	0.746316576186705
		N	44	44	45	44	44	44	44	44	44	44	43
nInferior_occipital_gyrustilavuus_YHTEENSA	mri__t1__cross__volume__inferior_occipital_gyrus_total_normalized	Pearson Correlation	-0.136677683432303	0.09182463109945045	-0.148997020608352	-0.1120875888108685	-0.01818885936016373	-0.01021014474285208	-0.1230559300879595	0.136839130805008	0.2149776257026446	0.04189705560839106	0.08211653764787985
		Sig. (2-tailed)	0.3763297727767219	0.5533046424552829	0.3286468569966915	0.4688274505160672	0.9067114774459256	0.9475542848613879	0.4261522179304575	0.3757608917020265	0.1610960086061043	0.7871360266612404	0.6006316350231001
		N	44	44	45	44	44	44	44	44	44	44	43
nInferior_temporal_gyrustilavuus_YHTEENSA	mri__t1__cross__volume__inferior_temporal_gyrus_total_normalized	Pearson Correlation	0.001251165528857627	0.2042276056156457	0.1630716769917272	-0.2088690933358712	-0.211082427730437	-0.02109787212040313	0.00978359956356189	-0.1257284018730113	0.1367354837208284	0.1491340249855501	-0.2230997339804035
		Sig. (2-tailed)	0.9935688283266294	0.1835978796086451	0.2844754492709739	0.1736219742679449	0.1690048514977469	0.8918737546498952	0.9497424328071896	0.416095879733834	0.3761260470123016	0.3339531578719677	0.1504197258569447
		N	44	44	45	44	44	44	44	44	44	44	43
nLingual_gyrustilavuus_YHTEENSA	mri__t1__cross__volume__lingual_gyrus_total_normalized	Pearson Correlation	-0.1975757042027881	0.1238781341254209	0.02879035297713815	-0.003286939058851945	0.0725679344926588	-0.01017073223568666	0.02994457805370831	0.09392156535139567	0.0774999871811825	-0.05939659816125184	0.03412077777795827
		Sig. (2-tailed)	0.1985981512099701	0.4230438599878086	0.8510839066260659	0.9831056986405096	0.6396975921189382	0.9477564526184153	0.8469943665630149	0.5442406784140802	0.6170535941434682	0.7017282783395808	0.8280408386071986
		N	44	44	45	44	44	44	44	44	44	44	43
nLateral_orbital_gyrustilavuus_YHTEENSA	mri__t1__cross__volume__lateral_orbital_gyrus_total_normalized	Pearson Correlation	0.2188363274142647	-0.03771996342517071	-0.1655499046699022	0.08892383052080133	0.2898783262938021	-0.215316498054274	-0.0288400241821675	0.1283082836680554	-0.0961610344268738	0.1931152200618071	0.1790332086484786
		Sig. (2-tailed)	0.1535303681881643	0.8079361753319112	0.2771197776475786	0.5659595857798928	0.05630254823075224	0.1604209232838879	0.8525746834892334	0.4065173271545059	0.5346398719844017	0.2091281301854739	0.2506733851969786
		N	44	44	45	44	44	44	44	44	44	44	43
nMiddle_cingulate_gyrustilavuus_YHTEENSA	mri__t1__cross__volume__middle_cingulate_gyrus_total_normalized	Pearson Correlation	-0.07320601497864225	0.1178362380857772	0.121308940362671	-0.0481622051533628	-0.01870648621954376	-0.1102750561758473	0.1045465721363204	0.1749320746537945	0.1036322804254076	0.1191805727370208	0.1620688633433274
		Sig. (2-tailed)	0.636749179911236	0.4461823824692905	0.4273097492234643	0.7562149178814463	0.9040688251758547	0.4760922986276578	0.4994383706417125	0.2560623881869687	0.5032179420007717	0.4409748114638943	0.2991189797887622
		N	44	44	45	44	44	44	44	44	44	44	43
nMedial_frontal_cortextilavuus_YHTEENSA	mri__t1__cross__volume__medial_frontal_cortex_total_normalized	Pearson Correlation	0.02597390517077936	0.1153718900599841	-0.160994456386106	0.09019064980398565	-0.1710284126689437	0.2317853253446589	0.0541056182196954	0.01526480843259602	-0.2697588256238085	-0.1230293574497192	0.2359185572065299
		Sig. (2-tailed)	0.8670871596190649	0.4558156097362716	0.2907381604339123	0.5604165501555817	0.2669915199595413	0.1300436096993955	0.7272246545533924	0.9216575860443508	0.07658455100600496	0.4262528900002229	0.1277525693451496
		N	44	44	45	44	44	44	44	44	44	44	43
nMiddle_frontal_gyrustilavuus_YHTEENSA	mri__t1__cross__volume__middle_frontal_gyrus_total_normalized	Pearson Correlation	0.04937379530047467	-0.01144681383169829	0.07792090388434653	0.1637203316195963	0.1872050082384698	0.05822279896457634	0.002264699947321039	0.09254072521768868	0.01024091017831347	0.1976375100147766	0.0702751597924191
		Sig. (2-tailed)	0.7502767534663343	0.9412124674233907	0.6109100278156555	0.2882722040274317	0.2236721735595163	0.7073579087564602	0.9883593914472171	0.5502013356767085	0.9473964748053363	0.1984549193183454	0.6542973797300262
		N	44	44	45	44	44	44	44	44	44	44	43
nMiddle_occipital_gyrustilavuus_YHTEENSA	mri__t1__cross__volume__middle_occipital_gyrus_total_normalized	Pearson Correlation	-0.07820110732662616	0.02511690833852032	-0.1949176312036631	-0.1211884735049676	-0.02297010514496267	-0.1218849314142034	-0.1320533702391863	-0.1408691559535005	.318*	0.2422542658881283	-.342*

			Motor Fitness	Muscular strenght		Screen time				Physical activity			
		Sig. (2-tailed)	0.6138622824473832	0.8714351500564715	0.1994568320803275	0.4332596662168475	0.8823431757690511	0.4306012876841028	0.392840782121114	0.3617256663708707	0.03531510668185987	0.1131085388952046	0.0248103317027865
		N	44	44	45	44	44	44	44	44	44	44	43
nMedial_orbital_gyrustilavuus_YHTEENSA	mri__t1__cross__volume__medial_orbital_gyrus_total_normalized	Pearson Correlation	0.2905126376807193	-0.07445568006630025	-0.22046195187649	0.08914302312954524	0.187302561663465	-0.1449952424349301	0.06893273622287954	0.0034538088996746	-0.1889014988791214	-0.05376759706680543	0.128261253357365
		Sig. (2-tailed)	0.05574128229113618	0.6309908524995375	0.1455970224372247	0.5649986844890141	0.2234265936936151	0.3476913849933081	0.6565986089644698	0.9822481494746124	0.2194279206107026	0.7288638027513938	0.4124013487868643
		N	44	44	45	44	44	44	44	44	44	44	43
nPostcentral_gyrus_medial_segmenttilavuus_YHTEENSA	mri__t1__cross__volume__postcentral_gyrus_medial_segment_total_normalized	Pearson Correlation	-0.1407253099689582	0.1377824115827071	-0.1834955196055167	0.05409088311058217	-0.03184079535632699	0.1516133547581898	-0.1662960767457013	-.337*	0.1320745252688526	0.01461903939299684	-.357*
		Sig. (2-tailed)	0.362222310399245	0.3724473592370214	0.2276001342354936	0.7272960837302536	0.837431853267065	0.3258872831774395	0.2806495377367224	0.02537893489024105	0.3927642987064737	0.9249621742394701	0.0189272019228683
		N	44	44	45	44	44	44	44	44	44	44	43
nPrecentral_gyrus_medial_segmenttilavuus_YHTEENSA	mri__t1__cross__volume__precentral_gyrus_medial_segment_total_normalized	Pearson Correlation	-0.1797810084493735	0.2835716758345256	0.1636744575694049	-0.02269377053754526	-0.1432481736994521	0.06617679379922009	-0.08108973403361357	0.1645533434786443	0.2303432819922165	0.1846151412514788	-0.05388568456528057
		Sig. (2-tailed)	0.2429094364246536	0.06213308908933448	0.282674708589312	0.8837488541852235	0.3535930446503543	0.6695261340782142	0.600788743215301	0.2857924110092671	0.1325174702956059	0.2302599963989208	0.7314560464167863
		N	44	44	45	44	44	44	44	44	44	44	43
nSuperior_frontal_gyrus_medial_segmenttilavuus_YHTEENSA	mri__t1__cross__volume__superior_frontal_gyrus_medial_segment_total_normalized	Pearson Correlation	-0.04231092375694177	0.1188828403605432	-0.2039578880213969	0.0568058748140173	0.01565215413162294	-0.1447273096104387	.361*	0.01383536707856768	-.346*	0.02434049457828468	0.04454063346915037
		Sig. (2-tailed)	0.7850828315907477	0.4421252390895445	0.178991822164958	0.7141741589251698	0.9196760470351529	0.3485925297593804	0.01606838930993294	0.9289741251459604	0.0212256825328997	0.8753775396545245	0.7767118150128887
		N	44	44	45	44	44	44	44	44	44	44	43
nMiddle_temporal_gyrustilavuus_YHTEENSA	mri__t1__cross__volume__middle_temporal_gyrus_total_normalized	Pearson Correlation	-0.1548269968698686	0.14343644857225	-0.1191214985529571	-0.01748071731481573	-0.1305708773685723	-0.04331408461768047	0.1628535495612168	-0.1904654305288314	-0.03303659673848748	0.02612003143992989	-0.2614129686750714
		Sig. (2-tailed)	0.31561584197517	0.352954128190195	0.4357524567406756	0.9103283786192725	0.3982221907594733	0.780112164258317	0.2908673261667416	0.2155649541854172	0.831413284648048	0.8663461680763561	0.09040356598422687
		N	44	44	45	44	44	44	44	44	44	44	43
nOccipital_poletilavuus_YHTEENSA	mri__t1__cross__volume__occipital_pole_total_normalized	Pearson Correlation	0.03967785144328985	0.00765568972043658	0.01016749427653092	.350*	0.2215135413699998	0.2629545307508325	0.004398316184287153	-0.1334422203335951	-0.1618311380359288	0.004365734899826465	-0.09868050415555933
		Sig. (2-tailed)	0.7981694874953489	0.9606636083384666	0.9471485666478593	0.01992000469612751	0.1484364280100418	0.08461337341913938	0.9773946985127222	0.3878380465557736	0.2939478037707867	0.9775621084663388	0.528980547121801
		N	44	44	45	44	44	44	44	44	44	44	43
nOccipital_fusiform_gyrustilavuus_YHTEENSA	mri__t1__cross__volume__occipital_fusiform_gyrus_total_normalized	Pearson Correlation	-0.07219422047901161	-0.1739618288625528	-0.02275076981194764	-0.101296793891522	-0.1351198681147885	0.0103269552683756	0.1214995013370461	-0.1579520739119069	0.01956123320556633	0.08411503134565461	-0.143179890476386
		Sig. (2-tailed)	0.64142698630132	0.2587503901068315	0.8820734709800493	0.5129382441132693	0.3818452152915912	0.9469551205778897	0.4320713446853539	0.3058262976957233	0.8997073199747803	0.5872282737314868	0.3596886958721965
		N	44	44	45	44	44	44	44	44	44	44	43
nOpercular_part_of_the_inferior_frontal_gyrustilavuus_YHTEENSA	mri__t1__cross__volume__opercular_part_of_the_inferior_frontal_gyrus_total_normalized	Pearson Correlation	0.02761799208718913	0.1002047563847778	-0.2733459939238544	0.1698516322238826	0.2634730085608207	-0.1308411579679911	-0.00792301659806047	0.236662406708038	0.05406357642428716	0.003894043628504447	0.1980769465494384
		Sig. (2-tailed)	0.858756798547302	0.5175154276686379	0.0692422846710556	0.2703459176339867	0.08397939064441831	0.3972379026136608	0.9592911618670712	0.1219325579889431	0.7274284602927069	0.9799858672480687	0.202924775112786
		N	44	44	45	44	44	44	44	44	44	44	43
nOrbital_part_of_the_inferior_frontal_gyrustilavuus_YHTEENSA	mri__t1__cross__volume__orbital_part_of_the_inferior_frontal_gyrus_total_normalized	Pearson Correlation	-0.1494902930086917	0.1000350068484198	0.03124665868697865	-0.2683831863645585	-0.2052774159149452	-0.1749213952978371	-0.2580487291197021	0.1964001547664679	0.1285217235570313	0.04855520867165657	0.0491812461200687
		Sig. (2-tailed)	0.3327865598649984	0.5182287434827432	0.8385409071162689	0.07815748160416253	0.181306515113737	0.2560918724954083	0.09079831370965827	0.2013363073289376	0.405730594112065	0.7542872158758541	0.7541367983457156
		N	44	44	45	44	44	44	44	44	44	44	43
nPosterior_cingulate_gyrustilavuus_YHTEENSA	mri__t1__cross__volume__posterior_cingulate_gyrus_total_normalized	Pearson Correlation	0.06352987369816782	0.05428539640945956	0.01950375813835924	-0.01633992236610045	-0.1259057323853148	-0.006901502294435335	0.01491991488052908	-0.0418718896061125	-0.2630312413923277	-0.08111392335315722	-0.01780720168621188
		Sig. (2-tailed)	0.6820318027508903	0.7263533549419532	0.8988094395421892	0.9161588324309446	0.4154334095241458	0.9645361720206227	0.923422348120343	0.7872609210651202	0.0845193389293842	0.6006797791494723	0.9097628626492155
		N	44	44	45	44	44	44	44	44	44	44	43
nPrecuneustilavuus_YHTEENSA	mri__t1__cross__volume__precuneus_total_normalized	Pearson Correlation	-0.1618295296582548	0.2243617997936754	-0.2500968378378693	0.06245032494887266	-0.07753096033362952	0.06926985875838178	0.1789881730119033	-0.08283296101684236	0.0378701939996629	0.06519290491217196	-0.1029760155905692
		Sig. (2-tailed)	0.2939526662853098	0.1431547864049686	0.09753184209434175	0.687156749892821	0.6169124647786637	0.6550239256937609	0.2450281164940027	0.5929582986796158	0.8071857252806358	0.6741645425289734	0.5111093308302599
		N	44	44	45	44	44	44	44	44	44	44	43
nParahippocampal_gyrustilavuus_YHTEENSA	mri__t1__cross__volume__parahippocampal_gyrus_total_normalized	Pearson Correlation	-0.06313156947890025	0.04444207656953082	-0.219094298753714	-0.2199583622720973	-0.1901905177889211	-0.2614101154263932	0.2056091004705879	-0.09803170471106638	-0.06563642175951137	0.07769996879946994	-0.2128486949866837
		Sig. (2-tailed)	0.6839210440436952	0.7745332964574705	0.1481754544204388	0.1513801007411183	0.2162405551357255	0.08652397575536709	0.1805868359023957	0.5266837941464707	0.67207215310368	0.6161426169793007	0.1705695648837371
		N	44	44	45	44	44	44	44	44	44	44	43
nPosterior_insulatilavuus_YHTEENSA	mri__t1__cross__volume__posterior_insula_total_normalized	Pearson Correlation	-0.2025447570997772	0.2553330717472999	-0.2226282229781183	0.1654437674656772	-0.07795684779592085	0.2309367214659177	0.02831877916737868	-0.001028693796536474	-0.003709657851699511	0.1742382747717936	0.04154998607893992
		Sig. (2-tailed)	0.1873139452217835	0.09436958497910455	0.1415809933493067	0.2831571172946866	0.6149732906437979	0.1314951918708416	0.8552105548370142	0.9947123473095578	0.9809333726832762	0.2579826027351138	0.791358426421955
		N	44	44	45	44	44	44	44	44	44	44	43
nParietal_operculumtilavuus_YHTEENSA	mri__t1__cross__volume__parietal_operculum_total_normalized	Pearson Correlation	-0.1133985473257975	0.2537300202989548	-0.1905569005884195	0.06368267390916231	-0.1087510031713125	0.2021467832689549	-0.062943352327726	-0.2643260014204628	-0.03196188788176297	0.2410712825763973	-0.1596391321527409
		Sig. (2-tailed)	0.4636100504306665	0.09652806335753021	0.2098965775248105	0.6813075477247919	0.482246596876286	0.1882005202665407	0.6848144626090281	0.08294444062936966	0.8368219610401129	0.1149336136999195	0.306519075374933
		N	44	44	45	44	44	44	44	44	44	44	43

			Motor Fitness	Muscular strenght		Screen time				Physical activity			
nPostcentral_gyrustilavuus_YHTEENSA	mri__t1__cross__volume__postcentral_gyrus_total_normalized	Pearson Correlation	-0.2164088698004152	0.1624156765775499	-0.04527764602565924	-0.03695805849397659	-0.2226340515855304	0.1793159792020515	-0.2220074052334076	-0.2496165722723819	-0.02749900230864189	0.02050991842024753	-0.1741129144483179
		Sig. (2-tailed)	0.1582587816904764	0.2921840451198695	0.7677371410144957	0.8117448178679145	0.146341769336753	0.2441506157013788	0.1475105060131808	0.1022408894353124	0.8593592059850376	0.8948698985053485	0.2641455068073272
		N	44	44	45	44	44	44	44	44	44	44	43
nPosterior_orbital_gyrustilavuus_YHTEENSA	mri__t1__cross__volume__posterior_orbital_gyrus_total_normalized	Pearson Correlation	-0.0006315511667512372	0.09380163283129096	-0.005745710455899503	0.07946989561191035	-0.09514222753461657	0.0940466431720312	-0.06000506808056088	-0.2217707824551272	-0.1612167248279354	-0.09567673624233539	-0.1048938728866658
		Sig. (2-tailed)	0.9967537104662915	0.544757160254005	0.9701189866949718	0.6081050129440467	0.5389973603011672	0.5437022887150169	0.6988161850272556	0.1479536065484127	0.2958091068258831	0.5367091019589082	0.503229484075212
		N	44	44	45	44	44	44	44	44	44	44	43
nPlanum_polaretilavuus_YHTEENSA	mri__t1__cross__volume__planum_polare_total_normalized	Pearson Correlation	-0.1257311337738897	0.06362604200749185	-0.1605830581307448	0.05191701602245746	0.009065591289780848	0.005108607304747615	0.05820159689433239	-0.04720961318861819	0.0823164868101411	0.2209545080048269	-0.08243061562072376
		Sig. (2-tailed)	0.416085669360118	0.6815759433017151	0.2919890356049528	0.7378587399728247	0.9534265836231223	0.9737453140239556	0.7074597384938163	0.7608934804617213	0.5952735601341707	0.1494896868376118	0.5992346162956895
		N	44	44	45	44	44	44	44	44	44	44	43
nPrecentral_gyrustilavuus_YHTEENSA	mri__t1__cross__volume__precentral_gyrus_total_normalized	Pearson Correlation	-0.1768217032945837	0.2583003713832553	0.04768064784086056	0.1457700111316809	0.2213424837348138	0.1255200234360714	-0.1412394997049674	0.07675049092873595	0.2677039498755781	0.1853823397998559	-0.07772168972637136
		Sig. (2-tailed)	0.2508811225867073	0.09047276496301551	0.7557794980372814	0.3450936309902228	0.1487581311870408	0.4168751060548772	0.3604533286295195	0.6204728251228315	0.07894342217837773	0.2282947764558127	0.6203265280596046
		N	44	44	45	44	44	44	44	44	44	44	43
nPlanum_temporaletilavuus_YHTEENSA	mri__t1__cross__volume__planum_temporale_total_normalized	Pearson Correlation	-0.07958711513900575	0.1606454263725303	-0.08175825768277553	0.1553287974503454	0.07048738390068796	0.1098833106465013	0.05787735699474415	-0.1865126528455743	-0.2015881635469081	0.2422707356206197	0.02465119749210686
		Sig. (2-tailed)	0.6075742886267975	0.2975466051130387	0.5934018742012969	0.3140306936440507	0.6493492682917936	0.4776702381030999	0.7090176319053354	0.225420447109544	0.1894499968271654	0.1130832860956928	0.8753164668803146
		N	44	44	45	44	44	44	44	44	44	44	43
nSubcallosal_areatilavuus_YHTEENSA	mri__t1__cross__volume__subcallosal_area_total_normalized	Pearson Correlation	0.04797437288282127	0.05537013468943359	0.008139281442184594	0.04385083854979096	0.08038452796533188	-0.05528434682610473	-0.1709359371421922	-0.05251256876706622	0.08595989579098998	0.09051136043447301	-0.1752964274515948
		Sig. (2-tailed)	0.757136762140612	0.721103416176634	0.9576806446106908	0.7774560853742465	0.6039692143438075	0.7215181567513169	0.2672541169439376	0.7349601522498562	0.5790265819926995	0.5590172959745487	0.260861909197463
		N	44	44	45	44	44	44	44	44	44	44	43
nSuperior_frontal_gyrustilavuus_YHTEENSA	mri__t1__cross__volume__superior_frontal_gyrus_total_normalized	Pearson Correlation	-0.03879368944293584	0.1119334175886119	-0.03692783367280745	0.2308345465732558	.407**	-0.1514000566384894	0.2065663070539958	0.2510880951347464	-0.02395690081575719	0.09697018406055273	0.1980188319288269
		Sig. (2-tailed)	0.8025763491683894	0.4694430773413377	0.8096863763141989	0.1316707805822818	0.006043790351557931	0.3265763551787292	0.1785213873562559	0.1001681653090787	0.8773264172349455	0.5311913484096201	0.2030600167686425
		N	44	44	45	44	44	44	44	44	44	44	43
nSupplementary_motor_cortextilavuus_YHTEENSA	mri__t1__cross__volume__supplementary_motor_cortex_total_normalized	Pearson Correlation	-0.2194816068425902	.405**	-0.03808089242302541	.313*	.443**	-0.01107162799367662	0.05410340343514979	0.1488249340047088	-0.05858493657743281	-0.06271970779322793	0.1036219779952982
		Sig. (2-tailed)	0.1522910373214716	0.006392522703565953	0.803859062229804	0.03876332992157118	0.002573091129043722	0.943136112828755	0.7272353906861395	0.3349673314229864	0.7056194071392554	0.6858765985222525	0.5084483710041099
		N	44	44	45	44	44	44	44	44	44	44	43
nSupramarginal_gyrustilavuus_YHTEENSA	mri__t1__cross__volume__supramarginal_gyrus_total_normalized	Pearson Correlation	-0.09598296978074848	0.1234608849292185	-0.2510279983288898	-0.1299807588127896	0.02351861542452963	-0.1711768522868853	-0.002159298357504925	0.2742773968773058	0.2919719143157073	0.1617348810810603	0.1323809960098218
		Sig. (2-tailed)	0.53540022627779	0.4246196767010243	0.09624977003681177	0.4003761559353194	0.879554036724724	0.2665703629371718	0.9889011234826631	0.07159236346660802	0.05446709704805897	0.2942389033602707	0.3974222828832997
		N	44	44	45	44	44	44	44	44	44	44	43
nSuperior_occipital_gyrustilavuus_YHTEENSA	mri__t1__cross__volume__superior_occipital_gyrus_total_normalized	Pearson Correlation	-0.04257299460545848	0.1033289180857822	0.1424383377852403	0.1567057118639789	0.06286311682935598	0.0912468843471723	0.1455515000394863	0.1101997622709826	0.04568786251835297	-0.08322430723468885	0.05835527560563993
		Sig. (2-tailed)	0.7837834450665528	0.5044752143790421	0.3506194516063341	0.309707104450643	0.6851954492653621	0.5558144089486741	0.3458250751988611	0.4763953663348148	0.7683848617401126	0.5912066169942859	0.7101145460571591
		N	44	44	45	44	44	44	44	44	44	44	43
nSuperior_parietal_lobuletilavuus_YHTEENSA	mri__t1__cross__volume__superior_parietal_lobule_total_normalized	Pearson Correlation	-0.2180632477166244	0.2836610650726606	-0.2445267942494053	0.188841821119154	-0.0716243794862086	.362*	-0.03942690826556146	0.1223234160937254	0.139354524653449	-0.145806008928257	0.03347570213590297
		Sig. (2-tailed)	0.1550248649702042	0.06204721085263253	0.1054777527868937	0.2195762691861027	0.6440675859291668	0.01586689510012846	0.7994196211582083	0.4289322833316689	0.3669638129708552	0.3449732232212613	0.8312442662936734
		N	44	44	45	44	44	44	44	44	44	44	43
nSuperior_temporal_gyrustilavuus_YHTEENSA	mri__t1__cross__volume__superior_temporal_gyrus_total_normalized	Pearson Correlation	-0.1287547295378271	0.2038662963259593	-0.139658091148998	-0.03648751976927566	-0.00391281525323584	-0.1374241408088676	0.1905077945445606	0.09001944648129216	-0.02216500656541374	0.2064334927080128	0.00999559411098582
		Sig. (2-tailed)	0.4048727426386409	0.1843912509640145	0.3601985292915415	0.8140990012360315	0.9798894067762197	0.3737038226970668	0.215460974616545	0.5611641757594786	0.8864395880671994	0.1788069559110599	0.9492763205057415
		N	44	44	45	44	44	44	44	44	44	44	43
nTemporal_poletilavuus_YHTEENSA	mri__t1__cross__volume__temporal_pole_total_normalized	Pearson Correlation	-0.01587484434826498	0.1698233119475517	-0.02720698060260818	-0.185240434504755	-0.1825740212089727	0.06125670670177599	-0.11655945196884	-0.0956081264241451	0.02404551622759551	0.09554790222727574	-0.1252183601465691
		Sig. (2-tailed)	0.9185370506126024	0.2704269858181438	0.8591888270351764	0.2286574037801576	0.2355447697202773	0.6928393843887907	0.4511593743467173	0.5370025603569074	0.8768761357380566	0.5372602157694254	0.423668926878598
		N	44	44	45	44	44	44	44	44	44	44	43
nTriangular_part_of_the_inferior_frontal_gyrustilavuus_YHTEENSA	mri__t1__cross__volume__triangular_part_of_the_inferior_frontal_gyrus_total_normalized	Pearson Correlation	-0.2169050946112254	0.2292247381427871	-0.03840155378490965	0.2056868949807871	0.1324155243886863	0.0731337137732486	0.2078815848685941	-0.03212002291288221	0.04317901647419049	0.1679494521689527	0.007475914405900893
		Sig. (2-tailed)	0.1572836548875291	0.1344604192741939	0.8022403603988452	0.1804183361392801	0.3915326587715098	0.6370829886493683	0.1757110038843487	0.8360256436086274	0.7807809279166691	0.2758267053591123	0.9620518433145647
		N	44	44	45	44	44	44	44	44	44	44	43
nTransverse_temporal_gyrustilavuus_YHTEENSA	mri__t1__cross__volume__transverse_temporal_gyrus_total_normalized	Pearson Correlation	-0.1814624114840648	0.2253889830896818	-0.008990469468136735	.387**	0.171589654326569	.362*	-0.06100527788844127	-0.08459844501909089	-0.2416273260474613	0.05853979067437745	0.1408848578522043
		Sig. (2-tailed)	0.2384574211628273	0.1412845464684402	0.953259635666849	0.00938911240484984	0.2654014664285699	0.0156760471879584	0.6940385382640589	0.5850741611507906	0.1140730039875018	0.7058360569496889	0.3675208953236104
		N	44	44	45	44	44	44	44	44	44	44	43
nOtsalohkotilavuus_YHTEENSA	mri__t1__cross__volume__frontal_lobe_total_normalized	Pearson Correlation	-0.1229103588969056	0.276205739123603	-0.05122043638483814	0.2392906309912014	.333*	-0.06225530128998791	0.09587576829988165	0.1761849266133829	-0.0372183804465669	0.1544447747717799	0.1272037579771795
		Sig. (2-tailed)	0.4267038872760287	0.06954174489589345	0.7382681055586124	0.1177227304463374	0.02725096506121751	0.6880840742902016	0.5358582407258103	0.2526191927020607	0.8104429740044587	0.3168266431469585	0.4162976285581541
		N	44	44	45	44	44	44	44	44	44	44	43

			Motor Fitness	Muscular strenght		Screen time				Physical activity			
nOhimolohkotilavuus_YHTEENSA	mri__t1__cross__volume__temporal_lobe_total_normalized	Pearson Correlation	-0.1713767737111812	0.2793331176332302	-0.0376016159118497	-0.1646559713844793	-0.251396999205477	-0.01888807578830537	0.08844088205168513	-0.1385112769836528	-0.01055176543776215	0.2033777474466713	-0.2218374408843994
		Sig. (2-tailed)	0.266003836828848	0.06631515156019363	0.8062799664025296	0.2854878622899328	0.09973718557481726	0.9031419929131033	0.5680794097706028	0.3698990120751144	0.945802067925855	0.1854679029558574	0.1528019390887574
		N	44	44	45	44	44	44	44	44	44	44	43
nPäälakilohkotilavuus_YHTEENSA	mri__t1__cross__volume__parietal_lobe_total_normalized	Pearson Correlation	-.302*	.380*	-.308*	-0.02899568316009304	-0.2295380057046109	0.1468664512011505	-0.05881097745330698	-0.03321203532592933	0.1911067067273923	0.06184230827756711	-0.1419518486368545
		Sig. (2-tailed)	0.04635904101594538	0.01084855476831703	0.03965371425068264	0.8517878415947794	0.1339141358807711	0.3414377778710231	0.7045350100589838	0.8305310798848207	0.2139947105554658	0.6900493196343757	0.3638669638461984
		N	44	44	45	44	44	44	44	44	44	44	43
nTakaraivolohkotilavuus_YHTEENSA	mri__t1__cross__volume__occipital_lobe_total_normalized	Pearson Correlation	-0.1868448743823679	0.0812497805285187	-0.02965287481454979	0.09039997760165602	0.003105065213697323	0.2400810341915265	-0.02540697755025104	-0.1231558031503674	0.1550495076092214	0.04722295125021509	-0.2198229391219913
		Sig. (2-tailed)	0.2245803812584057	0.6000679521012611	0.8466751762544821	0.5595030717308749	0.9840403752348518	0.116478434726516	0.8699630523602981	0.425773962269915	0.3149123242834437	0.7608279132310769	0.1566607928857103
		N	44	44	45	44	44	44	44	44	44	44	43
nSisempi_ohimolohkotilavuus_YHTEENSA	mri__t1__cross__volume__medial_temporal_lobe_total_normalized	Pearson Correlation	0.07121169043082358	-0.1576727426732993	-0.06473206587888307	-0.215814840126999	-.297*	-0.1404976285795956	0.1604222212649297	-0.052000633106336	-0.1488527793267212	0.06817369201443506	-0.08617306568961887
		Sig. (2-tailed)	0.6459826647638144	0.3066933396883757	0.6726892915054495	0.1594318967448881	0.04997393489686337	0.3630072805469933	0.2982272241426686	0.7374515531624833	0.33487588847548	0.6601494453364075	0.582698547611686
		N	44	44	45	44	44	44	44	44	44	44	43
nIsoaivojen_harmaa_ja_valkean_ainetilavuus_DX	mri__t1__cross__volume__cerebral_gray_and_white_matter_right_normalized	Pearson Correlation	-0.118855307825178	0.1861358814279665	-0.1169933851380583	0.2040824598939426	0.1168873480693572	0.2128258980029009	-0.06057006261531833	-0.1175201486624164	-0.06385870365475124	0.03651201882719662	-0.04385497625603887
		Sig. (2-tailed)	0.4422317073986504	0.2263757771050336	0.444054468230874	0.1839163006084291	0.4498783336938482	0.1654308913577516	0.696115979955687	0.4474117043363065	0.6804735382705714	0.8139763844570364	0.78006325499268
		N	44	44	45	44	44	44	44	44	44	44	43
nIsoaivojen_harmaa_ja_valkean_ainetilavuus_SIN	mri__t1__cross__volume__cerebral_gray_and_white_matter_left_normalized	Pearson Correlation	-.334*	0.2928613618648426	-0.06965827222883107	0.0864365598037371	-0.1281165387207471	0.2610762684307293	0.00618277649997319	-0.1562993962676444	-0.07616584192359047	0.005373319879487441	-0.112325398631026
		Sig. (2-tailed)	0.02692228801187169	0.05370202055236366	0.6493355039566577	0.576915943628479	0.4072248408987973	0.08694135813842786	0.968227400792733	0.3109789978526941	0.6231455205535659	0.9723853863817035	0.473289713213627
		N	44	44	45	44	44	44	44	44	44	44	43
nDorsol_lateral_prefrontaltilavuus_DX	mri__t1__cross__volume__dorso_lateral_prefrontal_right_normalized	Pearson Correlation	-0.04903307648784655	0.2015116355044343	-0.06151114840037802	.303*	.395**	0.01454122266780573	0.08223681796855742	0.09327500937142741	-0.1319398987077055	0.1162719544708616	0.09825680457074357
		Sig. (2-tailed)	0.7519452375085816	0.1896216264699538	0.6881268660310295	0.04523823428134453	0.007989990877690306	0.9253604704420167	0.5956310549673427	0.5470278042190688	0.393251172974268	0.4522842186518845	0.5307597596220915
		N	44	44	45	44	44	44	44	44	44	44	43
nDorsol_lateral_prefrontaltilavuus_SIN	mri__t1__cross__volume__dorso_lateral_prefrontal_left_normalized	Pearson Correlation	-0.1623828923305227	0.2499292349199709	-0.1219605926666383	0.2838545653986155	.359*	-0.07908410828963937	0.2305557769565887	0.1859053607136522	-0.07539977551447266	0.109566376798479	0.1618621354521056
		Sig. (2-tailed)	0.2922827849897597	0.1017977575650277	0.4248125071018527	0.06186163495556488	0.01676325708539268	0.6098531119092605	0.1321507399919042	0.2269616490371431	0.626654806834736	0.4789488528538085	0.2997440923928134
		N	44	44	45	44	44	44	44	44	44	44	43
nDorsol_lateral_prefrontaltilavuus_YHTEENSA	mri__t1__cross__volume__dorso_lateral_prefrontal_total_normalized	Pearson Correlation	-0.1153936544037248	0.2402518187824945	-0.09771743026153447	.306*	.386**	-0.03483049945251079	0.1696385008273524	0.1500314529112669	-0.107820786422829	0.11909378166314	0.1387555874563115
		Sig. (2-tailed)	0.4557300416252714	0.1162108892817732	0.5230874509590554	0.04368700059021826	0.009627525636011467	0.8224023657429635	0.2709564111303662	0.3310193924758322	0.4860232971429976	0.4413099983357446	0.3748781028147696
		N	44	44	45	44	44	44	44	44	44	44	43
nAnteroLateralTemporaltilavuus_DX	mri__t1__cross__volume__antero_lateral_temporal_right_normalized	Pearson Correlation	0.009248898946344791	0.1618827747250407	-0.07648424399579981	-0.08684729631762819	-0.221440573341192	0.09963084221636336	0.05801508312727913	-0.2808616661888957	-0.07111633289469894	0.0874021039446805	-.318*
		Sig. (2-tailed)	0.9524859249619244	0.2937917212031536	0.6175212134616884	0.5751000363040862	0.1485735942001959	0.5199290801123114	0.7083557477894059	0.06478195301316049	0.6464254927126629	0.5726513197339633	0.03743623955704125
		N	44	44	45	44	44	44	44	44	44	44	43
nAnteroLateralTemporaltilavuus_SIN	mri__t1__cross__volume__antero_lateral_temporal_left_normalized	Pearson Correlation	-0.1992034268715663	0.2799004309900072	0.05838455237805715	-0.2291265280589418	-0.1997736116663001	-0.1418976370974491	0.04958964597426484	-0.07118940495912485	0.1858498231386276	0.1456341521773677	-0.219738111665536
		Sig. (2-tailed)	0.1948502425237361	0.065742785651733	0.7032333082261307	0.1346320217299592	0.1935492678424593	0.3581967595630566	0.7492203245523569	0.6460861447659297	0.2271029542569302	0.3455482934903391	0.1568248295597847
		N	44	44	45	44	44	44	44	44	44	44	43
nAnteroLateralTemporaltilavuus_YHTEENSA	mri__t1__cross__volume__antero_lateral_temporal_total_normalized	Pearson Correlation	-0.08210019952126375	0.2190575299723681	-0.01136611129241708	-0.1612203241116412	-0.2193157918557097	-0.01416263206979802	0.04863324524161814	-0.1964005630218482	0.04911791084372661	0.1210230133518758	-0.2929192424886023
		Sig. (2-tailed)	0.5962443170649114	0.153104694425622	0.9409284749024387	0.2957981810657377	0.1526088005397065	0.9272984995746815	0.753904617671808	0.2013353518147128	0.751529702840219	0.4338925695105055	0.05661822363796479
		N	44	44	45	44	44	44	44	44	44	44	43
nOrbitofrontaltilavuus_DX	mri__t1__cross__volume__orbito_frontal_right_normalized	Pearson Correlation	-0.07127417788329762	0.1834709427408275	-0.04093399778797588	0.00204696543720775	0.1648133983005692	-.301*	-0.001279688633777066	0.1438614969918672	-0.01992818585554454	0.0945098896831951	0.09067927533516175
		Sig. (2-tailed)	0.6456925467433112	0.2332124043938241	0.7894858240345412	0.9894784858666404	0.2850211083690033	0.04705125802427755	0.993422218609898	0.3515143035929262	0.8978357636075621	0.5417105004192366	0.5630646580085709
		N	44	44	45	44	44	44	44	44	44	44	43
nOrbitofrontaltilavuus_SIN	mri__t1__cross__volume__orbito_frontal_left_normalized	Pearson Correlation	0.1598059067247945	-0.1335408071176182	-0.05850313003947599	-0.01767479297347606	0.02176541809925117	-0.1691973059995951	-0.07160571823643713	-0.02340328025063032	0.02922337495397031	0.103249056575931	-0.0856696921426576
		Sig. (2-tailed)	0.3001117508360969	0.3874843584707206	0.7026582783374886	0.9093369379099093	0.8884738224847707	0.2722230603939188	0.6441541342224213	0.8801403901418053	0.8506371366728762	0.5048064622621761	0.5849107359770082
		N	44	44	45	44	44	44	44	44	44	44	43
nOrbitofrontaltilavuus_YHTEENSA	mri__t1__cross__volume__orbito_frontal_total_normalized	Pearson Correlation	0.05111189325377352	0.03148912958251388	-0.05528854024819394	-0.005407547972931233	0.1145379393927039	-0.2761505122322877	-0.04407824413279005	0.07667807571113042	0.004597922233235126	0.1151004521759051	0.009503921094607919
		Sig. (2-tailed)	0.7417830275644758	0.839203578750778	0.7183042054355406	0.9722095494142945	0.459100901873305	0.06959982006273065	0.7763315427636437	0.6208036068634596	0.9763690990376421	0.4568835191664951	0.9517683619541945
		N	44	44	45	44	44	44	44	44	44	44	43
nMediotemporaltilavuus_DX	mri__t1__cross__volume__medio_temporal_right_normalized	Pearson Correlation	-0.02441918142920507	0.01148833183789126	0.1819094847198685	-.362*	-.327*	-0.170320908924912	-0.04622665795689983	0.1589021699273237	0.06174457897366053	0.06025597327693837	0.008969035087725605

			Motor Fitness	Muscular strenght		Screen time				Physical activity			
		Sig. (2-tailed)	0.874977854698776	0.9409996172356054	0.2317135678743509	0.01579375244915229	0.03007291339816793	0.2690049276496834	0.765730017929427	0.3028889436152917	0.6905146642806591	0.6976166106668769	0.9544799432510072
		N	44	44	45	44	44	44	44	44	44	44	43
nMediotemporaltilavuus_SIN	mri__t1__cross__volume__medio_temporal_left_normalized	Pearson Correlation	-0.2910009761299859	0.144371107778387	-0.01834101949816674	-.353*	-.483**	-0.07204295250058797	0.1061091586185487	0.03743434698017401	-0.1060073613788751	0.03129495473023864	-0.0465073043690474
		Sig. (2-tailed)	0.05531224675513827	0.3497927640545956	0.904813461901308	0.0186675919548483	0.000885980686288805	0.6421275261826837	0.4930126254734815	0.8093633906700085	0.4934299368720756	0.8401821872918028	0.7671212503135605
		N	44	44	45	44	44	44	44	44	44	44	43
nMediotemporaltilavuus_YHTEENSA	mri__t1__cross__volume__medio_temporal_total_normalized	Pearson Correlation	-0.1784082614384158	0.08051090637219552	0.1105322324880471	-.452**	-.500**	-0.1617018969848134	0.0268028573076845	0.1356604982292524	-0.01657551148723551	0.05892500957946786	-0.01794772742529406
		Sig. (2-tailed)	0.2465856935764714	0.603398713364241	0.4697862120912628	0.002054540362463316	0.0005488771881526945	0.294338696461401	0.8628851374600427	0.3799257247434258	0.9149544009207273	0.7039881774978338	0.9090537211436067
		N	44	44	45	44	44	44	44	44	44	44	43
nCingulatetilavuus_DX	mri__t1__cross__volume__cingulate_right_normalized	Pearson Correlation	-0.08893568532787748	0.125686526061407	0.104857392971339	-0.006883668217642402	-0.04896776496005121	0.07547198124432662	-0.09852159062674033	0.0759036017636196	0.1824382733112423	-0.09776903100095545	-0.06662458161543881
		Sig. (2-tailed)	0.565907597085214	0.4162524067945783	0.4930257551741762	0.9646277556478383	0.7522651929116404	0.6263236889233994	0.5246099503829367	0.6243458978122266	0.2358991512148896	0.5277974350328832	0.6712121226009334
		N	44	44	45	44	44	44	44	44	44	44	43
nCingulatetilavuus_SIN	mri__t1__cross__volume__cingulate_left_normalized	Pearson Correlation	-0.007107849280851524	0.04241256941170451	0.05955791625311029	-0.1038459716162172	-0.08973475232270453	-0.0328421198256837	0.003558102160383507	0.0807375417360272	-0.2124873946894463	0.0740312013177565	0.187707038053364
		Sig. (2-tailed)	0.9634765480443084	0.7845787884704215	0.6975504586941049	0.5023332665418777	0.5624084318353366	0.8323914641818201	0.9817121932276843	0.6023762150007748	0.1661204690459772	0.6329444085752162	0.2280657495654004
		N	44	44	45	44	44	44	44	44	44	44	43
nCingulatetilavuus_YHTEENSA	mri__t1__cross__volume__cingulate_total_normalized	Pearson Correlation	-0.05421855898545796	0.09750602883328097	0.1050097835670369	-0.07167090845862564	-0.08158889361653139	0.01667918680142139	-0.05107039938184307	0.1033805549841236	-0.0205649552837964	-0.008712785117097024	0.08810482275232959
		Sig. (2-tailed)	0.7266772444565577	0.5289136262593181	0.492394024247409	0.6438518112531724	0.5985419576638064	0.9144244282093993	0.741985451264713	0.5042610947616875	0.8945893750520719	0.9552372130893163	0.5742441139366952
		N	44	44	45	44	44	44	44	44	44	44	43
nAivokammiottilavuus_YHTEENSA	mri__t1__cross__volume__ventricles_total_normalized	Pearson Correlation	0.07744244873136406	-0.1019200266901967	0.1826376030572674	-0.1752297038334852	-0.02903422136355364	-0.2608394238510434	-0.05430930358216868	0.275413913427778	.443**	0.1274568040699163	0.01935728483439227
		Sig. (2-tailed)	0.6173158046379557	0.5103351587842879	0.2298188913705081	0.2552415870507229	0.8515930564281495	0.08723841457773529	0.7262375138877126	0.07037808305235523	0.00258208762570471	0.4096645754333619	0.9019445564444302
		N	44	44	45	44	44	44	44	44	44	44	43
nHippokampus_Asymmetria_	mri__t1__cross__asymmetry__hippocampus_normalized	Pearson Correlation	.360*	-0.2157787067719526	-0.05454369886965149	0.09898536202090298	0.1201081560167303	0.0780531431090196	-0.2484644412877501	-.338*	-0.04668816622989606	-0.1168007780805595	-0.1949399274469113
		Sig. (2-tailed)	0.01635673953647814	0.1595034584006647	0.7219460617721883	0.5226503793204856	0.4374012814278806	0.6145351920728257	0.1038865640279257	0.02480349632021485	0.7634581019028183	0.4502163564024045	0.2103156860795013
		N	44	44	45	44	44	44	44	44	44	44	43
nSisempi_ohimolohkoLaskennallinen_MTA_DX	mri__t1__cross__mta__medial_temporal_lobe_right_normalized	Pearson Correlation	-0.251891103496411	.361*	0.1571492602018817	0.03106226959269004	-0.08869280469420146	0.1593594806329144	-0.1446348716040973	-0.07621431158752574	0.2471622560073858	-0.07332486478967351	-0.2396716803967388
		Sig. (2-tailed)	0.09905078145282026	0.01597421629539197	0.3025655192835669	0.841355194713929	0.5669731794788183	0.3014815713795617	0.3489037602062375	0.6229237611750951	0.1057709053598492	0.6362006144830565	0.1216271003405891
		N	44	44	45	44	44	44	44	44	44	44	43
nSisempi_ohimolohkoLaskennallinen_MTA_SIN	mri__t1__cross__mta__medial_temporal_lobe_left_normalized	Pearson Correlation	-0.1044213589032984	0.1874515201731493	0.1422792468863943	-0.01241571930282166	0.0637538417783799	-0.02380918409882695	-0.2620292617117211	-0.0923623169641114	.301*	-0.1044060807789532	-0.2337393221669435
		Sig. (2-tailed)	0.4999551268730068	0.2230519659823301	0.3511633431815429	0.9362462694542826	0.6809703169595255	0.8780770951744707	0.08575403553455216	0.5509737226303419	0.04719792246599468	0.5000181985536918	0.1314137093644973
		N	44	44	45	44	44	44	44	44	44	44	43
nIsoaivojen_kortikaalinen_harmaa_aine_LaskennallinenGCA_YHTEENSA	mri__t1__cross__gca__cerebral_cortex_total_normalized	Pearson Correlation	.c	.c	.c	.c	.c	.c	.c	.c	.c	.c	.c
		Sig. (2-tailed)											
		N	44	44	45	44	44	44	44	44	44	44	43
nIsoaivojen_kortikaalinen_harmaa_aine_LaskennallinenGCA_DX	mri__t1__cross__gca__cerebral_cortex_right_normalized	Pearson Correlation	-0.2666252037800611	0.1972866024343124	0.1056238648234097	-0.1776763305855807	-0.1414378590714078	-0.09441109002275368	-0.04280576096680654	0.1308049045549311	0.2141508309576987	-0.08093080919324465	0.1122900522368877
		Sig. (2-tailed)	0.0802043504874653	0.1992690973509811	0.4898526169014473	0.2485611028718556	0.3597723064094847	0.542135007711098	0.7826298415069877	0.3973698454648882	0.1627517833752843	0.6015048556116912	0.4734298439523286
		N	44	44	45	44	44	44	44	44	44	44	43
nIsoaivojenKortikaalinenHarmaaaineLaskennallinenGCA_SIN	mri__t1__cross__gca__cerebral_cortex_left_normalized	Pearson Correlation	0.2494719299292429	-0.2499526809590829	0.05874557326603342	.331*	.301*	-0.1086996517794242	0.1157652661716887	-0.1108322753675834	-0.05361213891563418	-0.09261686103727872	0.01041726008397261
		Sig. (2-tailed)	0.1024463885382452	0.1017645873449821	0.701483086467939	0.02819765831444427	0.04682114773700784	0.482454682827669	0.4542703676461677	0.4738525885666311	0.7296180595413068	0.5498718756600298	0.9471394888844928
		N	44	44	45	44	44	44	44	44	44	44	43
nOtsalohkoLaskennallinenGCA_DX	mri__t1__cross__gca__frontal_lobe_right_normalized	Pearson Correlation	0.09153456777698216	-0.2476093965551773	0.1438532330312499	-0.1209382929414544	-.341*	0.07965407925832207	0.08591611102671269	0.08388125217477561	0.06037015116414596	-0.05871415359579615	0.09253182632288975
		Sig. (2-tailed)	0.5545640256676081	0.1051209431513288	0.3458049762268773	0.434216833658798	0.02368618064206312	0.6072711906243236	0.5792206333016081	0.5882712717845707	0.6970709685442863	0.7049994372308045	0.555083118884229
		N	44	44	45	44	44	44	44	44	44	44	43
nOtsalohkoLaskennallinenGCA_SIN	mri__t1__cross__gca__frontal_lobe_left_normalized	Pearson Correlation	0.1772013681900862	-0.2356245020004379	0.02446186546750481	0.2572704922732527	0.2808570519588656	-0.09753669455284458	0.02349122150350213	-0.1172839734518399	-0.1020782616949255	-0.1136712296573166	-0.01363618930840114
		Sig. (2-tailed)	0.2498486710498108	0.1236259282752439	0.8732739498640648	0.09181086384892358	0.06478653849186894	0.5287834201742576	0.879693299147586	0.4483314371835767	0.5096753128061572	0.4625287500305877	0.9308404031818129
		N	44	44	45	44	44	44	44	44	44	44	43
nOhimolohkoLaskennallinenGCA_DX	mri__t1__cross__gca__temporal_lobe_right_normalized	Pearson Correlation	-0.1567023607457697	0.1365861702961051	0.06521219994500776	-0.03222421837430322	-0.05403444612552041	0.05986777057904399	-0.2635868274227426	-0.006427869316729193	0.1923146345577849	-0.01638863163693393	0.004354416455865227
		Sig. (2-tailed)	0.3097175809228743	0.3766524586927751	0.6703992186892146	0.8355010370525797	0.7275696859538714	0.699472909709026	0.08384071286008295	0.9669685766268721	0.2110585836937171	0.9159097946779742	0.97789143499189
		N	44	44	45	44	44	44	44	44	44	44	43

			Motor Fitness	Muscular strenght		Screen time				Physical activity			
nOhimolohkoLaskennallinenGCA_SIN	mri__t1__cross__gca__temporal_lobe_left_normalized	Pearson Correlation	0.2082195856565897	-0.26321724699173	0.1351084349922508	-0.01407965684559654	-0.1997839678010463	0.1528297637457704	0.1195709888814849	0.05453711230380946	-0.1899574534157325	-0.04325061419097147	0.2344341015187178
		Sig. (2-tailed)	0.1749939548009881	0.08429166531110975	0.3762118293556259	0.9277233109101019	0.1935256955226299	0.3219750324425557	0.4394687655479816	0.7251339807170698	0.2168144627411328	0.7804264062406456	0.13023806924485
		N	44	44	45	44	44	44	44	44	44	44	43
nPäälakilohkoLaskennallinenGCA_DX	mri__t1__cross__gca__parietal_lobe_right_normalized	Pearson Correlation	-0.1596366104571615	0.1319748500733777	0.05933312921938278	0.05984531105579216	-0.005520329043351363	0.2404837671015655	-0.09684926645018077	-0.2254559512356326	-0.06645258109205472	-0.08186382913301421	-0.1507656213668545
		Sig. (2-tailed)	0.3006307505724821	0.3931247382065484	0.6986378948455256	0.6995803592743198	0.9716301784187907	0.1158482722795529	0.5317059966141989	0.141163245901624	0.6682281408169723	0.5973060065916964	0.3345272249619813
		N	44	44	45	44	44	44	44	44	44	44	43
nPäälakilohkoLaskennallinenGCA_SIN	mri__t1__cross__gca__parietal_lobe_left_normalized	Pearson Correlation	.380*	-0.2967801549871178	-0.1692962218196361	0.1248500568559672	0.2631426351265516	-0.1524045797230866	-0.03691709513702685	-0.2366172521785153	-0.1643278750285982	-0.05687184486811756	-0.08550610481987175
		Sig. (2-tailed)	0.01095737853616959	0.0504335278537456	0.2662396334611331	0.4193860434445915	0.08438293382722306	0.3233391496192443	0.8119496330739397	0.1220058633461297	0.286462232370627	0.7138563104334873	0.5856304656938885
		N	44	44	45	44	44	44	44	44	44	44	43
nTakaraivolohkoLaskennallinenGCA_DX	mri__t1__cross__gca__occipital_lobe_right_normalized	Pearson Correlation	-0.1759260005331218	0.1385864260522939	0.08234453134580462	-0.08067838941020106	-0.0207572771841195	-0.0638321486258798	-0.04413523384245801	0.1532143228554682	0.0892762865026957	0.0243149685597144	0.1123821469191072
		Sig. (2-tailed)	0.25332823364642	0.369636862025812	0.5907466085202813	0.6026430167539562	0.8936092083592156	0.6805993289685692	0.7760497938299562	0.3207443746284626	0.5644148514498626	0.8755072040617733	0.4730647806514523
		N	44	44	45	44	44	44	44	44	44	44	43
nTakaraivolohkoLaskennallinenGCA_SIN	mri__t1__cross__gca__occipital_lobe_left_normalized	Pearson Correlation	0.2387796536830144	-0.2330179465949159	0.1711489138959585	-0.0808501494143561	0.04621569530568759	-0.1495748819172346	-0.1606061383251791	-0.1442770329497957	-0.1021422363372385	0.1855132023132586	-0.2014710109754541
		Sig. (2-tailed)	0.1185324716612109	0.1279565397392094	0.2609652702855072	0.6018684490312367	0.76578400864717	0.3325099468698108	0.2976663334886112	0.3501101738102663	0.5094086583550641	0.2279607180970683	0.1951355392094838
		N	44	44	45	44	44	44	44	44	44	44	43
nOtsalohkoLaskennallinenGCA_YHTEENSA	mri__t1__cross__gca__frontal_lobe_total_normalized	Pearson Correlation	0.1212487009357505	-0.2573124410411651	0.1640449822425342	-0.05025372001430534	-0.2688127643933859	0.04857172554558167	0.1249990474052448	0.0818635738522982	0.05761477547585764	-0.06931876525848259	0.09489714447615984
		Sig. (2-tailed)	0.4330294174960234	0.0917560630725591	0.2815715136745531	0.7459730253387309	0.07766359681638221	0.7542062319861569	0.4188269087125384	0.5973071536672326	0.7102801323478136	0.654795608329261	0.5449704321460979
		N	44	44	45	44	44	44	44	44	44	44	43
nOhimolohkoLaskennallinenGCA_YHTEENSA	mri__t1__cross__gca__temporal_lobe_total_normalized	Pearson Correlation	-0.07835406984444887	0.02075361065879947	0.06637792049888054	0.04347321725543621	-0.1107192828417777	0.197082467247314	-0.2118803832599512	-0.05875022648475781	0.09368695169587643	-0.1215140251187685	0.07755056069901978
		Sig. (2-tailed)	0.6131669709720498	0.8936278933103269	0.6648514500522336	0.7793244511310667	0.4743063037082016	0.1997438138351877	0.1673622479135163	0.7048263969239568	0.5452512466908398	0.4320158992288498	0.6210988690815087
		N	44	44	45	44	44	44	44	44	44	44	43
nPäälakilohkoLaskennallinenGCA_YHTEENSA	mri__t1__cross__gca__parietal_lobe_total_normalized	Pearson Correlation	-0.08108848958069528	-0.005444989649751363	0.01687426921135929	-0.0750899518708052	0.007399000745048281	-0.01164116685522072	-0.02620147218637604	-0.2305924192786189	-0.1255274023055787	-0.008297555720258558	-0.1360361673615189
		Sig. (2-tailed)	0.6007943492547927	0.9720172050795035	0.9123947591433186	0.6280764052921236	0.9619815460008846	0.9402161137011774	0.8659332393674233	0.1320875782222869	0.4168474987871197	0.9573684880892542	0.3844008198432658
		N	44	44	45	44	44	44	44	44	44	44	43
nTakaraivolohkoLaskennallinenGCA_YHTEENSA	mri__t1__cross__gca__occipital_lobe_total_normalized	Pearson Correlation	-0.01241196113478256	-0.009971986425457447	0.1225375683924051	-0.07134487167111458	0.05732828797017254	-0.153829587293466	-0.08384906876724926	0.0740794283318912	0.02893595116835724	0.09811201257354954	0.03904312899617368
		Sig. (2-tailed)	0.9362655277313621	0.9487759749520243	0.4226083208424352	0.6453643908075042	0.7116584481037403	0.3187815887747963	0.588414921577648	0.6327223313245828	0.8520897645799601	0.5263435490192165	0.8036908004570111
		N	44	44	45	44	44	44	44	44	44	44	43
nAnterior_vs_Posterior__indeksi	mri__t1__cross__anterior_vs_posterior_index_normalized	Pearson Correlation	0.05266316872243319	0.07979598983813509	0.1041117044001124	0.0007886450535454417	0.07278031605173069	-0.1177319017595929	0.08427170240624475	0.05466537062067058	-0.2343605814420849	0.00850199659154496	0.1367687597270447
		Sig. (2-tailed)	0.7342277482137651	0.6066290794797168	0.4961230156267843	0.995946224929929	0.6387156174232002	0.446587957893929	0.5865297530824869	0.724512923649284	0.1257122241479894	0.956319103475483	0.3818215556213386
		N	44	44	45	44	44	44	44	44	44	44	43
*. Correlation is significant at the 0.05 level (2-tailed).													
**. Correlation is significant at the 0.01 level (2-tailed).													
c. Cannot be computed because at least one of the variables is constant.													
PA, physical activity													
